# Complexity begets simplicity: self-supervised learning for palaeontological images with few or no labels

**DOI:** 10.1098/rsif.2025.1243

**Published:** 2026-10-21

**Authors:** Niall Rodgers

**Affiliations:** UK Centre for Astrobiology, School of Physics and Astronomy, The University of Edinburgh, Edinburgh, Midlothian, UK

**Keywords:** self-supervised learning, machine learning, palaeontology, fossil classification, unsupervised learning, microfossils

## Abstract

Palaeontology has seen widespread and growing use of machine learning (ML) to classify and analyse large datasets of fossils. However, palaeontology is a challenging field in which to apply ML. Datasets may be small or unlabelled, images may be complex and different from standard datasets and palaeontologists may lack specialist training and access to necessary computational resources. We show how these challenges can be addressed by using recent developments in self-supervised learning (SSL). Using a frozen DINOv3 feature extractor and a simple linear classifier, with reduced data, we can achieve comparable results to literature benchmarks using convolutional neural networks, the previous standard, when classifying fossil tracks, pollen, radiolaria, foraminifera and a dataset of diverse fossil images. In addition, the rich feature vectors generated by the model can be used for few-shot learning, unsupervised clustering, subject to the presence of imaging artefacts and quantification of disparity. Using state-of-the-art self-supervised methods increases accessibility by reducing code, compute and data required. It also maintains accuracy, while increasing reproducibility by reducing parameters.

## 1. Introduction

Machine learning (ML) is currently having profound effects on society and science; however, the most recent developments in ML methodology, using pre-trained self-supervised models, have yet to break into palaeontology [1–3]. Palaeontology is a challenging domain for ML for a variety of reasons. Datasets can be small, and labelling is time-consuming and requires specialist knowledge. Images are complex with potentially degraded objects, distinct from standard ML training data, which are difficult to segment from the background and each other. Palaeontologists may not have the mathematical training found in some disciplines of the natural sciences, making applying untested complex ML pipelines unappealing. Moreover, owing to the number of parameters and complexity of these pipelines, there are issues with reproducibility [3]. Ideally, palaeontologists could learn a single pipeline and apply it to diverse datasets, before potentially needing to resort to bespoke methods. Recent developments, however, present the possibility to partially ameliorate all of the above issues. State-of-the-art models [4], pre-trained on vast amounts of unlabelled data using the approach known as self-supervised learning (SSL) [4–8], allow robust rich feature vectors to be extracted from diverse complex images, in just a few lines of high-level Python code [9]. Using these frozen, off-the-shelf, models to extract features, it is then possible to obtain excellent performance in downstream tasks [4,6]. For palaeontology, this raises the possibility that complex convolutional neural network (CNN) pipelines could be replaced, in the first instance, with self-supervised feature extractors. The embedding quality also raises the possibility of using unsupervised learning to reveal structure in the data, either taxonomic or as an artefact detection tool, or to use feature properties as a proxy for morphological disparity.

In this work, we apply the state-of-the-art self-supervised feature extractor DINOv3 [4] to various image sets across scales related to palaeontology, including foraminifera [10,11], radiolaria [12], fossil tracks [13], pollen [14] and a dataset of diverse fossil images [15]. We show that we can accurately classify objects, with reduced data, using the extracted features and simple k-nearest neighbour (kNN) or logistic regression classification (§5). Furthermore, we show that applying nonlinear dimensionality reduction to the feature vectors can reveal the structure of the datasets and that unsupervised learning can find meaningful groupings in the data without labels (§6). Moreover, we propose that, under carefully controlled circumstances, the statistical properties of the set of extracted features may be used to understand the disparity of a dataset (§6 and supplementary material). This pipeline (figure 1) provides a generalizable framework that maintains accuracy, with less data, while reducing computational burden, length of code and number of parameters. This framework could improve adoption of ML in palaeontology while increasing reproducibility and the range of useable datasets.

**Figure 1.**
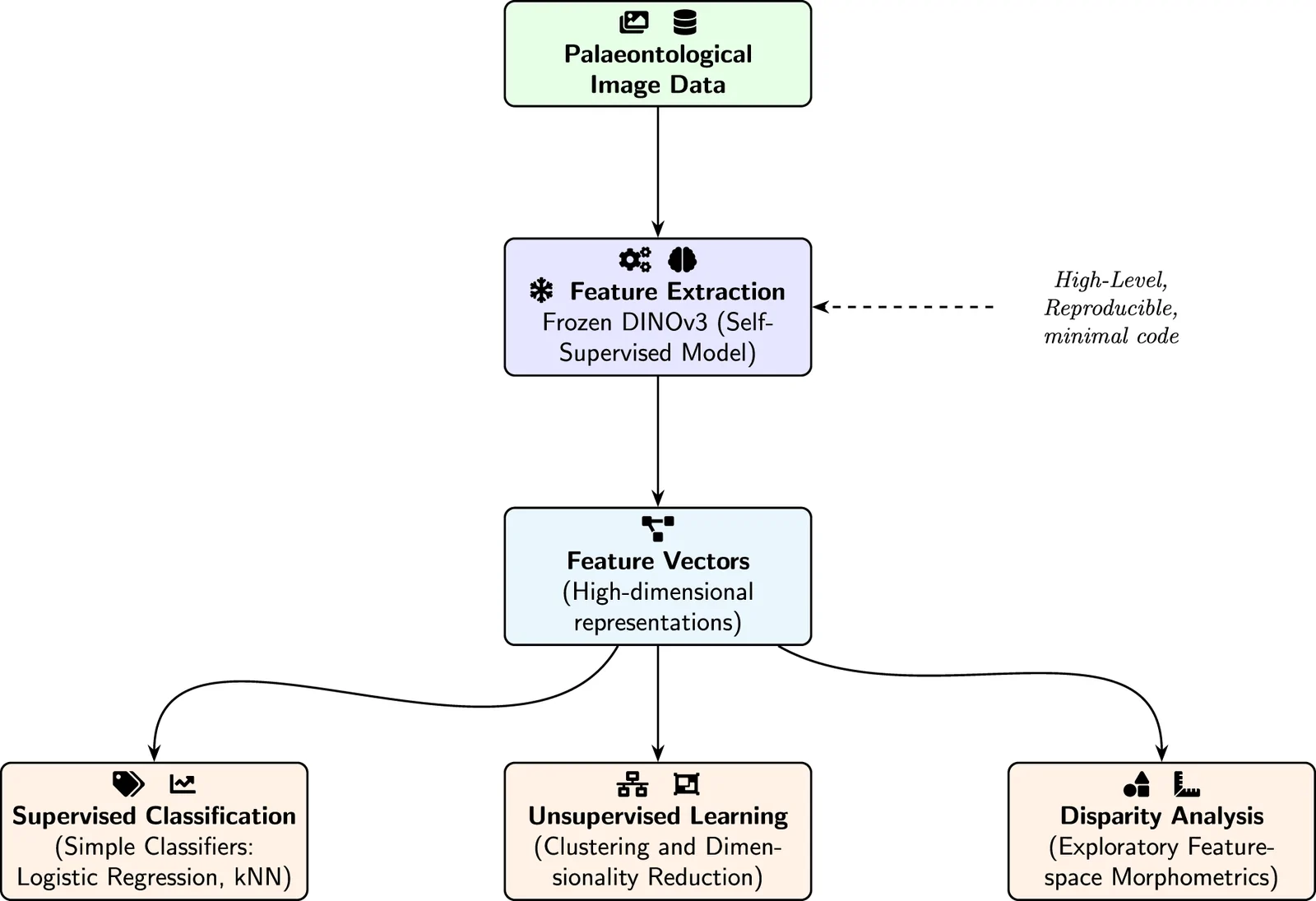
High-level overview of the pipeline proposed in this study.

## 2. Literature review

### 2.1. Machine learning in palaeontology

ML has been widely used in palaeontology, see reviews [1–3]. Thus far computer vision in palaeontology has been dominated by CNNs using transfer learning [1–3]. However, some studies have begun to use more recent frameworks such as: the application of metric learning to foraminifera [16], vision transformers to radiolaria [17] and the application of generative adversarial networks to pollen [18].

Despite the success of CNN-based approaches, there are still limitations. A significant amount of labelled training data are still required, even for transfer learning approaches, and ideally GPU access to fine-tune the model. There are many choices that must be made and hyperparameters selected when building the CNN pipeline, such as the base model to use [3] and the data augmentation methods to employ, which can affect the performance of the pipeline [19].

Therefore, there still exists a barrier-to-entry in applying ML to novel palaeontological images as enough data need to be gathered to verify it works and the researcher has to invest time in fine-tuning the model. Recent developments such as foundation models based on SSL, which offer to partially resolve some of the above limitations, have yet to be applied to palaeontological data.

### 2.2. Self-supervised learning and the self-distillation with no labels models

SSL is a methodology used to construct representations of unlabelled data such that they can be useful for downstream tasks [7,8], which has reshaped the modern ML landscape. SSL has been a key component of the rapid advances in natural language processing beginning with models like BERT [20] and leading to the GPT family [21], allowing models to be trained on the vast amount of unlabelled data [7,8].

The self-**di**stillation with **no** labels (DINO) self-supervised approach to training vision transformers for feature extraction without labels was first proposed in 2021 [5]. This was then iterated upon [4,6,22]. In this work, we focus on DINOv3 [4], the state-of-the-art variant at the time of writing, trained on billions of images. The DINO training framework can compete with (or exceed) the results of supervised CNNs in image classification tasks [4–6] using only a simple linear or kNN classifier applied to the features. Additional description of DINO models is given in the supplementary material.

At the time of writing, DINOv3 [4] has been released too recently to have been widely applied in the academic literature, although initial results on medical images [23], anomaly detection [24] and AI-generated image detection show promise [25]. However, previous iterations of the DINO family have been applied in a diverse range of contexts. Examples of applications where DINO features are used as part of the pipeline include but are not limited to: geological rock images [26], identification of plant species [27], individual animals [28], geophysical data [29], industrial anomaly detection [30], robotics [31] and medical imaging [32,33].

## 3. Feature extraction

The promise of this methodology is to make the conversion of images into rich features as simple as possible. The cornerstone of this is the Transformers Python library produced by the company Hugging Face [9]. This library allows for the easy sharing of models, providing easy access to state-of-the-art ML. In addition, it is very well documented; see example code and detailed description of the model used in this work at http://huggingface.co/facebook/dinov3-vitl16-pretrain-lvd1689m. The library [9] allows for the automatic selection of the appropriate preprocessor for a given model (details given in supplementary material). This allows us to directly pass images to the model irrespective of the image format or dimension.

From the DINOv3 model family [4], we select the large vision transformer trained on web data with approximately 0.3 billion parameters, distilled from the full DINOv3 model. The selected model converts images to 1024-dimensional feature vectors. The size of this model balances inference speed and embedding quality; smaller models could be used in settings where computational resources are more restricted, and larger models could be used where gains in accuracy are critical and resources are less constrained.

## 4. Datasets

To test the proposed pipeline, we selected five datasets relevant to various parts of palaeontology. Our criteria for dataset selection are that the data (or a representative subset) are freely accessible from a public repository. The datasets should also have been used for classification such that a headline accuracy score, the primary currency of papers in the area, is generated which can be reasonably compared to the robust cross-validated pipeline we present. There are many difficulties surrounding simultaneous exact reproductions of multiple ML pipelines in palaeontology, something also discussed in [3]. These include differences in data splitting and cross-validation, discrepancies between reported number of images and number downloaded and variations in approaches to imbalanced data and accessibility of test sets. As a result of this, we choose to compare our pipeline to the headline numbers presented in the literature rather than an exact reproduction of every step of every paper. Results should be caveated by this fact. However, our cross-validated pipeline is potentially more representative than a single test set. This is justified as although the results show that we can match the headline accuracy, even if this was not the case, the methodology would still be valuable based on the data efficiency, accessibility and unsupervised learning potential. Thus, we encourage researchers to implement this approach and test on their own data. In addition, this is required for brevity and accessibility. This is one of the few works to simultaneously tackle ML across different areas of palaeontology using a single pipeline.

### 4.1. Pollen

Pollen grains are important for our understanding of Earth's history and generally preserve well. In order to test the ability of this pipeline to classify pollen grains, we select the dataset used in [14]. This contains pollen grains from 46 classes (mainly species with some family classes spread over 37 families), from the New Zealand and Pacific region. The roughly 19 500 images are collapsed ‘z-stacks’ of microscope images taken at different focal planes, artificially coloured to show depth.

The classification of this type of pollen can be done exceptionally well by automated means with [14] achieving 97.86% validation accuracy, in their cross-validated pipeline. Ideally, the CNN pipeline would also contain a withheld test set to prevent data leakage during early stopping or any hyperparameter tuning; however, data and computational requirements can restrict this. By contrast, as DINO features and simple classifier do not require a validation set, this reduces the opportunities for data leakage.

### 4.2. Radiolaria

To test the methodology on a smaller dataset with a number of classes amenable to interpretation for unsupervised classification, we use the dataset of radiolaria assembled in [12]. The work of Carlsson *et al.* [12] splits the dataset into three sets, with one set labelled ‘S’ being the full data and the others the same data except for the removal of broken or blurred objects. This full dataset we use contains 1085 microscope images of radiolaria across eight species from the genus *Podocyrtis*. On the full dataset the CNN-based method of Carlsson *et al.* [12] achieved a validation accuracy of 88.46%.

### 4.3. Foraminifera

Foraminifera are a group of organisms to which ML has been applied in palaeontology, such as in the creation of the Endless Forams dataset in [10]. ML is also appealing owing to the possibility of pairing ML with automated systems to physically manipulate objects [34]. Large numbers of samples have allowed the implementations of data-intensive methodologies, such as [35] which uses vision transformers and generative adversarial networks to generate synthetic data. The Endless Forams dataset and subsets of it have been used to test automated classification of foraminifera several times [10,11,16]. The total Endless Forams dataset contains approximately 34 000 images which are available online [10]. However, they are not downloadable as a single folder, so we take the subset of this data. This was approximately 27 000 images split into 35 species classes, uploaded to Zenodo [36] as part of [11].

Using the complete dataset and a standard CNN, Hsiang *et al.* [10] achieved a maximum accuracy of 87.4% on a cross-validated pipeline. More specialist architectures which are trained from scratch on the approximately 27 000 image dataset (while removing small classes) managed to achieve a maximum accuracy of 90.3% [11], at the cost of increasing training time. Using higher-resolution images and metric learning on the approximately 34 000 image dataset, Karaderi *et al.* [16] managed to reach 92% accuracy, again this is at the cost of increasing training time, requiring 48 hours on a GPU [16]. The discrepancies in image numbers, how small classes are dealt with and evaluations between papers highlight the difficulties in reproduction of results in ML in palaeontology.

### 4.4. Fossil tracks

In order to test the ability of the model to work with ambiguous objects that are simple outlines without colour or textural information, we select the dataset of Lallensack *et al.* [13]. This dataset consists of 693 ornithischian tracks and 980 theropod tracks which are simple binary images consisting of the track itself and no background. In [13], the dataset is downsampled to create a balanced dataset and then tested on a small fixed set of 36 images so that the test set can be passed to human experts. As we cannot directly access the split of the downsample or the test set, we train on the full dataset.

In [13], human experts and the neural network achieve similar accuracy scores as some of the examples are fundamentally ambiguous. The work of Lallensack *et al.* [13] presents results with a binary classifier or with a third unsure option; however, for consistency, we focus on the binary case where their methodology achieves 86% accuracy on the fixed test set after a validation accuracy of 83% during the training process.

### 4.5. Diverse fossil images (reduced fossil image dataset)

Finally, to test our methodology on diverse data, we test our method on the images that were web-scraped in [15]. This large dataset consists of 50 major fossil groupings covering invertebrates, vertebrates, plants and microfossils. As to be anticipated from data which are scraped from the internet, there are a large variations in image quality (and relevance), background and image type (isolated skeletal subsections, whole specimens or sketches).

We select the reduced but still large version of the dataset of approximately 60 000 images as this is more than enough data for our methodology to be effective. This reduced fossil image dataset (RFID) has been downsampled to remove the imbalance present in the full dataset with each class containing approximately 1200 images. The authors of [15] manage to achieve a test top-1 accuracy of 82.97% on this dataset and also reported a top-3 accuracy of 92.57%.

## 5. Supervised classification

### 5.1. Classification pipeline

The goal of our classification pipeline is accurate and robust results while maximizing accessibility and simplicity. Hence, we directly pass the feature vectors to two simple classifiers, logistic regression and kNNs from [37].

A kNN classifier simply takes an unclassified data point and finds the kNNs, according to the given distance metric, then classifies the unknown point by taking the majority class of the kNNs. Logistic regression (or multinomial logistic regression in the case of more than two classes) uses linear boundaries in feature space. This is achieved by applying a sigmoid (binary) or softmax (multi-class) function to linear combinations of the variables to convert them to probabilities of belonging to a particular class.

When using the kNN classifier, we use a cosine distance metric, k=5 neighbours and $l^{2}$ (Euclidean) normalization of the feature vector. This is standard practice for high-dimensional embeddings, as angles are more meaningful than absolute distances. When performing logistic regression, we directly use the default settings of sklearn [37] aside from fixing a random seed for reproducibility, increasing the maximum iterations to 1000 and using the balanced class weight setting. This directly handles data imbalance by weighting the importance of examples inversely to the number in that class.

We do not claim that this pipeline is optimal. Improvements could be made by doing dimension reduction, using a different classifier or by hyperparameter tuning. However, we explicitly choose the minimal simplest pipeline to demonstrate that DINOv3 features are rich and that literature benchmarks can be matched (or exceeded) with this approach. In certain situations, small gains in accuracy could be made by using a nonlinear classifier. However, DINO features generally tend towards linear separability of classes, so gains are likely to be marginal while risking over-fitting. Shortcut learning is a generic problem in ML, where the classifier learns to classify based on spurious artefacts correlated with the class labels, the possibility of this is reduced by having fewer trainable parameters and linear classification boundaries.

Another benefit of this approach is that because of the ease of training, we can adopt a rigorous repeatable cross-validated pipeline (detailed in the supplementary material), ensuring each sample is used for both testing and training, something that is not universally employed in the CNN-based palaeontology ML literature as described above.

Classification metrics are reported as the mean and standard deviation combined across folds and repeats. In the main text, we focus on accuracy to compare to the literature with tables listing precision, recall, f1 score, specificity, as well as balanced, top-2 and top-3 accuracy given in the supplementary material.

### 5.2. Matching literature benchmarks

In table 1, we compare the maximum accuracy score of our method to the headline accuracy score shown in the literature (the maximum accuracy given in the cited work). For all cases, our maximum accuracy occurs when applying logistic regression to the maximum amount of data. On the RFID, the kNN classifier is numerically very close to the logistic regression, as it is likely that the dataset clusters separately in feature space.

**Table 1.** Comparison of maximum classification pipeline accuracy to reported headline literature accuracy. Numbers should be viewed as representative given variance in evaluation protocols.

dataset	maximum accuracy [our pipeline, self-supervised] (%)	representative headline literature accuracy [CNN] (%)
pollen	**98.34**	97.86 [14]
radiolaria	**94.30**	88.46 [12]
fossil tracks	81.28	**86** (small test set) [13]
diverse fossils (RFID)	**83.31**	82.97 [15]
foraminifera	86.95	87.4 [10], 90.3 [11], **92.0** [16]

Our cross-validation pipeline exceeds the reported accuracy in pollen, radiolaria and the RFID. In pollen classification, our method is exceptionally accurate, as is the CNN approach. In radiolaria, we exceed the accuracy of the literature benchmark, although this may be owing to choices made during the CNN pipeline design. We also perform well on the RFID with top-3 accuracy of 94%. We do not exceed but are around the same accuracy on the fossil tracks small test set and are close to the reported validation accuracy of 83%. This is not unsurprising, as the fossil track dataset contains many fundamentally ambiguous cases. DINOv3 is designed to extract features from complex RGB images; that it performs almost as well as a fine-tuned CNN on simple binary shape outlines is notable. In addition, this case may be the result of variance between protocols. We perform a full cross-validation and must compare to a small curated test set so our evaluation is, in some senses, more challenging.

In foraminifera classification, our reported accuracy is very close to the accuracy of Hsiang *et al.* [10] who used transfer learning. It is below the bespoke network trained in [11] and the metric learning approach of Karaderi *et al.* [16]. This is again unsurprising, DINOv3 plus simple classifier matches the performance of transfer learning but can be beaten at the cost of coding a complex pipeline and the use of days of GPU time [16].

However, these results taken together demonstrate that given the trade-offs (or lack thereof) between accuracy and simplicity, it makes sense for DINOv3 plus a simpler classifier to replace CNN-based transfer learning, at least in the first instance. Then, only in cases where accuracy is paramount and data volume allows, should bespoke methods be considered. Cross-validated confusion matrices built using logistic regression applied to all available data can be found in the supplementary material. Of course, differences in evaluation protocols mean that this should be viewed as a representative comparison between methodologies; however, this still shows that established trends in ML transfer to palaeontology as would be expected.

### 5.3. Reducing dataset size

Another advantage of our pipeline is data efficiency. Across all datasets tested, performance is reasonably good at only 10% of the data and the rate of increase begins to plateau at only 30% at the data used (figure 2). Results are competitive with the literature headline accuracies with only 30% of the data when classifying pollen, radiolaria and the diverse images. However, the rate of increase is slower in the case of foraminifera and the fossil tracks. Data efficiency is a key asset for palaeontology and may allow a wider array of questions to be studied.

**Figure 2.**
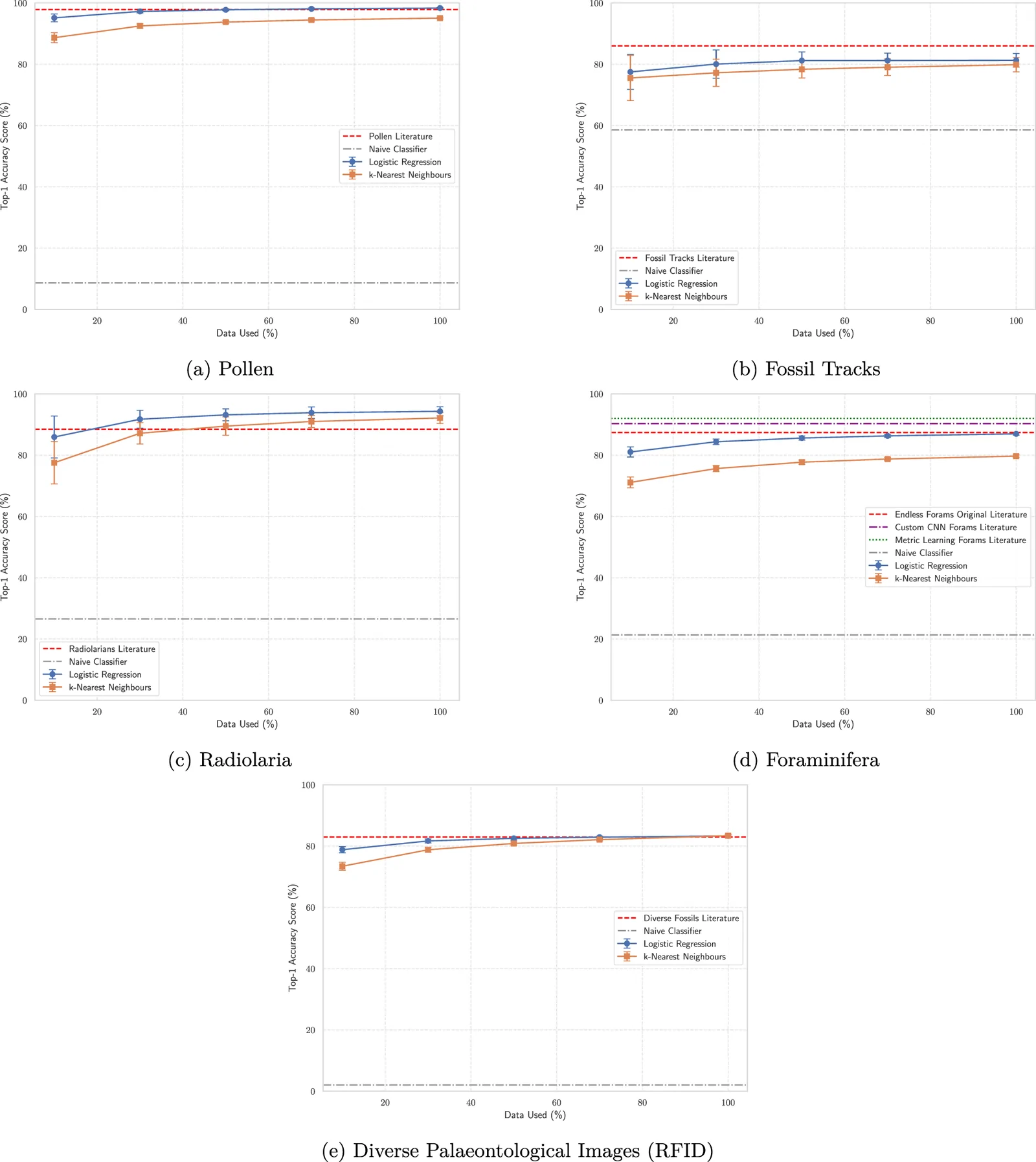
Changes in mean accuracy of our pipeline with fraction of data sampled for each dataset, panels (a)–(e). Headline literature accuracy and a naive classifier accuracy (always predicting the most common class) are shown as horizontal lines. Error bars are standard deviations over folds and repeats. Comparison to literature methodologies is representative, given different evaluation protocols and dataset sizes.

### 5.4. Few-shot learning

Few-shot learning is the process whereby an ML algorithm learns from a small number of examples. Gaining insight from limited data is useful in both proof-of-concept tests and situations of data scarcity.

For few-shot learning, we use a similar classifier set-up as in the previous section with the logistic regression unchanged, while in the kNN approach, we only change that number of neighbours is now set as either five or the maximum number of allowed training examples per few-shot run, whichever is smaller. We randomly select n training examples per class from the set [1,2,3,5,10,20]. If n is higher than the number of examples in a class minus one, we select up to this point to leave at least one example in the test set. We then test on the remaining data and repeat this process 50 times. All metrics, for a given number of training examples, are given in terms of mean and standard deviation over these repeats. In the main text, we again focus on the accuracy. Full metrics are given in the supplementary material.

Few-shot learning results are promising (figure 3). Something that would not be possible using a CNN-based pipeline, as it could not be fine-tuned using only a handful of examples. Pollen, radiolaria and the RFID are classified much more accurately than the naive classifier, selecting the most common class, after only a single training image. Classification performance steadily increases towards the literature headline accuracy with the maximum number of examples. The fossil track images, which are both simple and ambiguous, take longer to see improvements above the naive classifier; however, this is unsurprising, given this is an imbalanced binary classification problem. The foraminifera classifier shows slower growth with number of examples but again a reasonably accurate classifier can be achieved with only a small number of training examples. For both foraminifera and fossil tracks, it is likely that more examples are needed to learn the nuanced differences between classes. The tasks that performed best when the data volume was reduced (figure 2) also perform better for a smaller number of few-shot examples, showing that these are more easily distinguished in feature space. Few-shot learning performance suggests that the features extracted are robust and that shortcut learning is less likely to explain the performance.

**Figure 3.**
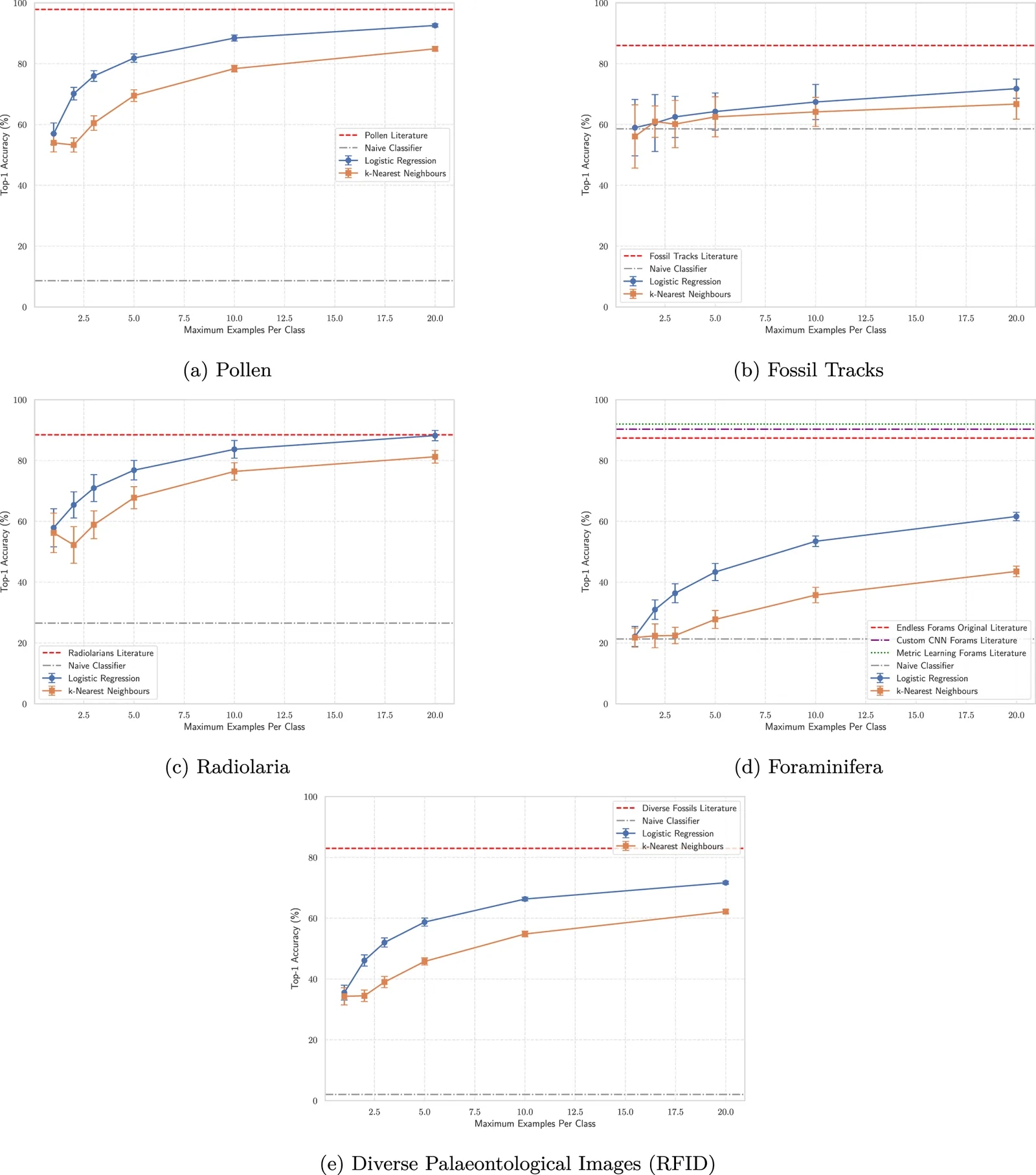
Changes in mean accuracy when doing few-shot learning for a given maximum number of examples per class for each dataset, panels (a)–(e). Headline literature accuracy and a naive classifier accuracy (always predicting the most common class) are shown as horizontal lines. Error bars are standard deviation over repeats. Comparison to literature benchmarks are representative given different evaluation protocols and dataset sizes.

## 6. Unsupervised learning

Unsupervised learning is the process of extracting information and groupings from unlabelled data. In this section, we demonstrate how the features generated by DINOv3 can be used to find groupings and structure in data without labels. Unsupervised feature vector clustering has previously been used to group by morphology, such as in the work of Couret *et al.* [38] on mosquito classification. Firstly, we show how dimension reduction can be used to visualize the features and how the visualized structures align with the true labels. Then we apply unsupervised clustering methods to automate the detection of groupings in the data. As seen in figures 4 and 5, when there is a large number of classes and points, visualization of results is difficult. Hence, we give large images where labels can be read more clearly in the supplementary material and the code repository. However, we note that when initially viewing the images the emphasis here should be on the qualitative emergence of structure. In practice, if doing a bespoke analysis of a single dataset, one would use an interactive figure which allows zooming and mouse over display of sample names to understand the distribution of points. An important caveat surrounding unsupervised learning is that it may detect all features which lead to groupings, including potentially spurious features unrelated to biology.

**Figure 4.**
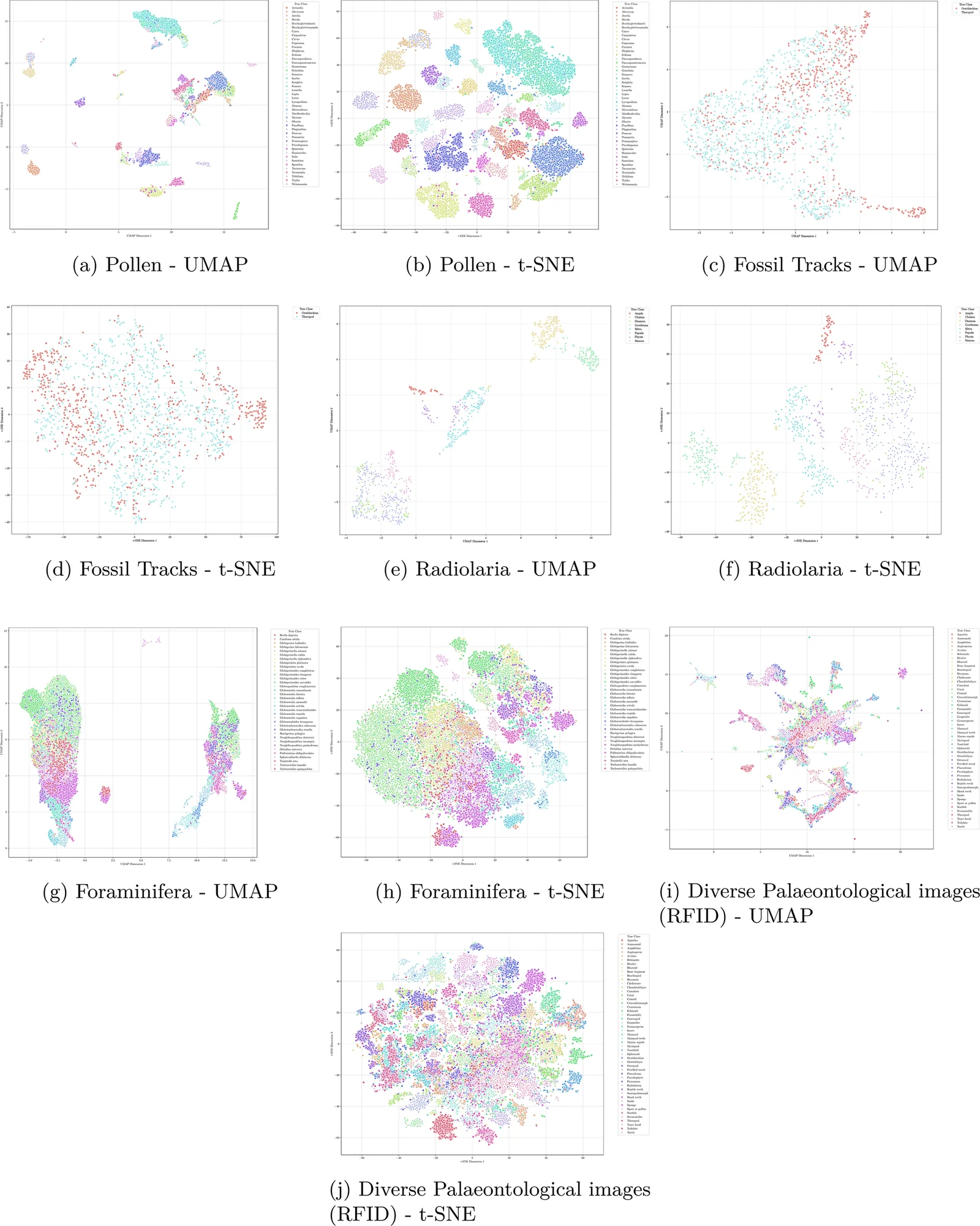
Nonlinear dimensionality reduction methods applied to feature vectors for all images across all datasets (panels (a)–(j)), points labelled according to their true class. Larger version of images and colour label correspondence given in the supplementary material, with high-resolution images in the code repository. Emphasis is on the qualitative emergence of structure matching label assignments; detailed correspondence can be viewed in the supplementary material.

**Figure 5.**
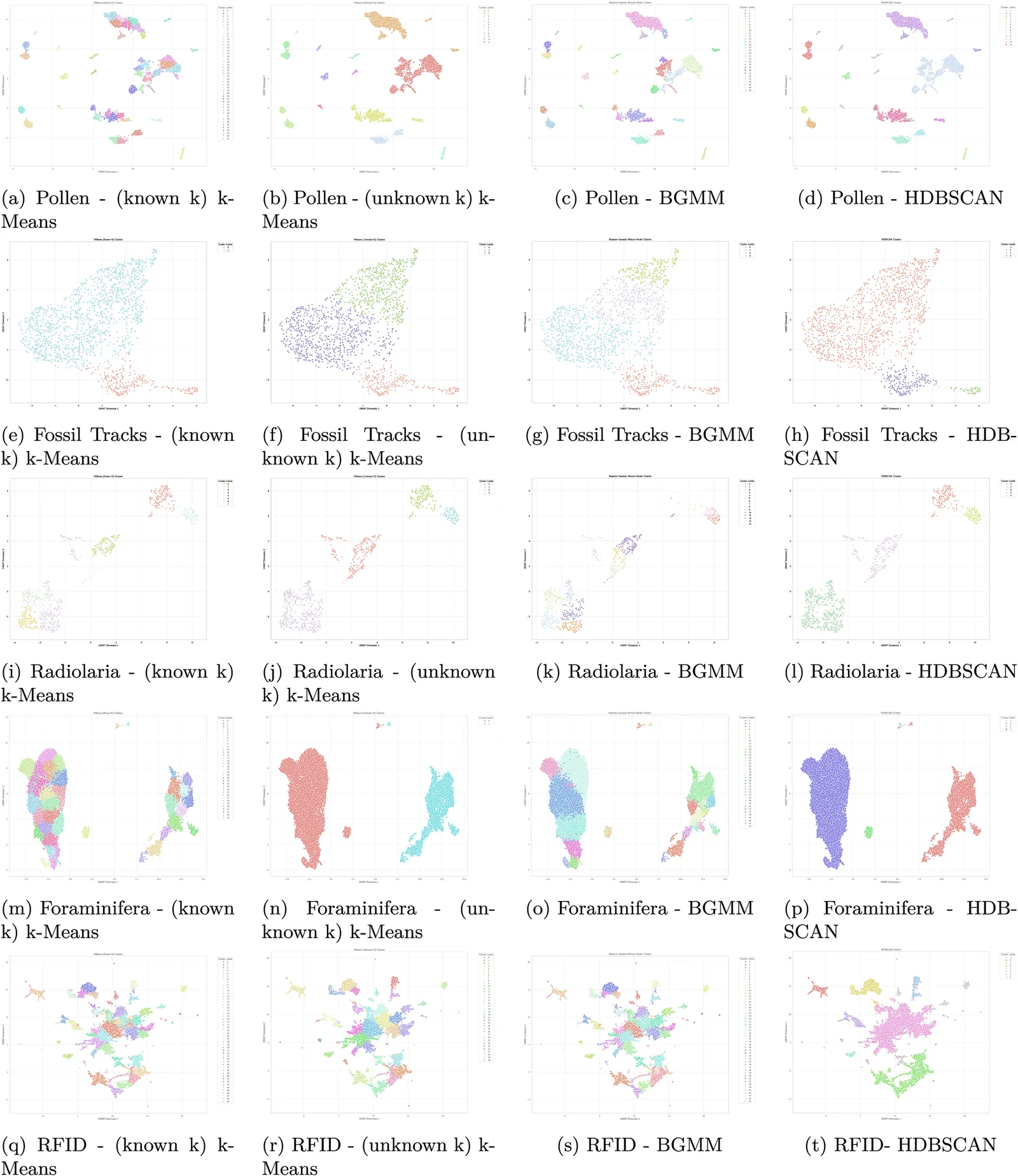
Comparison of exploratory unsupervised clustering across all datasets and methods, panels (a)–(t). Larger version of images and colour label correspondence given in the supplementary material, with original high-resolution images in the code repository. Emphasis is on the qualitative emergence of structure and variety of clustering patterns.

### 6.1. Visualization of feature vectors

As the features generated by self-supervised models are high-dimensional objects, for visualization purposes, we reduce them to two dimensions. This can be done with linear methods such as principal component analysis (PCA) or by using nonlinear methods like uniform manifold approximation and projection (UMAP) [39,40] or t-distributed stochastic neighbour embedding (t-SNE) [41] (accessed through [37]). Note that dimension reduction is a lossy procedure, and the feature picked out by the dimension reduction scheme may not be the biologically relevant ones.

Unfortunately, see supplementary material, PCA struggles to adequately represent these feature vectors in a small number of dimensions owing to the large number of classes and how the model represents the data. As the transformer architecture leads to an embedding space with an anisotropic nonlinear structure, it is not well suited to linear dimension reduction. [4,6].

As a result, we focus on nonlinear dimensionality reduction methods; however, this reduces interpretability and accessibility. Interpretability decreases as distances are not preserved when doing nonlinear dimension reduction. Accessibility is reduced as these methods are less common in palaeontology compared to PCA; t-SNE [41] is a nonlinear method for visualizing high-dimensional data which converts data into a lower-dimensional space while trying to preserve the pairwise similarities between points; UMAP is another method for nonlinear dimension reduction [39,40]. UMAP attempts to find a low-dimensional representation of the data that preserves as much as possible the topological structure of the high-dimensional data [40]. UMAP is more computationally efficient than t-SNE and can preserve more global structure. Parameters for nonlinear dimension reduction methods are given in the supplementary material.

Figure 4 shows the application of t-SNE and UMAP to each set of features derived from the data used. In general, figure 4 demonstrates that DINOv3 features contain biologically meaningful information, as classes generally plot close to each other, in relatively well-defined groupings. This plot demonstrates why some of our datasets are easier to classify than others. Some datasets such as radiolaria, pollen and to an extent the RFID break into discrete groupings organized into classes. Whereas, others like the foraminifera and, in particular, the fossil tracks do not break into distinct groups in the same way. In addition, when viewing the UMAP of the radiolaria (figure 4e), we can see three distinct groups that may relate to the three evolutionary lineages in the data [12].

When working with feature vectors, non-taxonomic groupings may appear owing to patterns in the image data unrelated to biology. The fossil tracks dataset embeds into a single group highlighting the ambiguous region where it is difficult to categorize images and the model struggles to pick out the nuance that a specialist may. In addition, the foraminifera groups into two large macroscopic clusters as a result of the fact that the dataset contains images of two distinct backgrounds and colour grades, so the feature vectors group objects of similar species independently in each of these two clusters. This may explain why we required more examples to classify the data well in §5. This short demonstration (figure 4) highlights the utility of the dimensionality reduction as a data visualization and inspection tool. However, it should also act as a cautionary tale demonstrating that feature extractors can find non-biological groupings of data.

### 6.2. Clustering methodology

To further test how biologically relevant information can be extracted from feature vectors, we tested several unsupervised clustering algorithms and compared the extracted clusters with biological labels. Before clustering, we reduce the dimensionality of the data, as unsupervised clustering algorithms based on distances can be computationally costly, and distances are less meaningful in high dimensions. This is done using PCA and UMAP [39,40] on $l^{2}$ (Euclidean distance) normalized feature vectors. When using PCA to reduce dimension for clustering, we reduce dimension to the minimum of 50 and the number of components needed to explain 95% of the variance. In practice, as the features are not conducive to linear dimension reduction, all datasets are reduced to 50 dimensions. UMAP is a powerful preprocessing step for clustering, but should be treated with care and used as an exploratory method as the nonlinear reduction in dimensionality does not preserve distances between points. When using UMAP for dimensionality reduction, we set the target dimension to 15.

We test four different methods for unsupervised (or partially unsupervised clustering), giving the parameters used for each in the supplementary material.

Firstly, we use partially unsupervised clustering, k-means clustering where the number of clusters expected, k, is known in advance; k-means finds clusters and assigns points such that the distances between points and the centroids of their assigned clusters are minimized—this k-means method runs very quickly, and in some scenarios, it may be realistic that there is some knowledge of how many clusters to expect.

The second method used is k-means with an unknown number of clusters. We fit k-means for varying values of k between 2 and min(2×#classes,50). In practice, the range would be selected with an educated guess. The best value of k is selected using the silhouette score [42], via scikit-learn [37]. This compares the mean intra-cluster distance to the mean distance to the nearest cluster. This also justifies the range selection as silhouette score selection probably tends towards selecting a smaller number of larger clusters.

The third method used is a Bayesian Gaussian mixture model (BGMM), again from Pedregosa *et al.* [37]. This method assumes that the data points in each cluster are drawn from a Gaussian distribution and automatically selects the probable number of clusters using Bayesian inference.

The final clustering method used is Hierarchical Density-Based Spatial Clustering of Applications with Noise (HDBSCAN) [43], which performs density-based clustering across scales and aims to identify the most stable clusters. This algorithm automatically determines the number of clusters and assigns points away from dense regions as noise. Unsupervised clustering is more difficult than supervised learning as it relies on the classes being well separated in feature space according to taxonomically relevant features. In addition, it is sensitive to parameter choice and objects may cluster at different scales. In practice, this would be dealt with by repeated tests with different parameters and algorithms, trying to determine which clusters are robust and what biological features they correspond to. As this work is a proof-of-concept and we focus on demonstrating how methodology works, we run the algorithms once and plot the resulting clusters on a visualization UMAP (parameters are given in the supplementary material).

In the main text, we only show the clusters from the exploratory UMAP pipeline, as they generally cluster much better than PCA as the DINOv3 embeddings have an intrinsically nonlinear structure. In the supplementary material, we give detailed quantitative metrics for each clustering algorithm and dimensionality reduction method.

### 6.3. Clustering results

The results of clustering of the data are shown in figure 5. It is clear that different algorithms pick out different groupings at different scales; however, meaningful groupings are found when comparing the plots to those by true class (figure 4). Full discussions of the quantitative clustering metrics for each dataset are given in the supplementary material. In general, knowing the level of granularity to cluster at and which features are truly biologically informative is a difficulty shared by all unsupervised learning methods in this domain.

Clustering of the pollen data is shown in figure 5a–d. Well-defined clusters are generally found; however, algorithms that infer the number of clusters generally cluster above the level of species. The fossil tracks (figure 5e–h) do not clearly separate in feature space. Hence, the algorithms break the objects into various subgroups. These represent genuine observed morphological variation, but do not precisely align with given labels and may not align with biologically relevant features. Clustering the radiolaria data is generally successful (figure 5i–l), with clusters aligning with species or groups of similar species depending on the scale, with larger scale groupings aligning with the three evolutionary lineages in the data [12]. When clustering foraminifera (figure 5m–p), some of the algorithms focus on the differences in image backgrounds and colour scales, creating two large macroscopic clusters. In addition, other approaches like the BGMM (figure 5o) find clusters which partially overlap with the known labels. Clustering the RFID (figure 5q–t), we again find generally well-defined clusters across different scales. The results of figure 5 and supporting quantitative metrics in the supplementary material show that well-defined groupings can be extracted directly from palaeontological image data using DINOv3 when care is taken to avoid clustering image artefacts.

### 6.4. Disparity quantification by feature vector-based metrics

It is also possible to use the feature vectors for each dataset to compute various measures of exploratory feature vector-based disparity (details given in the supplementary material). There are several caveats with this approach; however, it can be shown (supplementary material) that, as expected, the RFID is much more disparate based on the mean pairwise distance, effective rank and other measures owing to the diversity of object classes in the dataset. This shows how it may be possible for feature vector statistics to be used to quantify underlying features of a dataset.

## 7. Visualizing features captured by model

A useful step to confirm that the DINOv3 model is behaving as expected and focusing on relevant image regions, and test against shortcut learning, is to visualize the intermediate output of the model. We present two ways to do this. Firstly, one can construct an attention map using the register tokens (additional learnable parts of the model) [22]. Secondly, one can take output at patch-level, reduce them to three dimensions, and convert to RGB values for visualization, as demonstrated in [4]. We give details of each approach in the supplementary material. It should be emphasized that these methods are only an indication of the behaviour of the model as they involve combining output, loss of information and obscure additional nonlinearities that are applied before the final output.

When applying the feature visualization methods to a selection of images from the datasets (figure 6), we find that results are positive. In the attention maps, attention is concentrated on the objects of interest. Furthermore, for the RGB PCA, the objects of interest are distinct and plotted in a different colour from the background. Even for the complex museum image (figure 6e,f), the attention map focuses directly on the object of interest while the PCA segments out the fossil. The results of figure 6 support our observation that these models perform well in the classification of palaeontological data and provide a methodology to interpret, as far as possible, the model performance.

**Figure 6.**
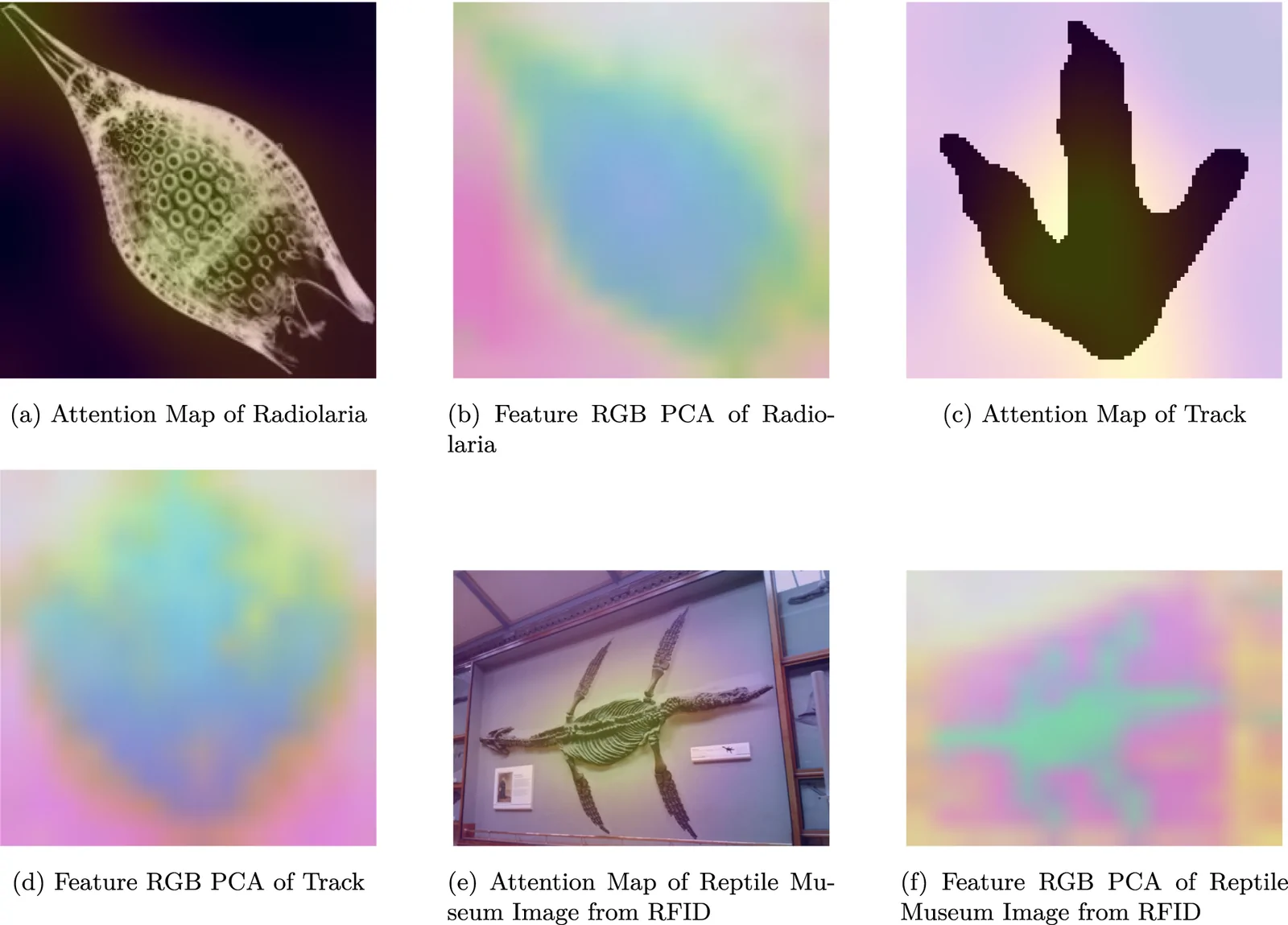
Comparison of techniques for visualizing model understanding applied to a sample of images including radiolaria *Podocyrtis Papalis* from [12] (panels (a), (b)), a described theropod footprint from [13] (panels (c), (d)) and an image from the marine reptile category in [15] (panels (e),(f)).

## 8. Discussion

The choice of using a given ML framework is not a simple analysis of the efficacy of the method in classification. It is a series of trade-offs between efficacy, accessibility, robustness and computational costs. The combined analysis of these factors demonstrates the utility of our pipeline which compares favourably with a CNN-based approach. Even if our methodology consistently underperformed previous methods, we would argue that it would still be worth adopting, given the fact that it can be used without fine-tuning, using a simple classifier, in a few lines of code, using standard well-documented libraries, on smaller amounts of data, with automated preparation.

This methodology addresses reproducibility issues in palaeontological ML studies [3] in three ways. Firstly, there are simply fewer parameters. Secondly, as the process of writing the code is faster, and the runtimes shorter, replication is easier. Thirdly, the large model can be downloaded and accessed directly from Hugging Face [9]. In addition, since the pipeline is model agnostic, DINOv3 [4] could be replaced with any relevant feature extractor by simply changing the Hugging Face model ID.

A particular benefit to palaeontology is the success of few-shot learning and learning on very small datasets. This could unlock new potential applications by allowing ML to be used in small data domains or by giving researchers the confidence to invest resources in data acquisition, preventing ideas being abandoned owing to result uncertainty. Feature extraction only needs to be run once, and can be done on a laptop, while training simple classifiers is not computationally costly, making the methodology widely accessible and more sustainable. Details of hardware used, along with a brief discussion of model size, speed and performance trade-offs, are given in the supplementary material. This work was carried out on a MacBook Pro, using the ViT/L variant of DINOv3 which can extract in batch approximately 50 images a second, with GPU acceleration. The ViT/S model extracts images approximately three to approximately eight times faster. On a CPU, tests on the radiolaria and tracks datasets show the large model extracts approximately six images a second and the small model extracts approximately 40 images a second. This is all fast enough for palaeontological data sizes, particularly for something that can be run once. The speed-ups of the small model come with drops in accuracy which vary with the task (supplementary material). Timings will be hardware and set-up dependent (e.g. if using bfloat16 as we do or not). In addition, this is also fast enough so that cross-validation or unsupervised learning may become the bottleneck. CNN training may be difficult without a GPU and with a GPU may take hours of GPU time and multiple runs over different experiments, depending on task and set-up. For the model sizes used in this work, the RAM requirements are well within the limits of consumer laptops. The thesis of this work is that previous methodologies can be matched with minimal cost; if in a scenario where every percentage of accuracy matters, then the full model can be dropped into the pipeline.

The fact that the off-the-shelf models are so powerful allows insight to be gained into datasets without the need for labels as shown in §6 on unsupervised learning and in §6.4. However, with the caveats that unsupervised learning can be sensitive to methodology set-up and structure of the dataset.

Unsupervised learning on robust features is likely to group images reflective of actual features of the image data; however, it is difficult to know *a priori* the cause of the separation if it is taxonomic, and of which rank, if it is morphological or if it is some aspect of image composition. In this work, we mainly focus on a demonstration pipeline, but in practice, one would run the unsupervised algorithm using multiple parameter sets and re-run it on large clusters to fully explore the structure of the dataset in collaboration with a domain expert treating the distortion of distances by the nonlinear dimension reduction methods with caution.

Despite the caveats, it is hoped that the unsupervised exploration of these datasets can potentially provide novel biological insight. Another potential unsupervised method, unexplored in this work, is using the embeddings for outlier detection. This could be used for dataset management, finding outliers caused by data contamination or labelling errors, or for the detection of novel objects.

Given the accessibility of this methodology, it may be useful in situations where ML-based classification is not the overall goal. Just as a PCA of morphological properties can be used as a figure to visualize a dataset in palaeontological papers, one can imagine using this pipeline as a visualization tool. In addition, an argument that objects are morphologically and visually distinct can be supported by showing that this classification pipeline can achieve near-perfect accuracy on the dataset.

A general concern in ML is ‘shortcut learning’ where the classification or clustering is the result of some spurious feature such as image artefacts. For supervised learning, this is an issue if there is a correlation between the spurious features and the class labels. As we use a frozen model, with broad and robust pretraining, and a simple linear classifier, which works in few-shot settings, it is less likely, albeit not impossible, for this framework to do shortcut learning than a CNN with many more trainable parameters. Care must be taken in unsupervised settings that the imaging artefacts do not lead to spurious clusters, as in the foraminifera UMAP (figure 4g), as the model is highly expressive so will exploit available structure. This serves as both a cautionary tale and a demonstration of how this method can be used to detect imaging artefacts. In an application where shortcut learning is likely and a specific concern, one may begin with the general fast pipeline and follow with a bespoke pipeline.

One proposal of this work (supplementary material) is to use the DINOv3 [4] generated feature vectors to quantify the disparity of a dataset. A similar concept was proposed in [44], where it was suggested to measure the diversity of pollen data using entropy-derived measurements based on CNN feature vectors [44]. However, further work and suitable dataset selection are needed to test if this methodology can be useful and whether correlation with biologically relevant parameters can be found, rather than image characteristics. A wide range of future work could be enabled by this methodology. Coupling this methodology to a segmentation pipeline could enable the automated processing of large datasets using robotics [34] or thin-section imaging [45]. Features could be used to track changes in morphology over time in evolution or ontogeny. Feature vector-derived distances could be compared to phylogenetic distances.

It could be examined how we can use these models to capture the true three-dimensional structure of objects by combining the feature vectors for different views appropriately. It may also be possible to concatenate the feature vectors with other metadata enabling multi-modal learning. Feature vectors could also be used to search collections for images similar to unknown objects based on similarity scores. Finally, more complex classifiers than those presented here could be used, or the model itself fine-tuned, where the need for accuracy or data merits this. In conclusion, we have shown how a simple pipeline using state-of-the-art pre-trained self-supervised models can match the performance of CNN-based methods and allow unsupervised insight into palaeontological images.

## Supplementary Material



## Data Availability

Code and access links to data are uploaded to GitHub at http://github.com/nrodgers1/Self-Supervised-Learning-for-Palaeontology and permanently archived using Zenodo [46]. Potential users of DinoV3 should consult the Meta AI license agreement. Software versions and model ID codes are listed in the supplementary material. Supplementary material is available online [47].
