# Supplementary material for: Complexity begets simplicity: self-supervised learning for palaeontological images with few or no labels
Source: J R Soc Interface. 2026 Oct 21;23(243):20251243. doi: 10.1098/rsif.2025.1243 (PMC13682873; doi:10.1098/rsif.2025.1243)
Supplement: Supplementary file 1 [file rsif.2025.1243.zip › rsif.2025.1243/rsif20251243_si_001.pdf]

Supplementary Information: Complexity Begets Simplicity: Self-Supervised Learning for Palaeontological Images with Few or No Labels

Niall Rodgers*[1]

[1]UK Centre for Astrobiology, School of Physics and Astronomy, University of Edinburgh, United Kingdom

August 11, 2026

Abstract

Supplementary Information to : Niall Rodgers, Complexity Begets Simplicity: Self-Supervised Learning for Palaeontological Images with Few or No Labels, *Journal of the Royal Society Interface*, 2026, 10.1098/rsif.2026.1243

Code and access links to data are uploaded to GitHub at https://github.com/nrodgers1/Self-Supervised-Learning-for-Palaeontology and permanently archived with a DOI on Zenodo https://zenodo.org/records/20812574.

*niall.rodgers@ed.ac.uk

S1 Supplementary Information for "Complexity Begets Simplicity: Self-Supervised Learning for Palaeontological Images with Few or No Labels" by Niall Rodgers

Contents

S1.1 Image-Based Disparity Analysis 2
S1.1.1 Methodology 2
S1.1.2 Results 4
S1.1.3 Discussion 4
S1.2 Supplementary Background 5
S1.3 The DINO Models 5
S1.4 Supplementary Methods 5
S1.4.1 Feature Extraction 5
S1.4.2 Supervised Classification Cross-Validation Pipeline 5
S1.4.3 Classification Metrics 5
S1.4.4 Confusion Matrices 6
S1.4.5 Visualisation Dimension Reduction Parameters 6
S1.4.6 Clustering Methods and Parameters 6
S1.4.7 Clustering Quantitative Metrics 7
S1.4.8 Visualising Features Captured by Model 7
S1.4.9 Hardware Used 7
S1.4.10 Software Versions and Model Id 8
S1.4.11 Model Selection and Performance 8
S1.5 Feature Visualisation with PCA 9
S1.6 Pollen Dataset 9
S1.6.1 Classification Results 9
S1.6.2 Clustering Metrics 9
S1.7 Fossil Tracks Dataset 10
S1.7.1 Classification Results 10
S1.7.2 Clustering Metrics 10
S1.8 Radiolaria 11
S1.8.1 Classification Results 11
S1.8.2 Clustering Metrics 11
S1.9 Foraminifera 11
S1.9.1 Classification Results 12
S1.9.2 Clustering Metrics 12
S1.10 Diverse Palaeontological Images Dataset (RFID) 13
S1.10.1 Classification Results - Diverse Palaeontological Images 13
S1.10.2 Clustering Metrics 13
S1.11 Larger Images from the Main Text 24
S1.11.1 Dimension Reduction with True Labels from Main Text 24
S1.11.2 Clustering Plots from Main Text 34
Supplementary References 54

In this supplementary information, we provide more detailed methods, classification and clustering metrics, plot confusion matrices for each dataset and show how PCA can be used for feature vector dimension reduction. We also describe the proposal of how the feature vector-based disparity could be quantified. At the end of the supplementary information we also repeat the dimension reduction plots given in the main text which can be used as reference for the colour label correspondence and examined in more detail and more easily zoomed in.

S1.1 Image-Based Disparity Analysis

By following this pipeline, we have access to the set of feature vectors which describe the objects. This raises the question as to whether these vectors can be used to quantify the morphological disparity of the objects they represent. This part of the work is more speculative in nature. However, this is justified for three reasons. Firstly, if you have followed the pipeline, the feature vectors are already computed and the disparity metrics are relatively simple to calculate, so there is very little cost associated with this method. Secondly, feature vector-based diversity measurements have previously been proposed for CNN feature vectors derived from studying pollen [1], where [1] used an entropy-based measure of diversity. Thirdly, this would represent a simple, fast and standardised way to capture some form of morphological disparity across a wide array of use cases.

As the primary purpose of this work is methodological and focuses on supervised learning and unsupervised clustering, we merely propose a set of metrics which can naturally be constructed from the feature vectors. If this method is employed in future work on other datasets with more metadata, it can be tested whether the proposed metrics and biologically informative variables correlate. The large caveat with this method is that care needs to be taken to ensure that the origin of the difference between vectors is due to the morphology of the objects and not due to differences in the background or imaging conditions, something that may need to be tested in detail on other datasets, as this is outside the scope of this work. The extracted feature vectors may have some complex geometric or anisotropic structure which further complicates the summarisation of some datasets with the proposed metrics. However, again these vectors are easy to generate so even as an exploratory tool this may prove useful.

S1.1.1 Methodology

A wide array of methods can be constructed to measure the spread, concentration and variation within a set of vectors. Hence, we propose four simple metrics, which are relatively simple to compute, relate to known concepts in palaeontology and are interpretable. The independence of these metrics depends on the choice of normalisation and distance metrics. When using euclidean normalisation and cosine similarities some metrics may give identical (or deterministically related) values due to the mathematical interactions of the definitions, norms and distances. However, we keep all metrics as they prompt different conceptualisation.

The first metric we propose is the mean and standard deviation of the pairwise distances, measured using a cosine distance metric. This metric reflects the overall morphological disparity by measuring how far apart objects are from each other in feature space. The next proposed metric is the mean and standard deviations of the cosine distance from all samples to the centroid, the l^2 normalised mean sample. This measures the spread of the objects and how much they deviate from the average object and spread across morphospace. In all metrics and normalisations the standard deviations of these quantities provide independent information, however when using l^2 normalised vectors and a cosine metric there is a deterministic relationship between the means (derived below).

The next proposed metric is the variance sum, which is defined as

$$\text{Variance Sum} = \frac{1}{n-1}\sum_{j=1}^{N_f}\sum_{i=1}^{n}(x_{ij} - \overline{x}_j)^2, \tag{S1}$$

where n is the number of samples, N_f is the number of features (1024 in our case) and $\overline{x}_j$ is the mean value of a given feature vector entry. This measures how much the feature vector set spreads out over morphospace. Depending on the choice of metric and normalisation, the value of this, may be identical or closely related to the mean pairwise distance. As we use a cosine distance metric and l^2 normalised feature vectors, our measured mean pairwise distance is numerically identical to our variance sum. This occurs as for two l^2 normalised vectors

$$\|x_i - x_k\|^2 = \|x_i\|^2 + \|x_k\|^2 - 2(x_i \cdot x_k) = 2(1 - x_i \cdot x_k) = 2d_{\cos}(x_i, x_k). \tag{S2}$$

Hence the mean pairwise cosine distance can be written as

$$D_{\text{mean}} = \frac{1}{n(n-1)} \sum_{i=1}^{n} \sum_{k=1}^{n} d_{\cos}(x_i, x_k) = \frac{1}{2n(n-1)} \sum_{i=1}^{n} \sum_{k=1}^{n} \|x_i - x_k\|^2 \tag{S3}$$

which can be written as

$$D_{\text{mean}} = \frac{1}{2n(n-1)} 2n \sum_{i=1}^{n} \|x_i - \overline{x}\|^2, \tag{S4}$$

where $\overline{x}$ is the arithmetic mean vector and elementary algebra has been used to rearrange the summations. Using the properties of averages over pairwise distances this expression is equivalent to the variance sum when the orders of summation are swapped and the sum over feature vectors is absorbed into the squared distance. However, we include both measures as they are not identical in all regimes, are both useful conceptually and simple to compute.

This result also allows us to quickly see the relationship between the first two metrics. Using the fact

$$D_{\text{mean}} = \frac{1}{(n-1)} \sum_{i=1}^{n} \|x_i - \overline{x}\|^2 = \frac{1}{(n-1)} \sum_{i=1}^{n} \left(\|x_i\|^2 + \|\overline{x}\|^2 - 2x_i \cdot \overline{x}\right) = \frac{n}{(n-1)}(1 - \|\overline{x}\|^2), \tag{S5}$$

using the properties of sums over vectors. In this specific norm and metric,

$$D_{\text{cent}} = \frac{1}{n} \sum_{i=1}^{n} d_{\cos}(x_i, \frac{\overline{x}}{\|\overline{x}\|}) = \frac{1}{n} \sum_{i=1}^{n} (1 - x_i \cdot \frac{\overline{x}}{\|\overline{x}\|}) = 1 - \|\overline{x}\| \tag{S6}$$

which can be substituted into the line above to give

$$D_{\text{mean}} = \frac{n}{(n-1)}(1 - (1 - D_{\text{cent}})^2) \tag{S7}$$

where it follows via elementary algebra that

$$D_{\text{mean}} = \frac{n}{(n-1)}(2D_{\text{cent}} - D_{\text{cent}}^2). \tag{S8}$$

The reasoning for the interdependence between metrics is due to the geometric properties which come from imposing the above normalisation which forces the vectors to point to the surface of a unit hypersphere and using a distance metric which depends on the the angle between them.

The final metric proposed is the effective rank which is a relatively easy to compute information-theoretic measure of the effective dimensionality of the morphospace. The effective rank depends on the eigenvalues, λ_i of the covariance matrix, estimated using the Ledoit–Wolf method to ensure stability for small sample numbers. The effective rank is defined as,

$$\text{Effective Rank} = \exp\left[-\sum_{i=1}^{N_f} p_i \ln p_i\right] \tag{S9}$$

where

$$p_i = \frac{\lambda_i}{\sum_{j=1}^{N_f} \lambda_j}, \tag{S10}$$

are the normalised eigenvalues of the covariance matrix. The effective rank is the exponentiation of the Shannon entropy of the normalised eigenvalue spectrum of the covariance matrix. When the probability density is concentrated in a single value ($p_1 = 1, \forall i \neq 1, p_i = 0$) the effective rank is 1. When the probability is uniformly distributed ($\forall i, p_i = \frac{1}{N_f}$) the effective rank is equal to N_f. Hence, demonstrating why it acts like an effective dimensionality and gives an indication of how filled the morphospace is. However, care should be taken when interpreting the effective rank for mixed datasets as between-group differences can lead to large eigenvalues which decrease the effective rank even though the disparity between samples has increased.

S1.1.2 Results

The first way we can analyse the feature vector-based disparity is by inspecting the histograms of the pairwise distances between objects, figure S1. This shows that datasets which contain similar objects, such as the pollen, foraminifera and radiolaria, have generally smaller distances reflecting the fact that the vectors likely point to similar regions of feature space. For the RFID, the average distance is larger and nearer to one, reflecting the fact that the objects are diverse and there is much more variety in the direction of vectors.

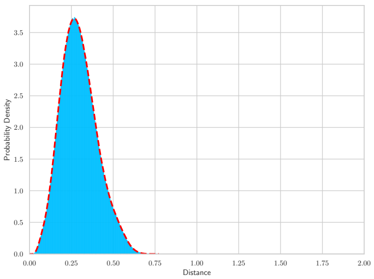

(a) Pollen

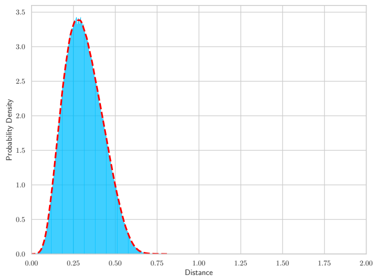

(b) Fossil Tracks

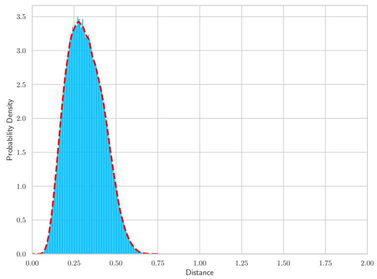

(c) Radiolarians

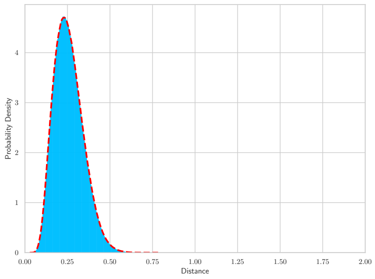

(d) Forams

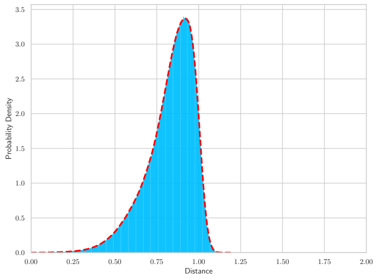

(e) RFID

Figure S1: Histograms of Pairwise Distances (Cosine Distance between feature vectors)

The proposed disparity measurements are computed for the datasets in table S1. Table S1, shows that as expected, on all counts, the diverse palaeontological images are much more disparate than the other datasets. It also shows that differences between other datasets are generally very subtle. Based on this, we would advise these metrics to be used with care, and only in situations where the morphological disparity is either directly comparable or the differences are large enough that the comparison is unambiguous. For example, it is quite fair to say that the diverse set of web images is more disparate than a dataset of foraminifera or to compare two datasets of foraminifera with different distributions of species. However, it is ambiguous and potentially inadvisable to place stock in the fact that the dataset of radiolaria has a different disparity than a dataset of pollen based on minor differences. The results of table S1, show that the proposed metrics measure different aspects of the data as the ordering of the datasets is not preserved between metrics. The derived relationship between the metrics also holds in the measured quantities.

Table S1: Proposed Morphological Disparity Metrics Applied to Datasets used in this work.

Dataset	No. of Samples	Mean Pairwise Dist.	std Pairwise Dist.	Mean Centroid Dist.	std Centroid Dist.	Variance Sum	Effective Rank
Pollen	19667	0.2965	0.1090	0.1613	0.0608	0.2965	42.0603
Fossil Tracks	1671	0.3110	0.1116	0.1699	0.0661	0.3110	48.3432
Radiolarians	1085	0.3154	0.1061	0.1724	0.0563	0.3154	54.7914
Endless Forams Original	27729	0.2578	0.0839	0.1385	0.0547	0.2578	76.3005
Diverse Fossils (RFID)	59244	0.8301	0.1408	0.5878	0.1563	0.8301	197.3126

S1.1.3 Discussion

The methods we proposed to explore disparity, do not claim to be exhaustive or a uniquely optimal choice as given a set of vectors there is a whole host of statistics and measures that could be defined. However, our proposal has merit given the readily accessible nature of the feature vectors and the relative simplicity

of the calculations. Given this, it is an almost cost-free addition to a suite of measurements if the utility can be proven. The caveats with this approach are that metrics based on feature vector distances are not intuitively interpretable, and separating effects of shape, texture, pose, colour and other image artifacts may be difficult in practice. However, there may be situations where a fast way to quantify the disparity between large unlabelled assemblages is required, and this provides a solution to this use case.

S1.2 Supplementary Background

S1.3 The DINO Models

The DINO models are freely available (subject to the META AI custom licensing agreement) and were built by Meta AI, leveraging their access to large amounts of data and computational resources.

All DINO models build on the same core concept, the coupled training of a student and teacher network [2]. Later variants added the ability to scale to larger datasets and other modifications to improve the stability of training and embedding quality [3, 4, 5]. In order to achieve the goal of creating rich feature vectors, the training procedure is as follows: a global view of an image is passed to the teacher network, which produces an output vector; different augmented views of the same image are passed to the student network, which produces output vectors; a loss function is computed which measures the discrepancy in the output vectors between the teacher and student; parameters are updated to minimise this loss function and the teacher network is updated using an exponentially moving average of the student network. This training process coupled with large amounts of data is what allows the models to produce useful feature vectors even on unseen complex objects.

DINOv3 was trained in two configurations by MetaAI, one using satellite image data and another using web data [5]. We focus on the web data variant as this is more appropriate for our datasets. To begin training DINOv3 roughly 17 billion images were collected from Instagram. This dataset was then subsampled to create a balanced dataset of approximately 1.7 billion images used to train the model [5]. The complete DINOv3 model has around 7 billion parameters, so to reduce computational burden the model was distilled into a range of smaller variants, which approximate its behaviour [5].

S1.4 Supplementary Methods

S1.4.1 Feature Extraction

The model can be automatically initiated using the Transformers library [6] and the model ID. Once loaded it can be applied to the preprocessed images and the output features collated. The automatically selected preprocessor performs the following. The selected preprocessor converts the image to an RGB PyTorch tensor of size 224×224 rescaled so that pixels take values between 0 and 1 and normalised using the ImageNet mean $[0.485, 0.456, 0.406]$ and standard deviation $[0.229, 0.224, 0.225]$.

S1.4.2 Supervised Classification Cross-Validation Pipeline

When performing supervised learning we use the following cross-validation pipeline. We take the complete dataset (defined as all the data to which we have access) and sample the following fractions $[0.1, 0.3, 0.5, 0.7, 1]$, repeating this process 10 times for each fraction. For each sample, our aim is to perform 5-fold cross-validation. For small dataset fractions, this may prove impractical. If the minimum number of examples per class is < 5, then we simply perform an $80 : 20$ train-test split, attempting stratification if possible, and repeat 5 times to approximate multiple folds.

S1.4.3 Classification Metrics

Accuracy alone is not enough to fully understand the behaviour of a classifier; however, it does give a good overall impression of how a classifier performs. As accuracy is the main metric used to illustrate performance in the literature, we focus on this for our literature comparison, but for completeness list other relevant metrics in this supplementary information. The additional metrics used are described below. Firstly, we measure top-2 and top-3 accuracy which measure the fraction of predictions where the true labels are in the top-2 or top-3 predictions, respectively. This is useful when working with a large number of classes as it gives a sense if the errors are between similar classes and if the classifier has learned the structure of the data or if the predictions are more random. We also measure the balanced accuracy, which re-weights the accuracy

to account for class imbalance. We also measure the standard metrics of precision, recall, specificity and F1 scores, for each of these metrics we provide the 'macro' version from scikit-learn [7] which does not account for class imbalance aside from the F1 score where we also measure the 'weighted' version which does. The precision is the ratio of the True Positives to the sum of True Positives and False Positives, averaged across classes which gives the fraction of positive classifications which are correct. The recall score is the ratio of the True Positives to the sum of the True Positives and False Negatives, which is the fraction of positives identified. The specificity is the ratio of the True Negatives over the sum of the True Negatives and False Positives, which gives the fraction of negative classifications identified. While, the F1 score is the harmonic mean of the precision and recall.

S1.4.4 Confusion Matrices

Confusion matrices are a way to visualise the performance across classes, allowing the per-class accuracy to be visualised and combinations of classes that are most likely to be misclassified to be understood. All the confusion matrices plotted are the result of cross-validated fitting of class-balanced logistic regression, passing the full datasets into this cross-validation pipeline. This is done using five folds generated using the stratified fold function in scikit-learn [7] which are passed to the cross-validation predict function which predicts and fits across folds, allowing a normalised confusion matrix to be constructed from the predictions on the held-back test sets.

S1.4.5 Visualisation Dimension Reduction Parameters

Before applying dimension reduction methods, we l^2 (euclidean distance) normalise all our vectors. Here we list the parameters used when using non-linear dimension reduction for visualisation of the feature vectors:

- t-SNE - When using t-SNE for visualisation we use the scikit-learn implementation [7] setting a random seed, number of dimensions to 2, perplexity to 20, learning rate to 200, using a cosine distance metric and leaving the rest to the default settings.
- UMAP - When using UMAP for dimension reduction [8], we use a cosine distance metric, set the number of dimensions to 2, the minimum distance to 0.1, the number of neighbours to 30 and a random seed for reproducibility.

For both UMAP and t-SNE parameters are chosen from within the standard ranges used in the literature to balance local and global structure. Both UMAP and t-SNE are stochastic and sensitive to parameter choices, so when used in practice, replotting and parameter testing is advised.

S1.4.6 Clustering Methods and Parameters

Before undertaking any clustering or dimension reduction methods we again ensure that our feature vectors are l^2 (euclidean distance) normalised. We then use PCA or UMAP to reduce the dimension to a number of dimensions amenable to clustering, listed in the main text. When using UMAP for clustering we use the following parameters: we set the number of neighbours to 30, the distance metric to cosine, the minimum distance to 0 and a random seed for reproducibility.

We use the following parameters for our clustering methods:

- k-means clustering - default scikit-learn implementation, no parameters set [7]
- unknown k k-means clustering - default scikit-learn implementation, no parameters set [7] other than the range and use of silhouette score to select the optimal cluster as detailed in the main text.
- Bayesian Gaussian Mixture Model - We use the default setting from Scikit-learn [7], apart from setting the weight concentration prior to 0.1, a random seed and the number of components to $\min(2 \times \#\text{classes}, 50)$.
- HDBSCAN - We use the default settings of the implementation given by the package [9], aside from setting the minimum cluster size to be $\max(5, 0.02 \times \#\text{ samples})$ rounded to the nearest integer.

When visualising clusters we use a visualisation UMAP which reduces to 2 dimensions using number of neighbours equal to 30, minimum distance 0.1, a random seed and a cosine metric.

When performing unsupervised learning in a non-exploratory manner, great care needs to be taken when using non-linear dimension reduction methods such as UMAP [8, 10] as they can distort the underlying distances in the space. Unfortunately, the features outputted by the models on the datasets do not admit an adequate low-dimensional representation that can be found by linear methods, such as PCA.

S1.4.7 Clustering Quantitative Metrics

Clustering methods can be evaluated by comparing the computed clusters with known labels or independently of the labels. For this, we use a variety of metrics from [7]. To compare the clusters with the labels, we use the Adjusted Rand Index (ARI) and Adjusted Mutual Information (AMI) to compare the similarity of the clusters with the true labels while adjusting for overlap from random clustering. We also measure the purity score, which indicates the fraction of points in each cluster which share the most common label in that cluster. To evaluate the clusters without reference to the underlying labels, we use the Silhouette Score, which compares intra-cluster distances to distances to other clusters, and the Davies–Bouldin index (DBI), which compares average intra-cluster spread to cluster separation, with lower values being better and indicating tighter, well-separated clusters.

S1.4.8 Visualising Features Captured by Model

In the main text, we show how we can either use register tokens or patch-level embeddings and PCA to visualise the outputs of the model which we explain in detail below.

In the first approach using register tokens, we pass an image through the preprocessor and the model, configured so that it also outputs the attentions. We then extract all the register tokens across all attention heads (parts of the transformer architecture). We take the median value per patch, to reduce noise and outliers. We then reshape this vector of median register attentions per patch into a square array for visualisation. For visualisation, we then apply a Gaussian blur (with sigma set to 2), clip extreme values (below the 5th and above the 95th percentile), normalise the values between 0 and 1 and resize to the same dimensions as the original image. The attention map can then be overlaid on the original image to give an indication of the parts of the image on which the model is focusing. This, however, is only indicative and not necessarily a true representation of how the model projects objects into feature space. The attention maps involve aggregation across heads, different tokens can give different representations and subsequent non-linearities are applied before the final output. Together, these aspects muddy the direct link between maps and explanation.

The second approach using patch-level RGB PCA works as follows. An image is passed through the preprocessor and model configured to output the hidden states, allowing us to extract the patch embeddings. The patch embeddings are then reduced to 3 dimensions using PCA, normalised between 0 and 1 and then reshaped into a square array per colour channel. To each colour channel, we apply a Gaussian blur (with sigma set to 1). Then we multiply each value by a colour multiplier (set to 2) and pass through a sigmoid function to increase colour vibrancy and allow for better differentiation between patches. We can then resize to match the original image and visualise as an RGB image.

This gives an indication of how the model separates parts of the image into different structures and how it views the image. Again, this comes with caveats: the PCA of the patch-level embeddings loses information; the layer this is applied to affects the representation; the sigmoid and steps to make the visualisation more appealing distort the raw differences in values between patches. However, this still gives a useful check to ensure that the model is segmenting the image in a sensible way.

S1.4.9 Hardware Used

Fitting a logistic regression or kNN classifier is not a computationally expensive process, and fitting a classifier to a dataset of tens of thousands of feature vectors is possible in minutes on a laptop. The runtime of the feature extraction process, which only needs to be run once, will depend on the hardware available. The work on this study was performed using an Apple MacBook Pro M4 Max with 36GB of memory. Using batched image loading and the Apple MPS GPU the smallest data set features could be extracted in a few minutes and the largest in less than 30 minutes. Unsupervised clustering can become runtime intensive depending on the amount of dimensionality reduction, method and dataset size but this must be evaluated on a case-by-case basis.

S1.4.10 Software Versions and Model Id

In this work the following key software versions were used:

- transformers 4.57.0.dev0
- torch 2.8.0
- scikit-learn 1.7.1
- umap-learn 0.5.9.post2
- hdbscan 0.8.39

Tests were performed on the small Dinov3 distilled model “facebook/dinov3-vits16-pretrain-lvd1689m” however the main results of this work used “facebook/dinov3-vitl16-pretrain-lvd1689m”. These model can be downloaded using the Hugging Face [6] library, the DINOv3 models are found in the following collection `https://huggingface.co/collections/facebook/dinov3`.

S1.4.11 Model Selection and Performance

Model selection is a trade-off between accuracy, computational burden, ease of implementation and various other factors. In this work, we generally choose to use the distilled version of DINOv3 with approximately 0.3 billion parameters from the various DINOv3 options. The full DINOv3 model has around 7B parameters and the smallest distilled version around 20 million parameters. For reasons of accessibility, and the fact we want to demonstrate speed and ease of use on lower-end consumer hardware, we do not use the full 7B parameter model. On examining the runtime of the 0.3 billion parameter model, referred to as ViT Large, and the smallest DINOv3 model (which is not a distilled convolutional neural network), table S2, we find that the smaller model is significantly faster but not in a way which makes a huge difference for our task given our dataset sizes. ~ 50 images per second is more than adequate for the tasks we have and the datasets we have available. If you are particularly resource constrained, it is simply the case of updating the model id. Benchmarks are simple timed runs over the datasets and include any warm-up time from model compilation.

Table S2: Comparison of feature extraction runtimes for two DINOv3 model sizes. Numbers depend heavily on hardware setup. A batch size of 64 was used and feature extraction is done using a MacBook Pro with GPU acceleration using bfloat16 precision

Dataset	Images per Second [feature extraction, ViT Large] (s^{-1})	Images per Second [feature extraction, ViT Small] (s^{-1})
Pollen	48.62	397.92
Radiolaria	52.42	333.04
Fossil Tracks	58.86	393.26
Diverse Fossils (RFID)	43.77	183.00
Foraminifera	46.48	216.52

Models will run slower on a CPU however it is not impossibly slow and few-shot testing is certainly possible on a CPU. We test the single-thread, with float32 precision, CPU runtime on the smaller radiolaria and tracks datasets leading to runtimes of 6.54 and 6.35 images per second on the large model and 38.56 and 39.89 images per second using the small model respectively. For a single run, this will be more than feasible, even for a large dataset. However, it may make sense to use a smaller model for initial testing if using a CPU only setup. For inference only, the RAM requirements are modest and accessible on consumer hardware. RAM requirement for the large model running on the CPU or unified Apple memory only never exceed 6GB; and smaller models use even less, hence this is perfectly possible on most systems. Exact benchmark data for all models and datasets for our particular setup is given in the code repository associated with this work.

Switching to a smaller model is not cost free, table S3. In some cases, the smaller models maintain accuracy, but in others there are small drops in performance as the model size is reduced. The small model still does remarkably well, and for the “easier” tasks like the pollen it performs excellently. This reflects

expected intuition regarding DINOv3 performance with model size [5]. Supervised learning results figures and dimension reduction of features generated by smaller models can be found in the code repository.

Table S3: Comparison of maximum classification pipeline accuracy for two Dinov3 model sizes to reported headline literature accuracy. Numbers should be viewed as representative given variance in evaluation protocols. All accuracies from our pipeline are using all the data available and logistic regression unless otherwise noted. Pipeline is identical to that presented in the main text with only the model swapped.

Dataset	Maximum Accuracy [our pipeline, ViT Large] (%)	Representative Headline Literature Accuracy (%) [CNN]	Maximum Accuracy [our pipeline, ViT Small] (%)
Pollen	**98.34**	97.86 [11]	97.78
Radiolaria	**94.30**	88.46 [12]	94.14
Fossil Tracks	81.28	**86** (small test set) [13]	82.26
Diverse Fossils (RFID)	**83.31**	82.97 [14]	77.48 (kNN)
Foraminifera	86.95	87.4 [15], 90.3 [16], **92.0** [17]	80.84

In this work, we do not give CNN time benchmarks as we did not train CNNs for this work. The training requirements of a CNN are well established, although, it will of course vary heavily depending on the dataset size, the model and the amount of augmentations needed. The key point is that CNNs are very restrictive on the number of images that you can train upon if you do not have access to a GPU. Even if you have access to a GPU training will take potentially hours and you may need to repeat if you wish to tune hyperparameters.

S1.5 Feature Visualisation with PCA

In figure S2, we show the application of PCA to reduce the dimensions of the feature vectors. In general, points of the same class are placed together; however, it is difficult to separate the classes as the classes cluster too tightly together. In the case of radiolaria, figure S2c, as classes are relatively easy to classify and there are a relatively small number of classes, the PCA is able to separate out some of the classes. However, for larger datasets with more classes the PCA struggles to be interpretable. In general, we recommend that non-linear dimensionality reduction techniques are likely more effectively used on the DINOv3 vectors; however, there may be cases where PCA gives adequate insight into the data.

S1.6 Pollen Dataset

In this section, we refer to Pollen Dataset as the data of New Zealand Pollen comprising approximately 19,500 images and 46 classes taken from [11], described in the main text.

S1.6.1 Classification Results

The Pollen Dataset, table S4, shows extremely strong results of the classification metrics. With only 10% of the data, all metrics are above 90% and the top-3 accuracy is close to 100% for logistic regression. When the whole dataset is passed to the pipeline, all metrics approach 100% for logistic regression, with kNN also performing very well. This demonstrates that for well separated and distinguishable classes our proposed pipeline can reach the upper limits of possible performance, negating the need for a more complex pipeline.

The confusion matrix for the pollen, figure S3, also shows excellent performance. Most classes have performance in the high 90% range. The lowest performing class *Salix* is confused mostly for *Weinmannia* which looks very similar in certain images, showing errors tend not to be random in nature.

Again, the few-shot results are very strong on the pollen dataset, above 50% in all metrics can be gained with a single example per class. There is a rapid increase in performance with more data, table S5. This highlights the utility of this method for small datasets and proof-of-concept studies.

S1.6.2 Clustering Metrics

When clustering the pollen dataset, table S6, UMAP is generally much better than using PCA as a preprocessing step. Meanwhile, the performance of the clustering method depends on the scale at which it chooses to cluster. If the method chooses a number of clusters which is broadly similar to the true number of classes

Table S4: Pollen Classification results with mean ± standard deviation over folds and repeats

Data Fraction	Classifier	Top-1 Accuracy	Top-2 Accuracy	Top-3 Accuracy	F1 Macro	F1 Weighted	Precision Macro	Recall Macro	Specificity Macro	Balanced Accuracy
0.10	Logistic Regression	0.951 ± 0.013	0.989 ± 0.005	0.995 ± 0.003	0.931 ± 0.022	0.950 ± 0.013	0.943 ± 0.020	0.931 ± 0.022	0.999 ± 0.000	0.931 ± 0.022
0.10	k-Nearest Neighbours	0.887 ± 0.016	0.961 ± 0.009	0.973 ± 0.008	0.825 ± 0.027	0.879 ± 0.018	0.864 ± 0.027	0.816 ± 0.027	0.997 ± 0.000	0.816 ± 0.027
0.30	Logistic Regression	0.972 ± 0.005	0.995 ± 0.002	0.998 ± 0.001	0.967 ± 0.008	0.972 ± 0.005	0.971 ± 0.007	0.966 ± 0.008	0.999 ± 0.000	0.966 ± 0.008
0.30	k-Nearest Neighbours	0.925 ± 0.007	0.977 ± 0.004	0.983 ± 0.003	0.900 ± 0.013	0.924 ± 0.007	0.933 ± 0.011	0.885 ± 0.015	0.998 ± 0.000	0.885 ± 0.015
0.50	Logistic Regression	0.978 ± 0.003	0.997 ± 0.001	**0.999 ± 0.001**	0.973 ± 0.005	0.978 ± 0.003	0.975 ± 0.005	0.972 ± 0.005	0.999 ± 0.000	0.972 ± 0.005
0.50	k-Nearest Neighbours	0.938 ± 0.005	0.981 ± 0.003	0.986 ± 0.003	0.921 ± 0.010	0.938 ± 0.005	0.945 ± 0.009	0.907 ± 0.011	0.999 ± 0.000	0.907 ± 0.011
0.70	Logistic Regression	0.981 ± 0.003	0.997 ± 0.001	0.999 ± 0.001	0.977 ± 0.005	0.981 ± 0.003	0.978 ± 0.005	0.977 ± 0.005	**1.000 ± 0.000**	0.977 ± 0.005
0.70	k-Nearest Neighbours	0.945 ± 0.004	0.984 ± 0.002	0.988 ± 0.002	0.930 ± 0.008	0.944 ± 0.004	0.950 ± 0.007	0.918 ± 0.009	0.999 ± 0.000	0.918 ± 0.009
1.00	Logistic Regression	**0.983 ± 0.002**	**0.998 ± 0.001**	0.999 ± 0.001	**0.980 ± 0.003**	**0.983 ± 0.002**	**0.981 ± 0.003**	**0.980 ± 0.003**	1.000 ± 0.000	**0.980 ± 0.003**
1.00	k-Nearest Neighbours	0.951 ± 0.003	0.986 ± 0.002	0.989 ± 0.002	0.938 ± 0.005	0.950 ± 0.003	0.954 ± 0.004	0.928 ± 0.005	0.999 ± 0.000	0.928 ± 0.005

the metrics which depend on the true labels are generally better, while if the algorithm clusters at a higher level of organisation then the label-independent measures of performance are best. This demonstrates how unsupervised learning can quite successfully identify structure in the pollen dataset; however, there can be variation between algorithms on the scale of clusters detected.

S1.7 Fossil Tracks Dataset

In this section, we discuss the data of [13], described in the main text, which contains approximately 1700 binary images of fossil tracks broken into ornithischians and theropod classes. This dataset additionally contains many images which are fundamentally ambiguous, and it is unclear to which class they should belong.

S1.7.1 Classification Results

When classifying the fossil tracks, table S7, we do not give a top-2 or top-3 accuracy score, as this does not make sense for a binary classification problem. The performance of the classifier is relatively consistent as more data is added as a result of the ambiguity between classes. All metrics plateau near 80%, which is still a reasonably good performance due to the ambiguity. These results highlight that this pipeline cannot necessarily remove fundamental ambiguities in the data and that DINOv3 models trained on a large complex dataset can generalise quite well to binary shapes.

The confusion matrix, figure S4, is unsurprising given what has been observed about this binary classification problem. We classify most of each class correctly, but with a still significant amount of errors falsely placing the object in the opposite class.

Few-shot classification of the tracks paints a similar picture to previous results, table S7. Where again we omit the top-2 and top-3 accuracy. Due to the difficulty of the classification problem, it takes more data to improve beyond the baseline of predicting the most frequent class. The results still demonstrate that few-shot learning is possible and much better than would be possible in a CNN-based approach but that the success of the approach depends on characteristics of the data in question.

S1.7.2 Clustering Metrics

Due to the difficulty of the fossil tracks separability, clustering results are quite poor, table S9. Some algorithms find clusters which are reasonable without respect to the labelling, but due to the fact the data is continuous and does not fall into discrete groups, finding high-quality separable clusters is not possible. It is expected that the found clusters do represent genuine morphological groupings in the data, just ones that are continuous at the boundaries and do not align with the given labels. This data highlights the difficulties of unsupervised clustering, but also how it can inform you about the structure of the data.

Table S5: Few Shot Classification Results - Pollen Dataset

Few-Shot k	Classifier	Repeats Successful	Top-1 Accuracy	Top-2 Accuracy	Top-3 Accuracy	F1 Macro	F1 Weighted	Precision Macro	Recall Macro	Specificity Macro	Balanced Accuracy
1	k-Nearest Neighbours	50	0.540 ± 0.030	0.546 ± 0.029	0.558 ± 0.030	0.511 ± 0.026	0.536 ± 0.033	0.572 ± 0.023	0.550 ± 0.024	0.990 ± 0.001	0.550 ± 0.024
1	Logistic Regression	50	0.570 ± 0.035	0.720 ± 0.032	0.795 ± 0.029	0.538 ± 0.029	0.564 ± 0.036	0.573 ± 0.024	0.580 ± 0.030	0.990 ± 0.001	0.580 ± 0.030
2	k-Nearest Neighbours	50	0.533 ± 0.023	0.767 ± 0.025	0.771 ± 0.024	0.470 ± 0.024	0.500 ± 0.034	0.581 ± 0.020	0.514 ± 0.023	0.989 ± 0.001	0.514 ± 0.023
2	Logistic Regression	50	0.702 ± 0.021	0.838 ± 0.022	0.894 ± 0.018	0.682 ± 0.018	0.702 ± 0.022	0.689 ± 0.018	0.719 ± 0.014	0.993 ± 0.001	0.719 ± 0.014
3	k-Nearest Neighbours	50	0.605 ± 0.024	0.797 ± 0.021	0.863 ± 0.018	0.572 ± 0.022	0.596 ± 0.029	0.643 ± 0.020	0.611 ± 0.020	0.991 ± 0.001	0.611 ± 0.020
3	Logistic Regression	50	0.760 ± 0.018	0.881 ± 0.018	0.925 ± 0.016	0.746 ± 0.015	0.759 ± 0.018	0.743 ± 0.015	0.779 ± 0.012	0.995 ± 0.000	0.779 ± 0.012
5	k-Nearest Neighbours	50	0.695 ± 0.019	0.846 ± 0.017	0.907 ± 0.013	0.660 ± 0.014	0.678 ± 0.020	0.694 ± 0.012	0.693 ± 0.013	0.993 ± 0.000	0.693 ± 0.013
5	Logistic Regression	50	0.818 ± 0.014	0.923 ± 0.013	0.956 ± 0.010	0.809 ± 0.012	0.818 ± 0.015	0.800 ± 0.013	0.839 ± 0.009	0.996 ± 0.000	0.839 ± 0.009
10	k-Nearest Neighbours	50	0.784 ± 0.011	0.909 ± 0.009	0.945 ± 0.008	0.762 ± 0.011	0.775 ± 0.014	0.769 ± 0.010	0.791 ± 0.009	0.995 ± 0.000	0.791 ± 0.009
10	Logistic Regression	50	0.884 ± 0.010	0.964 ± 0.006	0.983 ± 0.004	0.880 ± 0.008	0.884 ± 0.010	0.869 ± 0.010	0.899 ± 0.006	0.997 ± 0.000	0.899 ± 0.006
20	k-Nearest Neighbours	50	0.849 ± 0.007	0.946 ± 0.004	0.967 ± 0.004	0.831 ± 0.007	0.844 ± 0.008	0.826 ± 0.007	0.857 ± 0.006	0.996 ± 0.000	0.857 ± 0.006
20	Logistic Regression	50	**0.926 ± 0.005**	**0.983 ± 0.003**	**0.992 ± 0.002**	**0.923 ± 0.005**	**0.926 ± 0.005**	**0.914 ± 0.006**	**0.935 ± 0.004**	**0.998 ± 0.000**	**0.935 ± 0.004**

S1.8 Radiolaria

The radiolaria data we refer to in the SI is the dataset of [12] referred to in the main text. This data contains 8 classes and 1085 images.

S1.8.1 Classification Results

The radiolaria data is generally very well classified even when using a small fraction of the data, table S10. Using the maximum amount of data both the logistic regression and the kNN classifiers score above 90% in all metrics and scores begin to plateau near the maximum value after using only half the data. This shows our pipeline performs well on distinct objects in small data scenarios.

The confusion matrix for the radiolaria data, figure S5, shows strong performance across all classes aside from *Diamesa* which is confused with *Papalis*. This is unsurprising as, particularly in certain images, both these classes look very similar.

The few-shot classification performance on the radiolaria dataset, table S11, is generally very good with reasonable results across all metrics with only a few examples and a smooth increase in performance with increased data. In particular, the top-2 accuracy is above 90% for only 5 examples in logistic regression showing how the DINOv3 model separates the groups of classes in feature space.

S1.8.2 Clustering Metrics

Again, when clustering the radiolarian dataset, table S12, the UMAP dimension reduction performs much more consistently than the PCA. Clustering results on the radiolaria data are generally solid, however, with the caveat that different algorithms may group some similar species but not others creating a wide variety of possible groupings.

S1.9 Foraminifera

This section of SI refers to the dataset of Foraminifera images taken from [16, 18] which was derived from [15] which contains approximately 27,000 images spread over 35 classes.

Table S6: Clustering results - Pollen Dataset

Pipeline	Algorithm	No. of Clusters	ARI	AMI	Purity	Silhouette	DBI	Noise Fraction	Components Used
PCA	KMeans (Known K)	46	0.4805	0.7568	0.7633	0.1407	1.9950	0.000000	50
PCA	KMeans (Unknown K)	3	0.0909	0.3589	0.1942	0.1689	1.8855	0.000000	50
PCA	Bayesian GMM	50	0.5625	0.8234	0.8363	0.1330	2.0443	0.000000	50
PCA	HDBSCAN	3	0.0122	0.2044	0.0933	0.4010	1.0315	0.787105	50
Exploratory UMAP	KMeans (Known K)	46	0.6298	0.8711	**0.8753**	0.6332	0.5032	0.000000	15
Exploratory UMAP	KMeans (Unknown K)	12	0.3312	0.7203	0.4450	**0.7527**	**0.2995**	0.000000	15
Exploratory UMAP	Bayesian GMM	29	**0.6833**	**0.8915**	0.7258	0.6702	0.3459	0.000000	15
Exploratory UMAP	HDBSCAN	12	0.3801	0.7470	0.4757	0.6863	0.3199	0.089948	15

Table S7: Classification results with mean ± standard deviation over folds and repeats - Fossil Tracks Dataset

Data Fraction	Classifier	Top-1 Accuracy	Top-2 Accuracy	Top-3 Accuracy	F1 Macro	F1 Weighted	Precision Macro	Recall Macro	Specificity Macro	Balanced Accuracy
0.10	Logistic Regression	0.775 ± 0.057	N/A	N/A	0.765 ± 0.063	0.773 ± 0.060	0.773 ± 0.058	0.767 ± 0.064	0.767 ± 0.064	0.767 ± 0.064
0.10	k-Nearest Neighbours	0.755 ± 0.074	N/A	N/A	0.741 ± 0.079	0.751 ± 0.076	0.758 ± 0.083	0.740 ± 0.077	0.740 ± 0.077	0.740 ± 0.077
0.30	Logistic Regression	0.801 ± 0.046	N/A	N/A	0.794 ± 0.047	0.800 ± 0.046	0.797 ± 0.048	0.795 ± 0.046	0.795 ± 0.046	0.795 ± 0.046
0.30	k-Nearest Neighbours	0.772 ± 0.044	N/A	N/A	0.760 ± 0.048	0.769 ± 0.046	0.771 ± 0.048	0.757 ± 0.047	0.757 ± 0.047	0.757 ± 0.047
0.50	Logistic Regression	0.812 ± 0.028	N/A	N/A	0.806 ± 0.029	0.812 ± 0.028	**0.808 ± 0.029**	0.807 ± 0.028	0.807 ± 0.028	0.807 ± 0.028
0.50	k-Nearest Neighbours	0.784 ± 0.028	N/A	N/A	0.773 ± 0.030	0.781 ± 0.029	0.782 ± 0.030	0.769 ± 0.030	0.769 ± 0.030	0.769 ± 0.030
0.70	Logistic Regression	0.812 ± 0.024	N/A	N/A	**0.807 ± 0.025**	0.812 ± 0.024	0.807 ± 0.025	**0.808 ± 0.026**	**0.808 ± 0.026**	**0.808 ± 0.026**
0.70	k-Nearest Neighbours	0.790 ± 0.027	N/A	N/A	0.780 ± 0.028	0.788 ± 0.027	0.788 ± 0.029	0.776 ± 0.028	0.776 ± 0.028	0.776 ± 0.028
1.00	Logistic Regression	**0.813 ± 0.022**	N/A	N/A	0.807 ± 0.023	**0.813 ± 0.022**	0.808 ± 0.023	0.808 ± 0.022	0.808 ± 0.022	0.808 ± 0.022
1.00	k-Nearest Neighbours	0.799 ± 0.024	N/A	N/A	0.789 ± 0.025	0.797 ± 0.024	0.796 ± 0.025	0.786 ± 0.026	0.786 ± 0.026	0.786 ± 0.026

S1.9.1 Classification Results

As the foraminifera dataset is relatively large, performance, table S13, is good with only a small fraction of the data used, and the high top-3 accuracy shows it can quickly learn to group similar foraminifera. This shows that supervised classification can still perform well despite differing views of the foraminifera and differences in image lighting conditions.

The confusion matrix, figure S6, shows generally good performance across most classes but a few outliers with very poor performance. The very poorly performing classes generally have a very small number of images, which explains why the pipeline struggles to separate them as effectively as the larger classes.

Few-shot performance, table S14, is weaker on the foraminifera dataset since it takes more examples for the pipeline to build a representative set of images for each class. However, top-3 accuracy does become reasonable, showing how some understanding of the data is found. Few-shot performance is still indicative that these objects could be classified given more data and could be useful in a proof-of-concept approach.

S1.9.2 Clustering Metrics

The outcome of the clustering of the foraminifera data, table S15, depends heavily on the chosen algorithm and dimension reduction method. UMAP is generally better than PCA, with PCA based HDBSCAN failing completely. Some clustering methods, such as unknown k k-means and HDBSCAN, focus on the two large clusters caused by imaging artifacts, highlighting potential caveats with this approach. Meanwhile, the Bayesian Gaussian Mixture Model finds a set of clusters which partially align with the underlying labels. This shows how care needs to be taken not to identify image artifacts with this methodology; however,

Table S8: Few Shot Classification Results - Fossils Tracks Dataset

Few-Shot k	Classifier	Repeats Successful	Top-1 Accuracy	Top-2 Accuracy	Top-3 Accuracy	F1 Macro	F1 Weighted	Precision Macro	Recall Macro	Specificity Macro	Balanced Accuracy
1	k-Nearest Neighbours	50	0.561 ± 0.104	N/A	N/A	0.515 ± 0.102	0.531 ± 0.109	0.564 ± 0.107	0.541 ± 0.087	0.541 ± 0.087	0.541 ± 0.087
1	Logistic Regression	50	0.590 ± 0.093	N/A	N/A	0.550 ± 0.101	0.565 ± 0.104	0.600 ± 0.100	0.573 ± 0.082	0.573 ± 0.082	0.573 ± 0.082
2	k-Nearest Neighbours	50	0.609 ± 0.052	N/A	N/A	0.468 ± 0.115	0.449 ± 0.132	0.567 ± 0.093	0.554 ± 0.066	0.554 ± 0.066	0.554 ± 0.066
2	Logistic Regression	50	0.605 ± 0.093	N/A	N/A	0.584 ± 0.098	0.591 ± 0.102	0.613 ± 0.091	0.601 ± 0.085	0.601 ± 0.085	0.601 ± 0.085
3	k-Nearest Neighbours	50	0.601 ± 0.077	N/A	N/A	0.568 ± 0.081	0.580 ± 0.084	0.614 ± 0.078	0.588 ± 0.066	0.588 ± 0.066	0.588 ± 0.066
3	Logistic Regression	50	0.625 ± 0.068	N/A	N/A	0.608 ± 0.068	0.619 ± 0.068	0.623 ± 0.070	0.614 ± 0.064	0.614 ± 0.064	0.614 ± 0.064
5	k-Nearest Neighbours	50	0.625 ± 0.066	N/A	N/A	0.593 ± 0.077	0.608 ± 0.075	0.628 ± 0.070	0.606 ± 0.061	0.606 ± 0.061	0.606 ± 0.061
5	Logistic Regression	50	0.642 ± 0.061	N/A	N/A	0.629 ± 0.060	0.638 ± 0.063	0.645 ± 0.057	0.637 ± 0.052	0.637 ± 0.052	0.637 ± 0.052
10	k-Nearest Neighbours	50	0.641 ± 0.048	N/A	N/A	0.625 ± 0.048	0.637 ± 0.048	0.635 ± 0.048	0.629 ± 0.045	0.629 ± 0.045	0.629 ± 0.045
10	Logistic Regression	50	0.674 ± 0.058	N/A	N/A	0.668 ± 0.056	0.674 ± 0.057	0.674 ± 0.054	0.674 ± 0.053	0.674 ± 0.053	0.674 ± 0.053
20	k-Nearest Neighbours	50	0.667 ± 0.050	N/A	N/A	0.658 ± 0.046	0.666 ± 0.049	0.666 ± 0.044	0.663 ± 0.041	0.663 ± 0.041	0.663 ± 0.041
20	Logistic Regression	50	**0.718 ± 0.031**	N/A	N/A	**0.713 ± 0.030**	**0.719 ± 0.031**	**0.716 ± 0.029**	**0.719 ± 0.028**	**0.719 ± 0.028**	**0.719 ± 0.028**

this could be remedied by clustering on the large macroscopic clusters again individually and inspecting the clusters to find alignment.

S1.10 Diverse Palaeontological Images Dataset (RFID)

This section in the SI concerns the diverse RFID [14], described in the main text, which contains approximately 60,000 images taken from the Internet comprising 50 classes.

S1.10.1 Classification Results - Diverse Palaeontological Images

Classification performance is generally very good on the RFID, table S16, given the variations in image quality in this dataset. However, as DINOv3 is trained on such a large volume of images, this is the scenario in which it is expected to perform well. Interestingly, on this data the kNN classifier and the logistic regression perform very similarly, with kNN even exceeding the performance of the logistic regression on some metrics. This is likely due to how the DINOv3 feature vectors separate the diverse objects in feature space.

The confusion matrix, figure S7, shows generally very good performance across most classes, with performance being weakest on classes which lack strong distinct structures such as bone fragments, bryozoans, sponges and trace fossils. The classifier also struggles to distinguish bivalves and brachiopods which demonstrates that the classifier behaves in a somewhat predictable manner as these are both classes which are difficult for people to distinguish.

Given the diversity of image types within each class, it takes several examples for few-shot performance, table S17, to begin to increase to reasonable levels. However, with enough examples we see adequate few shot performance even on a dataset containing as much diversity as this.

S1.10.2 Clustering Metrics

Clustering of the diverse images, table S18, again shows UMAP performs better than PCA, with PCA HDBSCAN failing. The algorithms are able to find moderately good clusters. However, these do not necessarily reflect the labelling, as in a dataset as diverse, in species and representation, there will be many ways to group the objects.

Table S9: Clustering results - Fossil Tracks Dataset

Pipeline	Algorithm	No. of Clusters	ARI	AMI	Purity	Silhouette	DBI	Noise Fraction	Components Used
PCA	KMeans (Known K)	2	0.0385	0.0256	0.5990	0.1640	2.1381	0.000000	50
PCA	KMeans (Unknown K)	2	0.0385	0.0256	0.5990	0.1640	2.1381	0.000000	50
PCA	Bayesian GMM	4	0.0493	0.0378	0.6290	0.1187	2.4838	0.000000	50
PCA	HDBSCAN	2	0.0003	0.0489	0.1305	0.4298	0.7397	0.833633	50
Exploratory UMAP	KMeans (Known K)	2	0.0102	0.0019	0.5859	0.5333	0.6047	0.000000	15
Exploratory UMAP	KMeans (Unknown K)	3	**0.0973**	0.0612	**0.6517**	**0.5396**	0.6413	0.000000	15
Exploratory UMAP	Bayesian GMM	4	0.0817	0.0500	0.6349	0.4360	0.7782	0.000000	15
Exploratory UMAP	HDBSCAN	3	0.0251	**0.0660**	0.6236	0.4906	**0.4666**	0.008977	15

Table S10: Classification results with mean ± standard deviation over folds and repeats - Radiolaria Dataset

Data Fraction	Classifier	Top-1 Accuracy	Top-2 Accuracy	Top-3 Accuracy	F1 Macro	F1 Weighted	Precision Macro	Recall Macro	Specificity Macro	Balanced Accuracy
0.10	Logistic Regression	0.859 ± 0.068	0.969 ± 0.037	0.989 ± 0.022	0.803 ± 0.094	0.840 ± 0.072	0.815 ± 0.099	0.824 ± 0.084	0.979 ± 0.010	0.824 ± 0.084
0.10	k-Nearest Neighbours	0.775 ± 0.069	0.924 ± 0.053	0.960 ± 0.045	0.645 ± 0.120	0.734 ± 0.091	0.647 ± 0.139	0.670 ± 0.108	0.965 ± 0.012	0.670 ± 0.108
0.30	Logistic Regression	0.917 ± 0.029	0.990 ± 0.010	0.998 ± 0.005	0.904 ± 0.037	0.915 ± 0.030	0.922 ± 0.033	0.900 ± 0.038	0.987 ± 0.004	0.900 ± 0.038
0.30	k-Nearest Neighbours	0.871 ± 0.035	0.975 ± 0.020	0.986 ± 0.013	0.816 ± 0.058	0.852 ± 0.041	0.875 ± 0.061	0.804 ± 0.057	0.979 ± 0.006	0.804 ± 0.057
0.50	Logistic Regression	0.932 ± 0.019	0.992 ± 0.009	**0.999 ± 0.003**	0.926 ± 0.023	0.931 ± 0.020	0.932 ± 0.021	0.924 ± 0.026	0.990 ± 0.003	0.924 ± 0.026
0.50	k-Nearest Neighbours	0.895 ± 0.030	0.981 ± 0.015	0.989 ± 0.010	0.866 ± 0.041	0.887 ± 0.032	0.907 ± 0.039	0.852 ± 0.042	0.983 ± 0.005	0.852 ± 0.042
0.70	Logistic Regression	0.938 ± 0.019	0.993 ± 0.006	0.999 ± 0.003	0.935 ± 0.021	0.938 ± 0.019	0.940 ± 0.019	0.934 ± 0.024	**0.991 ± 0.003**	0.934 ± 0.024
0.70	k-Nearest Neighbours	0.910 ± 0.020	0.984 ± 0.008	0.991 ± 0.006	0.888 ± 0.029	0.902 ± 0.023	0.919 ± 0.020	0.875 ± 0.031	0.985 ± 0.003	0.875 ± 0.031
1.00	Logistic Regression	**0.943 ± 0.015**	**0.994 ± 0.005**	0.999 ± 0.002	**0.940 ± 0.016**	**0.943 ± 0.015**	**0.942 ± 0.017**	**0.940 ± 0.017**	0.991 ± 0.002	**0.940 ± 0.017**
1.00	k-Nearest Neighbours	0.921 ± 0.018	0.985 ± 0.008	0.991 ± 0.005	0.901 ± 0.023	0.913 ± 0.018	0.924 ± 0.018	0.888 ± 0.025	0.987 ± 0.003	0.888 ± 0.025

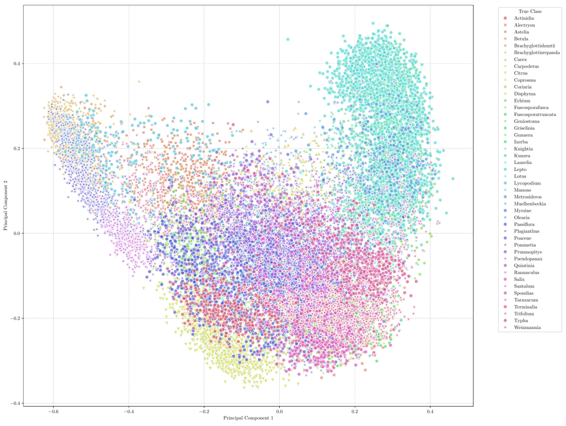

(a) Pollen

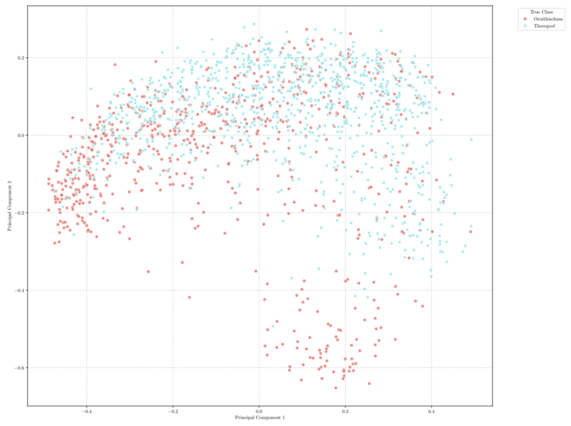

(b) Fossil Tracks

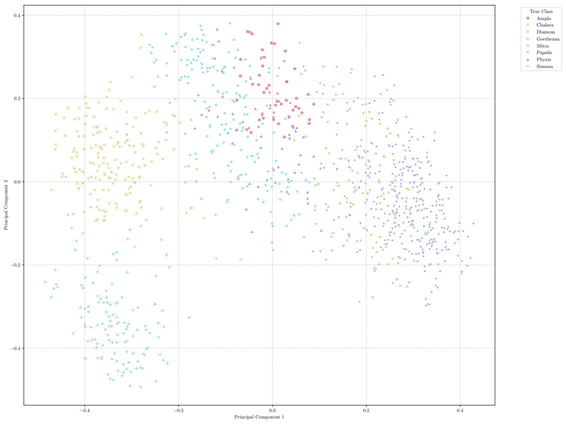

(c) Radiolaria

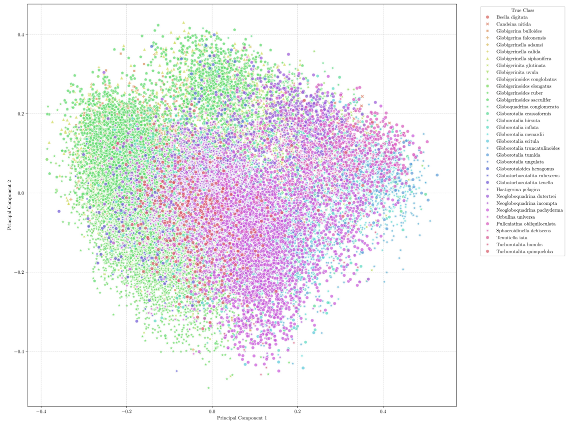

(d) Foraminifera

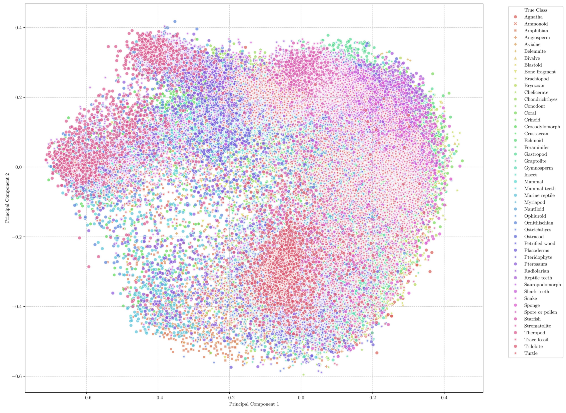

(e) Diverse Palaeontological Images

Figure S2: Dimension reduction of feature vectors by PCA for all Datasets and all Images. Points are labelled by the true class.

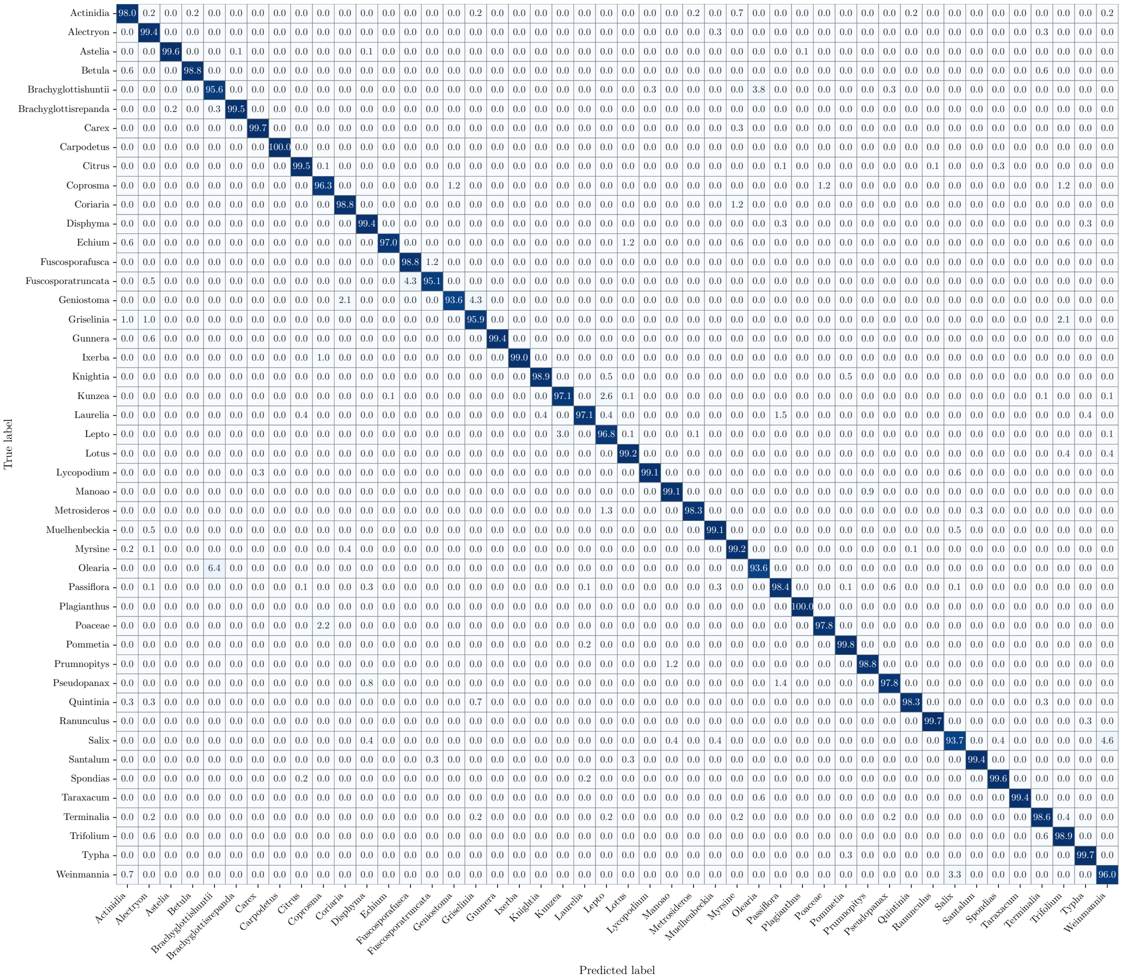

Figure S3: Confusion Matrix, Pollen Dataset

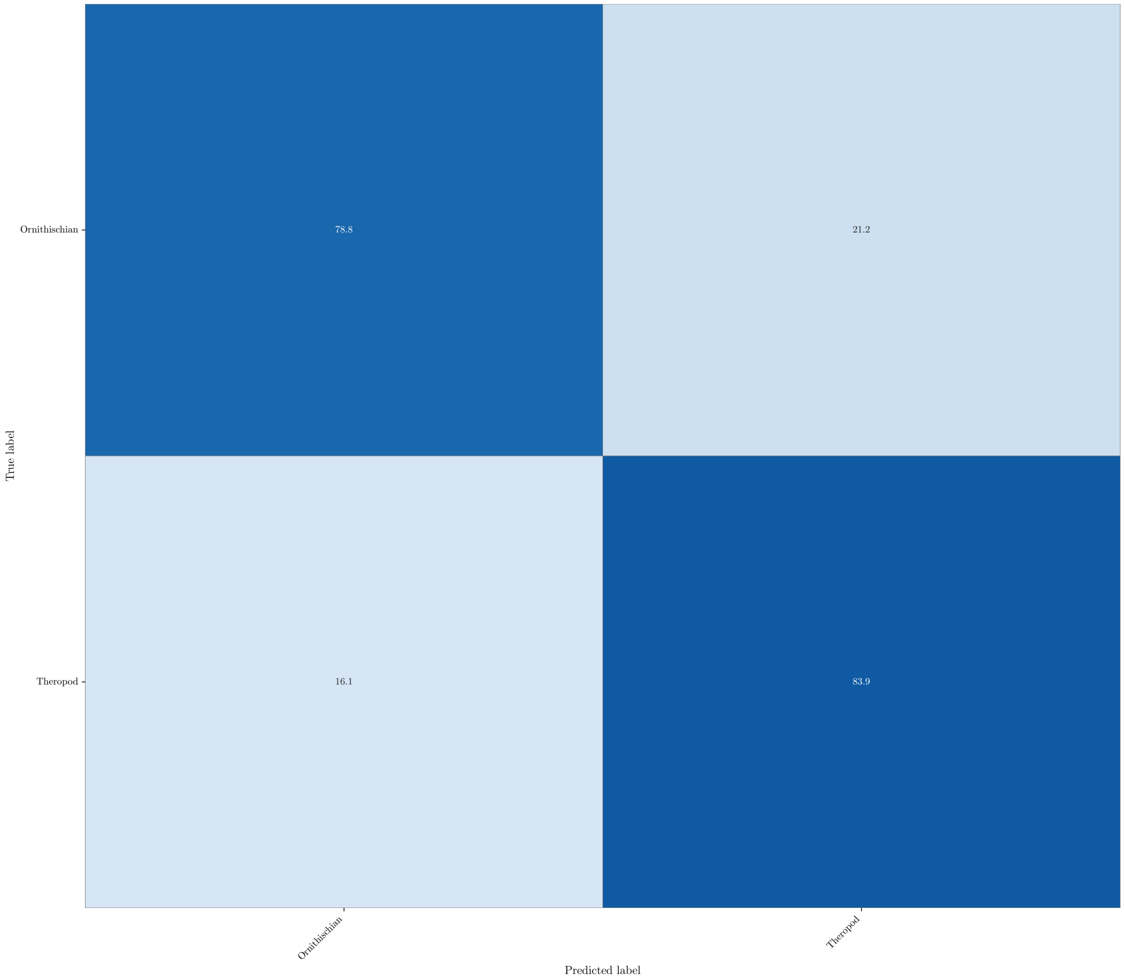

Figure S4: Confusion Matrix - Fossil Tracks Dataset

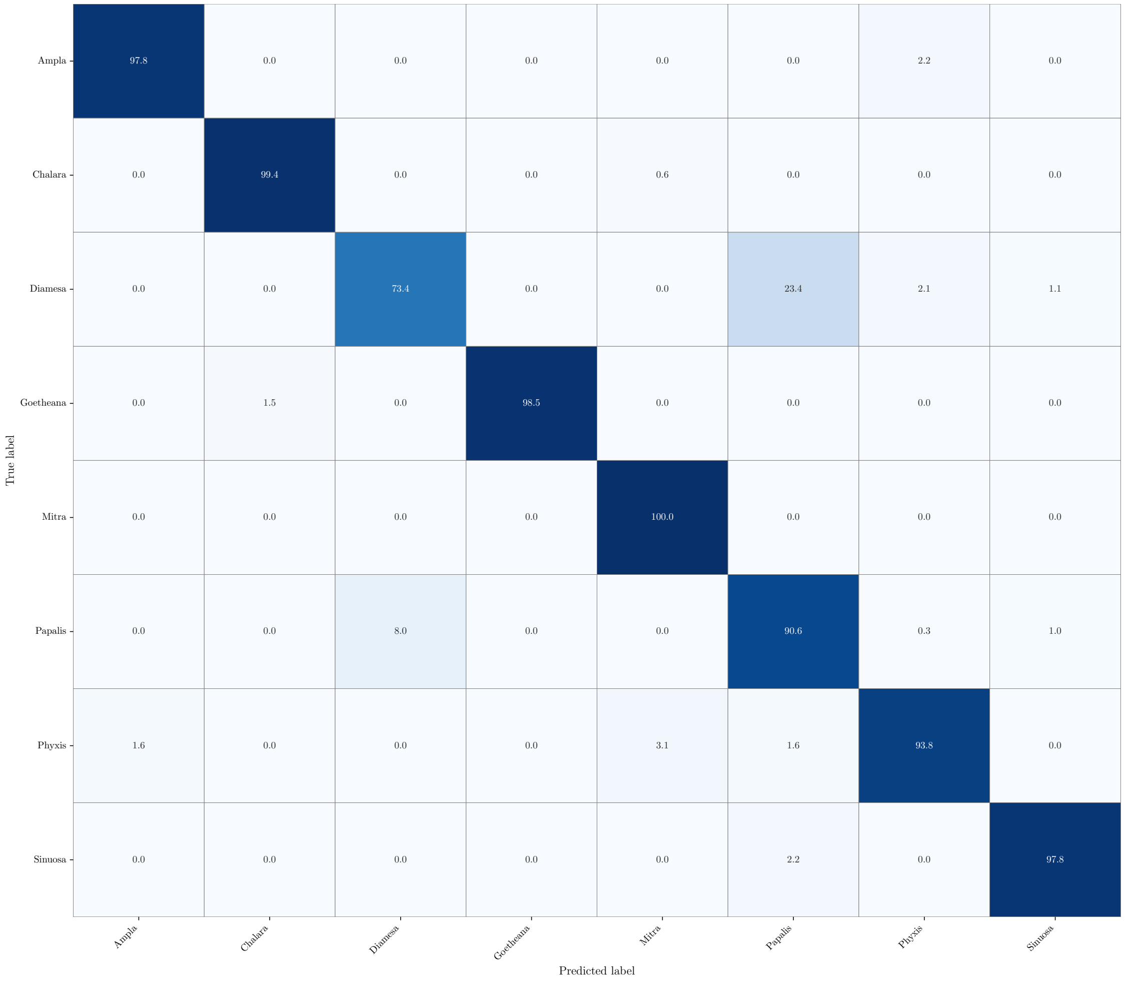

Figure S5: Confusion Matrix - Radiolarians Dataset

Table S11: Few-Shot Classification Results - Radiolaria Dataset

Few-Shot k	Classifier	Repeats Successful	Top-1 Accuracy	Top-2 Accuracy	Top-3 Accuracy	F1 Macro	F1 Weighted	Precision Macro	Recall Macro	Specificity Macro	Balanced Accuracy
1	k-Nearest Neighbours	50	0.562 ± 0.065	0.598 ± 0.071	0.635 ± 0.068	0.536 ± 0.053	0.564 ± 0.069	0.590 ± 0.048	0.568 ± 0.050	0.938 ± 0.009	0.568 ± 0.050
1	Logistic Regression	50	0.579 ± 0.063	0.780 ± 0.057	0.885 ± 0.040	0.550 ± 0.051	0.580 ± 0.064	0.586 ± 0.047	0.583 ± 0.045	0.940 ± 0.008	0.583 ± 0.045
2	k-Nearest Neighbours	50	0.522 ± 0.060	0.802 ± 0.053	0.820 ± 0.052	0.498 ± 0.066	0.524 ± 0.074	0.589 ± 0.057	0.553 ± 0.048	0.934 ± 0.009	0.553 ± 0.048
2	Logistic Regression	50	0.654 ± 0.043	0.843 ± 0.038	0.926 ± 0.029	0.638 ± 0.040	0.659 ± 0.042	0.656 ± 0.040	0.667 ± 0.039	0.951 ± 0.006	0.667 ± 0.039
3	k-Nearest Neighbours	50	0.589 ± 0.046	0.788 ± 0.048	0.913 ± 0.034	0.582 ± 0.037	0.603 ± 0.049	0.644 ± 0.043	0.631 ± 0.029	0.944 ± 0.005	0.631 ± 0.029
3	Logistic Regression	50	0.709 ± 0.044	0.883 ± 0.036	0.951 ± 0.023	0.701 ± 0.041	0.715 ± 0.044	0.711 ± 0.039	0.730 ± 0.031	0.959 ± 0.006	0.730 ± 0.031
5	k-Nearest Neighbours	50	0.678 ± 0.036	0.876 ± 0.030	0.954 ± 0.016	0.658 ± 0.036	0.680 ± 0.039	0.688 ± 0.042	0.686 ± 0.030	0.953 ± 0.005	0.686 ± 0.030
5	Logistic Regression	50	0.768 ± 0.032	0.925 ± 0.024	0.975 ± 0.013	0.763 ± 0.030	0.775 ± 0.032	0.764 ± 0.029	0.788 ± 0.028	0.967 ± 0.004	0.788 ± 0.028
10	k-Nearest Neighbours	50	0.764 ± 0.029	0.926 ± 0.020	0.973 ± 0.010	0.755 ± 0.023	0.765 ± 0.029	0.777 ± 0.026	0.776 ± 0.021	0.965 ± 0.004	0.776 ± 0.021
10	Logistic Regression	50	0.837 ± 0.029	0.965 ± 0.014	0.991 ± 0.005	0.838 ± 0.026	0.843 ± 0.027	0.834 ± 0.025	0.858 ± 0.022	0.977 ± 0.004	0.858 ± 0.022
20	k-Nearest Neighbours	50	0.812 ± 0.021	0.951 ± 0.013	0.980 ± 0.009	0.816 ± 0.018	0.819 ± 0.023	0.824 ± 0.017	0.841 ± 0.020	0.973 ± 0.004	0.841 ± 0.020
20	Logistic Regression	50	**0.882 ± 0.017**	**0.982 ± 0.005**	**0.996 ± 0.002**	**0.883 ± 0.015**	**0.887 ± 0.015**	**0.877 ± 0.014**	**0.900 ± 0.016**	**0.983 ± 0.002**	**0.900 ± 0.016**

Table S12: Clustering Results - Radiolaria Dataset

Pipeline	Algorithm	No. of Clusters	ARI	AMI	Purity	Silhouette	DBI	Noise Fraction	Components Used
PCA	KMeans (Known K)	8	0.4768	0.6210	0.7327	0.1387	2.0850	0.000000	50
PCA	KMeans (Unknown K)	3	0.4019	0.5681	0.5456	0.1979	1.7619	0.000000	50
PCA	Bayesian GMM	16	0.3288	0.5685	0.7558	0.0842	2.6096	0.000000	50
PCA	HDBSCAN	2	0.0719	0.1634	0.2378	0.2303	1.2640	0.456221	50
Exploratory UMAP	KMeans (Known K)	8	0.5193	0.6740	0.7475	0.6255	0.5220	0.000000	15
Exploratory UMAP	KMeans (Unknown K)	4	0.5643	0.7252	0.7051	**0.7234**	**0.3704**	0.000000	15
Exploratory UMAP	Bayesian GMM	15	0.4907	0.6895	**0.8544**	0.4300	0.8286	0.000000	15
Exploratory UMAP	HDBSCAN	5	**0.6122**	**0.7607**	0.7475	0.6809	0.4270	0.000000	15

Table S13: Classification results with mean ± standard deviation over folds and repeats - Foraminifera Dataset

Data Fraction	Classifier	Top-1 Accuracy	Top-2 Accuracy	Top-3 Accuracy	F1 Macro	F1 Weighted	Precision Macro	Recall Macro	Specificity Macro	Balanced Accuracy
0.10	Logistic Regression	0.810 ± 0.016	0.917 ± 0.012	0.955 ± 0.008	0.671 ± 0.029	0.811 ± 0.015	0.683 ± 0.032	0.687 ± 0.026	0.994 ± 0.001	0.708 ± 0.022
0.10	k-Nearest Neighbours	0.711 ± 0.018	0.826 ± 0.015	0.872 ± 0.014	0.506 ± 0.029	0.690 ± 0.015	0.584 ± 0.041	0.488 ± 0.030	0.991 ± 0.001	0.493 ± 0.030
0.30	Logistic Regression	0.844 ± 0.009	0.939 ± 0.005	0.968 ± 0.004	0.716 ± 0.032	0.846 ± 0.009	0.717 ± 0.037	0.737 ± 0.034	0.995 ± 0.000	0.751 ± 0.034
0.30	k-Nearest Neighbours	0.756 ± 0.010	0.861 ± 0.008	0.899 ± 0.007	0.549 ± 0.028	0.745 ± 0.010	0.643 ± 0.041	0.517 ± 0.026	0.992 ± 0.000	0.518 ± 0.027
0.50	Logistic Regression	0.856 ± 0.007	0.947 ± 0.004	0.973 ± 0.003	0.726 ± 0.021	0.859 ± 0.007	0.715 ± 0.021	0.752 ± 0.025	**0.996 ± 0.000**	0.755 ± 0.024
0.50	k-Nearest Neighbours	0.777 ± 0.007	0.876 ± 0.006	0.912 ± 0.006	0.568 ± 0.017	0.768 ± 0.007	0.670 ± 0.027	0.530 ± 0.015	0.993 ± 0.000	0.530 ± 0.015
0.70	Logistic Regression	0.863 ± 0.005	0.951 ± 0.004	0.976 ± 0.003	0.740 ± 0.026	0.866 ± 0.005	0.724 ± 0.028	0.770 ± 0.028	0.996 ± 0.000	0.770 ± 0.028
0.70	k-Nearest Neighbours	0.787 ± 0.005	0.884 ± 0.004	0.917 ± 0.004	0.584 ± 0.022	0.779 ± 0.005	0.683 ± 0.029	0.546 ± 0.021	0.993 ± 0.000	0.546 ± 0.021
1.00	Logistic Regression	**0.870 ± 0.004**	**0.956 ± 0.002**	**0.979 ± 0.002**	**0.770 ± 0.022**	**0.873 ± 0.004**	**0.748 ± 0.022**	**0.809 ± 0.026**	0.996 ± 0.000	**0.809 ± 0.026**
1.00	k-Nearest Neighbours	0.797 ± 0.004	0.892 ± 0.003	0.924 ± 0.003	0.603 ± 0.018	0.790 ± 0.003	0.703 ± 0.024	0.564 ± 0.017	0.994 ± 0.000	0.564 ± 0.017

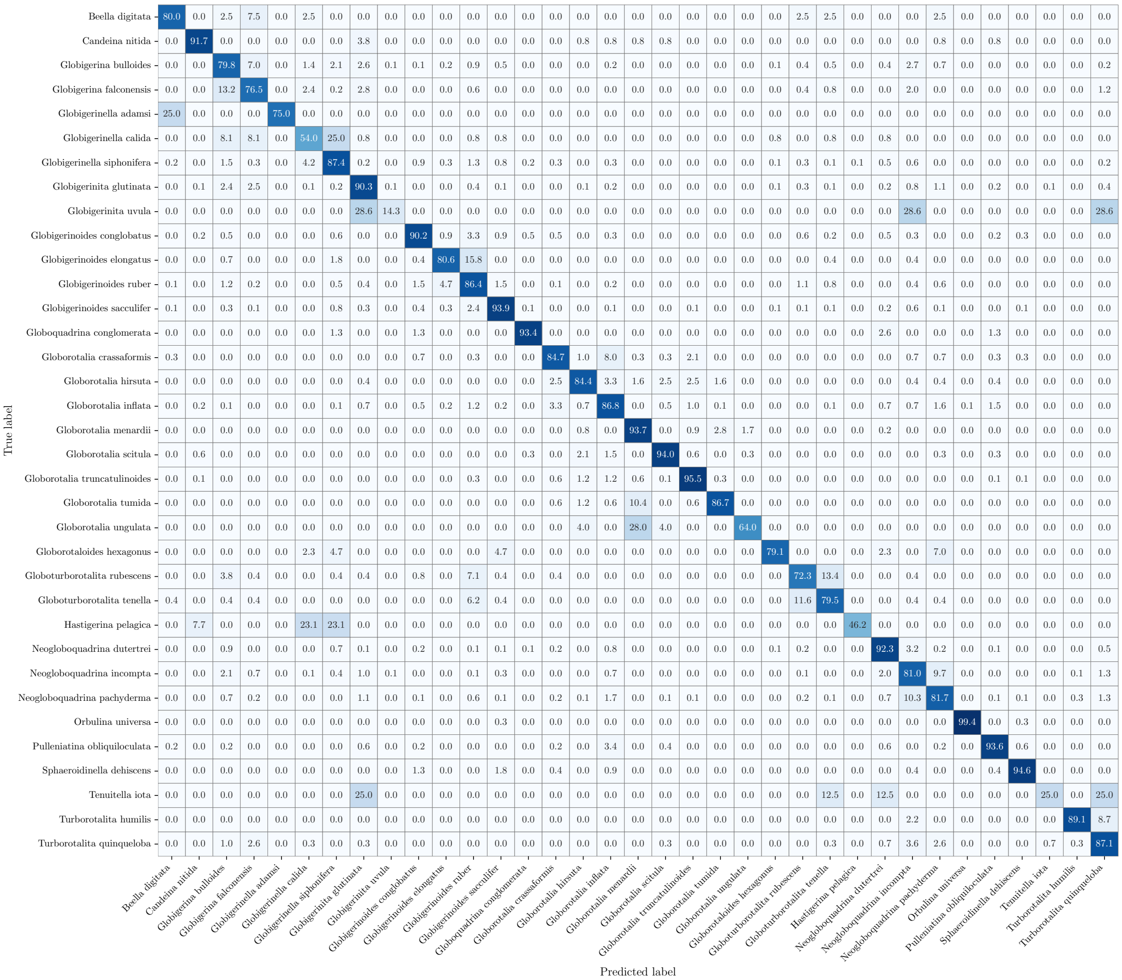

Figure S6: Confusion Matrix - Foraminifera

Table S14: Few-Shot Classification Results - Foraminifera

Few-Shot k	Classifier	Repeats Successful	Top-1 Accuracy	Top-2 Accuracy	Top-3 Accuracy	F1 Macro	F1 Weighted	Precision Macro	Recall Macro	Specificity Macro	Balanced Accuracy
1	k-Nearest Neighbours	50	0.218 ± 0.032	0.254 ± 0.030	0.384 ± 0.036	0.193 ± 0.016	0.238 ± 0.038	0.257 ± 0.022	0.305 ± 0.023	0.977 ± 0.001	0.305 ± 0.023
1	Logistic Regression	50	0.221 ± 0.033	0.338 ± 0.045	0.422 ± 0.050	0.194 ± 0.017	0.245 ± 0.042	0.235 ± 0.021	0.333 ± 0.021	0.977 ± 0.001	0.333 ± 0.021
2	k-Nearest Neighbours	50	0.224 ± 0.039	0.385 ± 0.047	0.470 ± 0.034	0.170 ± 0.018	0.217 ± 0.038	0.252 ± 0.024	0.279 ± 0.019	0.976 ± 0.001	0.279 ± 0.019
2	Logistic Regression	50	0.310 ± 0.032	0.457 ± 0.036	0.551 ± 0.036	0.265 ± 0.018	0.346 ± 0.039	0.289 ± 0.021	0.441 ± 0.023	0.980 ± 0.001	0.441 ± 0.023
3	k-Nearest Neighbours	50	0.225 ± 0.027	0.382 ± 0.032	0.512 ± 0.035	0.197 ± 0.015	0.223 ± 0.035	0.289 ± 0.019	0.330 ± 0.019	0.977 ± 0.001	0.330 ± 0.019
3	Logistic Regression	50	0.364 ± 0.031	0.518 ± 0.037	0.614 ± 0.036	0.307 ± 0.014	0.401 ± 0.036	0.323 ± 0.015	0.506 ± 0.016	0.981 ± 0.001	0.506 ± 0.016
5	k-Nearest Neighbours	50	0.278 ± 0.029	0.439 ± 0.030	0.556 ± 0.031	0.251 ± 0.015	0.304 ± 0.038	0.315 ± 0.015	0.402 ± 0.022	0.979 ± 0.001	0.402 ± 0.022
5	Logistic Regression	50	0.433 ± 0.028	0.599 ± 0.028	0.693 ± 0.025	0.367 ± 0.013	0.475 ± 0.030	0.371 ± 0.013	0.584 ± 0.018	0.983 ± 0.001	0.584 ± 0.018
10	k-Nearest Neighbours	50	0.358 ± 0.025	0.528 ± 0.027	0.638 ± 0.026	0.321 ± 0.014	0.394 ± 0.031	0.358 ± 0.013	0.499 ± 0.024	0.981 ± 0.001	0.499 ± 0.024
10	Logistic Regression	50	0.534 ± 0.018	0.702 ± 0.016	0.786 ± 0.014	0.437 ± 0.011	0.575 ± 0.017	0.423 ± 0.010	0.669 ± 0.020	0.986 ± 0.001	0.669 ± 0.020
20	k-Nearest Neighbours	50	0.435 ± 0.017	0.605 ± 0.015	0.706 ± 0.014	0.372 ± 0.016	0.473 ± 0.020	0.391 ± 0.015	0.559 ± 0.025	0.983 ± 0.001	0.559 ± 0.025
20	Logistic Regression	50	**0.616 ± 0.014**	**0.775 ± 0.013**	**0.848 ± 0.011**	**0.489 ± 0.012**	**0.650 ± 0.014**	**0.465 ± 0.013**	**0.723 ± 0.022**	**0.989 ± 0.000**	**0.723 ± 0.022**

Table S15: Clustering Results - Foraminifera

Pipeline	Algorithm	No. of Clusters	ARI	AMI	Purity	Silhouette	DBI	Noise Fraction	Components Used
PCA	KMeans (Known K)	35	0.1788	0.4256	0.5771	0.0711	2.5075	0.000000	50
PCA	KMeans (Unknown K)	2	0.0923	0.1556	0.2665	0.1153	2.7967	0.000000	50
PCA	Bayesian GMM	50	0.1846	0.4871	0.6574	0.0379	2.8144	0.000000	50
PCA	HDBSCAN	0	0.0000	0.0000	0.0000	nan	nan	1.000000	50
Exploratory UMAP	KMeans (Known K)	35	0.2505	0.5177	**0.6643**	0.4040	0.8222	0.000000	15
Exploratory UMAP	KMeans (Unknown K)	2	0.0466	0.0951	0.2259	**0.7883**	**0.3012**	0.000000	15
Exploratory UMAP	Bayesian GMM	38	**0.4172**	**0.5456**	0.5886	-0.0011	1.2859	0.000000	15
Exploratory UMAP	HDBSCAN	3	0.0560	0.1359	0.2419	0.6234	0.3518	0.006744	15

Table S16: Classification Results with mean ± standard deviation over folds and repeats - RFID

Data Fraction	Classifier	Top-1 Accuracy	Top-2 Accuracy	Top-3 Accuracy	F1 Macro	F1 Weighted	Precision Macro	Recall Macro	Specificity Macro	Balanced Accuracy
0.10	Logistic Regression	0.788 ± 0.010	0.880 ± 0.009	0.916 ± 0.008	0.790 ± 0.010	0.789 ± 0.010	0.798 ± 0.010	0.789 ± 0.010	0.996 ± 0.000	0.789 ± 0.010
0.10	k-Nearest Neighbours	0.734 ± 0.013	0.827 ± 0.010	0.864 ± 0.010	0.736 ± 0.011	0.735 ± 0.011	0.753 ± 0.011	0.736 ± 0.012	0.995 ± 0.000	0.736 ± 0.012
0.30	Logistic Regression	0.817 ± 0.007	0.900 ± 0.005	0.931 ± 0.004	0.818 ± 0.006	0.817 ± 0.006	0.822 ± 0.006	0.817 ± 0.007	0.996 ± 0.000	0.817 ± 0.007
0.30	k-Nearest Neighbours	0.788 ± 0.008	0.866 ± 0.006	0.896 ± 0.006	0.790 ± 0.007	0.789 ± 0.007	0.798 ± 0.007	0.790 ± 0.007	0.996 ± 0.000	0.790 ± 0.007
0.50	Logistic Regression	0.825 ± 0.006	0.905 ± 0.005	0.935 ± 0.004	0.827 ± 0.006	0.826 ± 0.006	0.829 ± 0.005	0.826 ± 0.006	0.996 ± 0.000	0.826 ± 0.006
0.50	k-Nearest Neighbours	0.809 ± 0.005	0.881 ± 0.004	0.908 ± 0.003	0.811 ± 0.005	0.810 ± 0.005	0.817 ± 0.005	0.810 ± 0.005	0.996 ± 0.000	0.810 ± 0.005
0.70	Logistic Regression	0.829 ± 0.003	0.908 ± 0.003	0.938 ± 0.002	0.831 ± 0.003	0.830 ± 0.003	0.833 ± 0.003	0.830 ± 0.003	**0.997 ± 0.000**	0.830 ± 0.003
0.70	k-Nearest Neighbours	0.821 ± 0.004	0.890 ± 0.004	0.915 ± 0.003	0.824 ± 0.004	0.822 ± 0.004	0.828 ± 0.004	0.823 ± 0.004	0.996 ± 0.000	0.823 ± 0.004
1.00	Logistic Regression	0.833 ± 0.003	**0.911 ± 0.003**	**0.941 ± 0.002**	0.835 ± 0.003	0.834 ± 0.003	0.836 ± 0.003	0.834 ± 0.003	0.997 ± 0.000	0.834 ± 0.003
1.00	k-Nearest Neighbours	**0.834 ± 0.003**	0.899 ± 0.003	0.922 ± 0.002	**0.836 ± 0.003**	**0.835 ± 0.003**	**0.840 ± 0.003**	**0.836 ± 0.003**	0.997 ± 0.000	**0.836 ± 0.003**

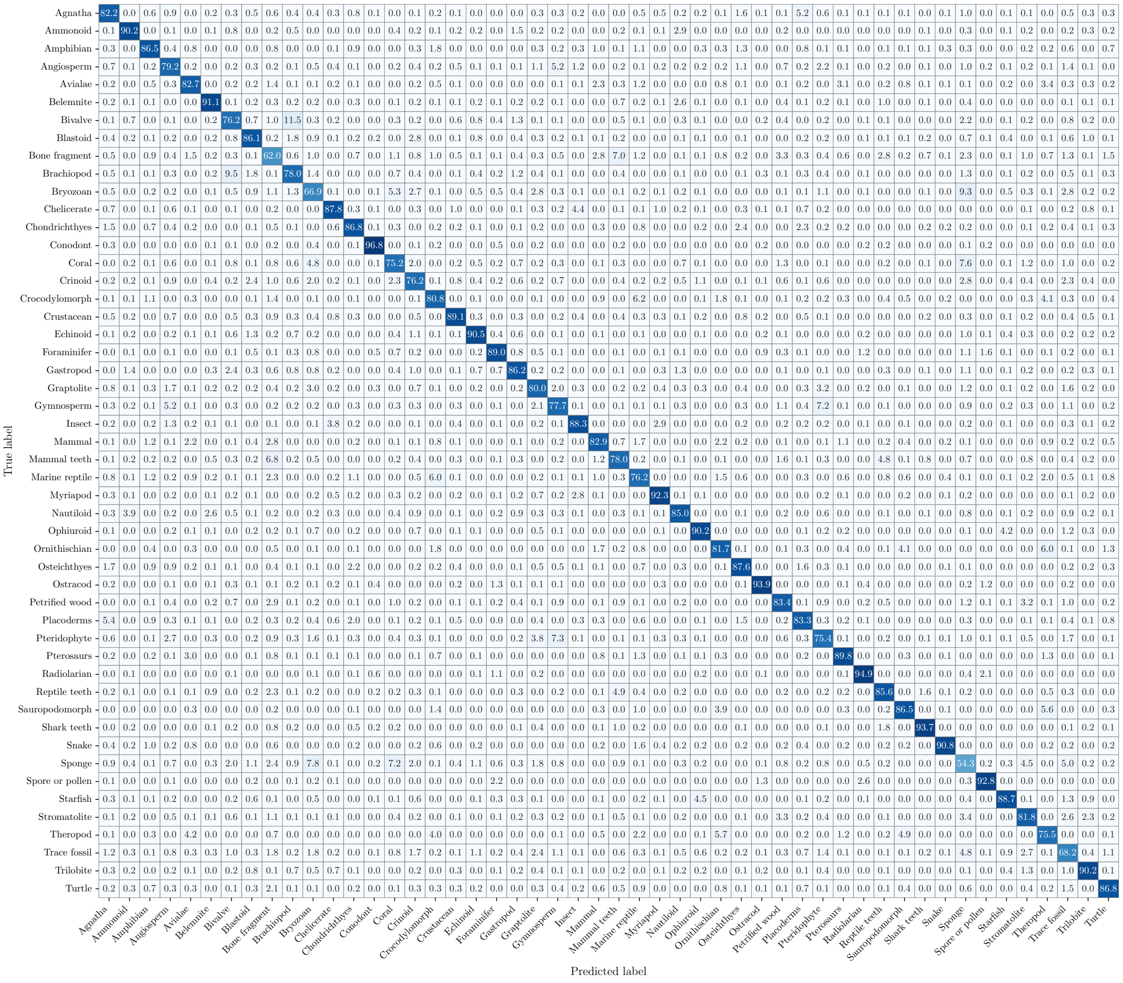

Figure S7: Confusion Matrix - RFID

Table S17: Few-Shot Classification Results - RFID

Few-Shot k	Classifier	Repeats Successful	Top-1 Accuracy	Top-2 Accuracy	Top-3 Accuracy	F1 Macro	F1 Weighted	Precision Macro	Recall Macro	Specificity Macro	Balanced Accuracy
1	k-Nearest Neighbours	50	0.343 ± 0.028	0.357 ± 0.027	0.376 ± 0.027	0.333 ± 0.028	0.334 ± 0.028	0.401 ± 0.033	0.342 ± 0.028	0.987 ± 0.001	0.342 ± 0.028
1	Logistic Regression	50	0.355 ± 0.025	0.473 ± 0.028	0.544 ± 0.028	0.346 ± 0.025	0.346 ± 0.025	0.393 ± 0.027	0.355 ± 0.024	0.987 ± 0.001	0.355 ± 0.024
2	k-Nearest Neighbours	50	0.345 ± 0.019	0.537 ± 0.019	0.549 ± 0.018	0.321 ± 0.023	0.323 ± 0.022	0.401 ± 0.025	0.336 ± 0.022	0.987 ± 0.000	0.336 ± 0.022
2	Logistic Regression	50	0.461 ± 0.018	0.591 ± 0.017	0.663 ± 0.017	0.455 ± 0.020	0.456 ± 0.020	0.486 ± 0.022	0.461 ± 0.018	0.989 ± 0.000	0.461 ± 0.018
3	k-Nearest Neighbours	50	0.390 ± 0.018	0.557 ± 0.017	0.652 ± 0.017	0.381 ± 0.019	0.381 ± 0.019	0.476 ± 0.022	0.389 ± 0.018	0.988 ± 0.000	0.389 ± 0.018
3	Logistic Regression	50	0.520 ± 0.015	0.653 ± 0.015	0.720 ± 0.014	0.518 ± 0.016	0.517 ± 0.016	0.542 ± 0.016	0.521 ± 0.015	0.990 ± 0.000	0.521 ± 0.015
5	k-Nearest Neighbours	50	0.458 ± 0.012	0.601 ± 0.012	0.685 ± 0.012	0.456 ± 0.013	0.456 ± 0.013	0.522 ± 0.013	0.461 ± 0.012	0.989 ± 0.000	0.461 ± 0.012
5	Logistic Regression	50	0.587 ± 0.013	0.717 ± 0.011	0.780 ± 0.010	0.587 ± 0.014	0.586 ± 0.014	0.603 ± 0.015	0.588 ± 0.013	0.992 ± 0.000	0.588 ± 0.013
10	k-Nearest Neighbours	50	0.548 ± 0.009	0.681 ± 0.008	0.750 ± 0.008	0.553 ± 0.009	0.552 ± 0.009	0.594 ± 0.010	0.554 ± 0.009	0.991 ± 0.000	0.554 ± 0.009
10	Logistic Regression	50	0.663 ± 0.006	0.782 ± 0.005	0.838 ± 0.005	0.665 ± 0.006	0.664 ± 0.006	0.674 ± 0.007	0.664 ± 0.006	0.993 ± 0.000	0.664 ± 0.006
20	k-Nearest Neighbours	50	0.622 ± 0.007	0.740 ± 0.005	0.797 ± 0.005	0.628 ± 0.007	0.626 ± 0.007	0.652 ± 0.006	0.626 ± 0.007	0.992 ± 0.000	0.626 ± 0.007
20	Logistic Regression	50	**0.717 ± 0.005**	**0.826 ± 0.004**	**0.873 ± 0.004**	**0.719 ± 0.005**	**0.718 ± 0.005**	**0.726 ± 0.006**	**0.718 ± 0.005**	**0.994 ± 0.000**	**0.718 ± 0.005**

Table S18: Clustering results - RFID

Pipeline	Algorithm	No. of Clusters	ARI	AMI	Purity	Silhouette	DBI	Noise Fraction	Components Used
PCA	KMeans (Known K)	50	0.3148	0.5708	0.4789	0.1649	2.0605	0.000000	50
PCA	KMeans (Unknown K)	39	0.2777	0.5543	0.4198	0.1693	1.9669	0.000000	50
PCA	Bayesian GMM	50	0.3092	0.5910	0.4713	0.1262	2.2057	0.000000	50
PCA	HDBSCAN	0	0.0000	0.0000	0.0000	nan	nan	1.000000	50
Exploratory UMAP	KMeans (Known K)	50	**0.3571**	0.6390	0.5051	0.5636	0.5652	0.000000	15
Exploratory UMAP	KMeans (Unknown K)	37	0.3195	0.6245	0.4353	**0.5847**	0.5169	0.000000	15
Exploratory UMAP	Bayesian GMM	50	0.3497	**0.6440**	**0.5057**	0.5197	0.9864	0.000000	15
Exploratory UMAP	HDBSCAN	7	0.0684	0.4328	0.1355	0.5587	**0.4859**	0.093309	15

S1.11 Larger Images from the Main Text

In this section, we repeat the dimension reduction figures given in the main text but giving each figure its own page so that details can be observed more clearly and so that the correspondence between colours and labels can be clearly read off from the figure legends.

S1.11.1 Dimension Reduction with True Labels from Main Text

Non-linear dimensionality reduction methods applied to feature vectors for all images across all datasets with points labelled according to their true class.

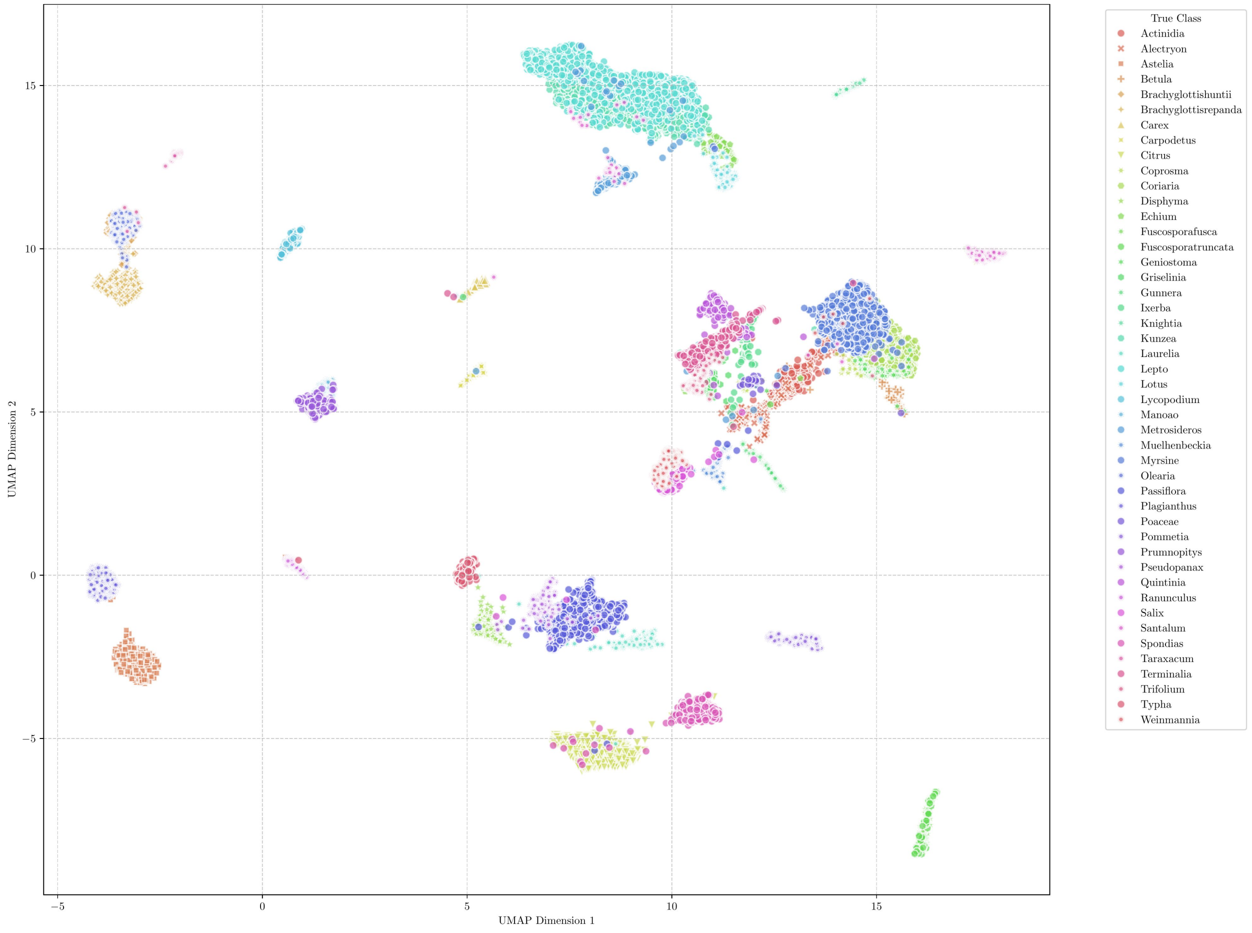

Figure S8: Pollen - UMAP

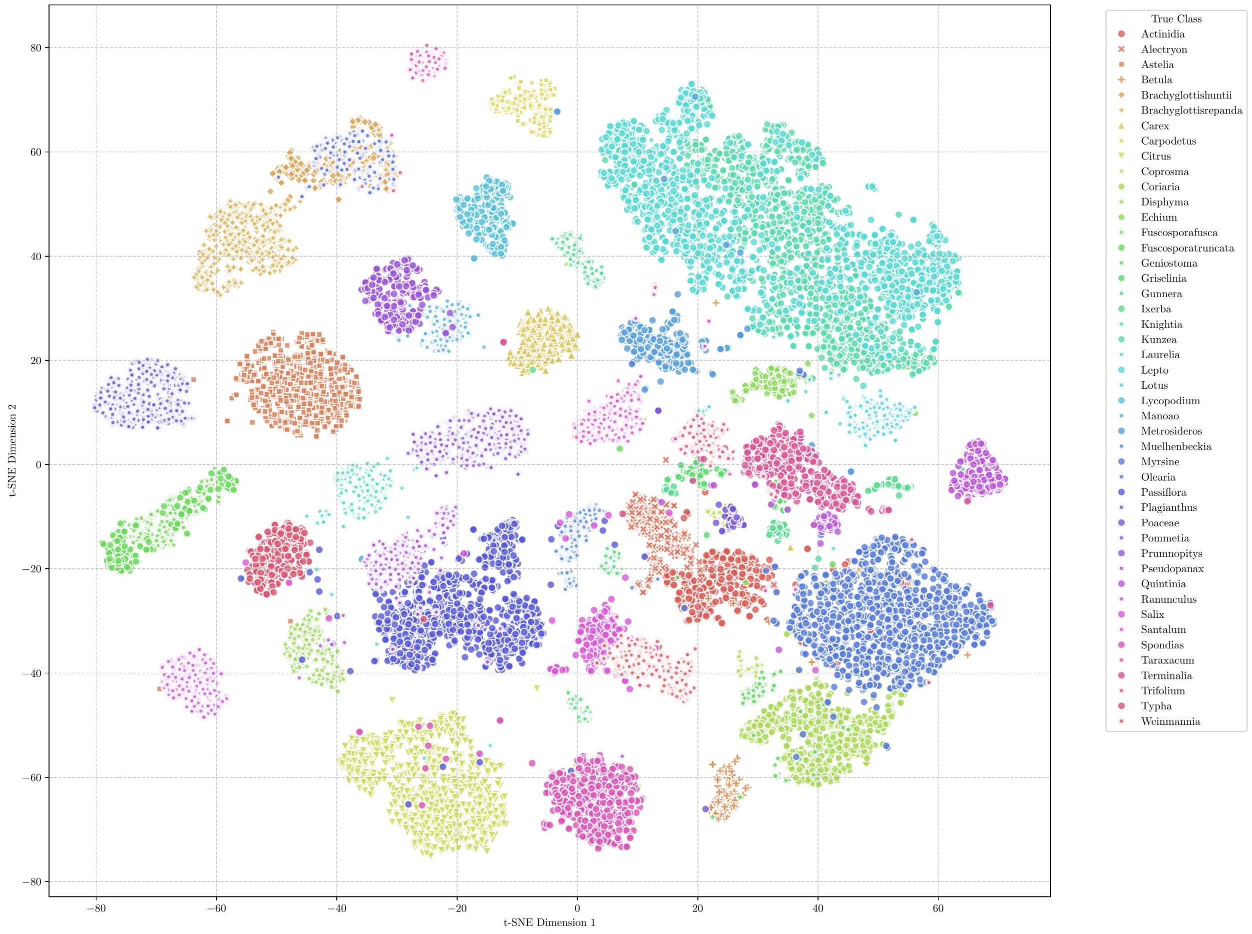

Figure S9: Pollen - t-SNE

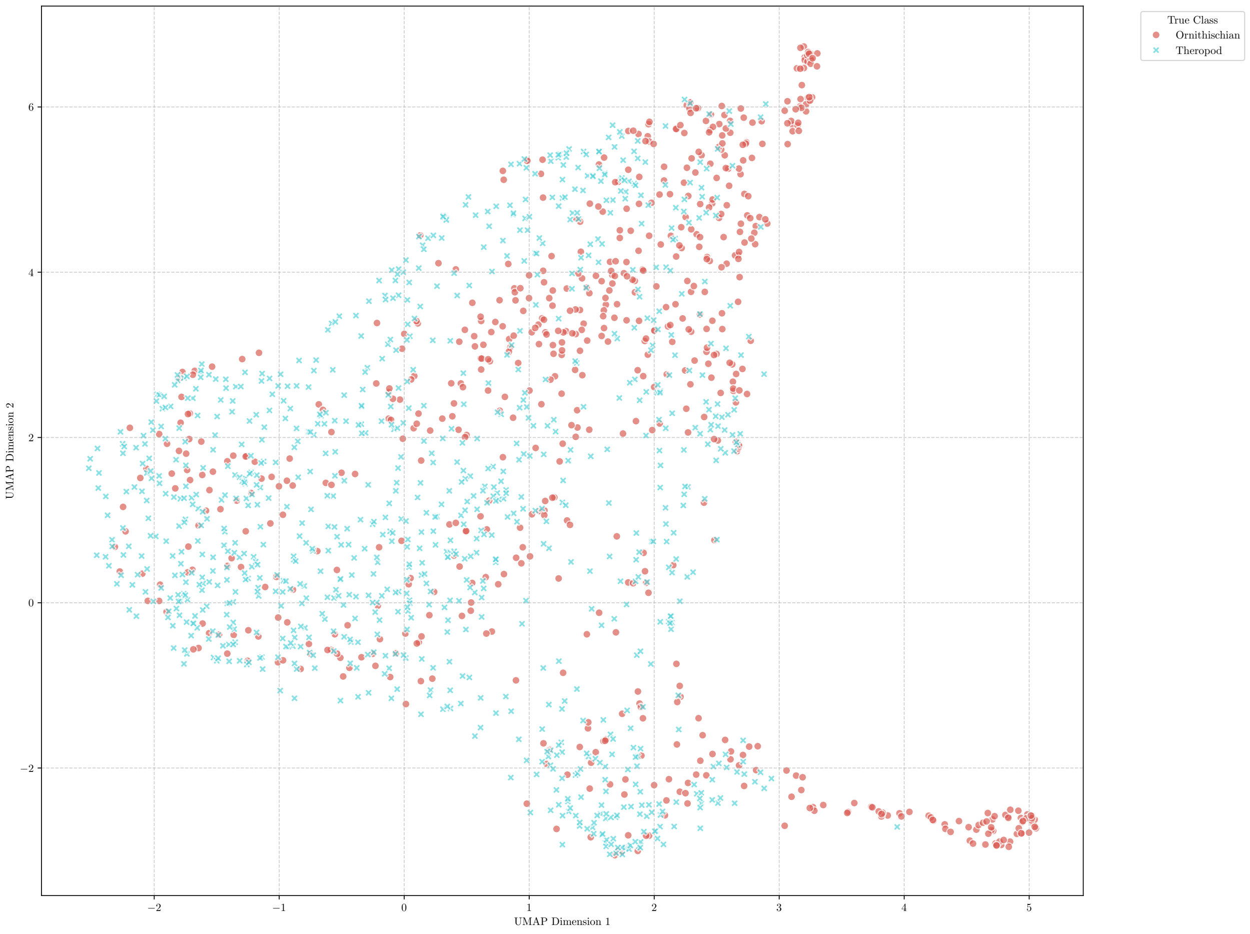

Figure S10: Fossil Tracks - UMAP

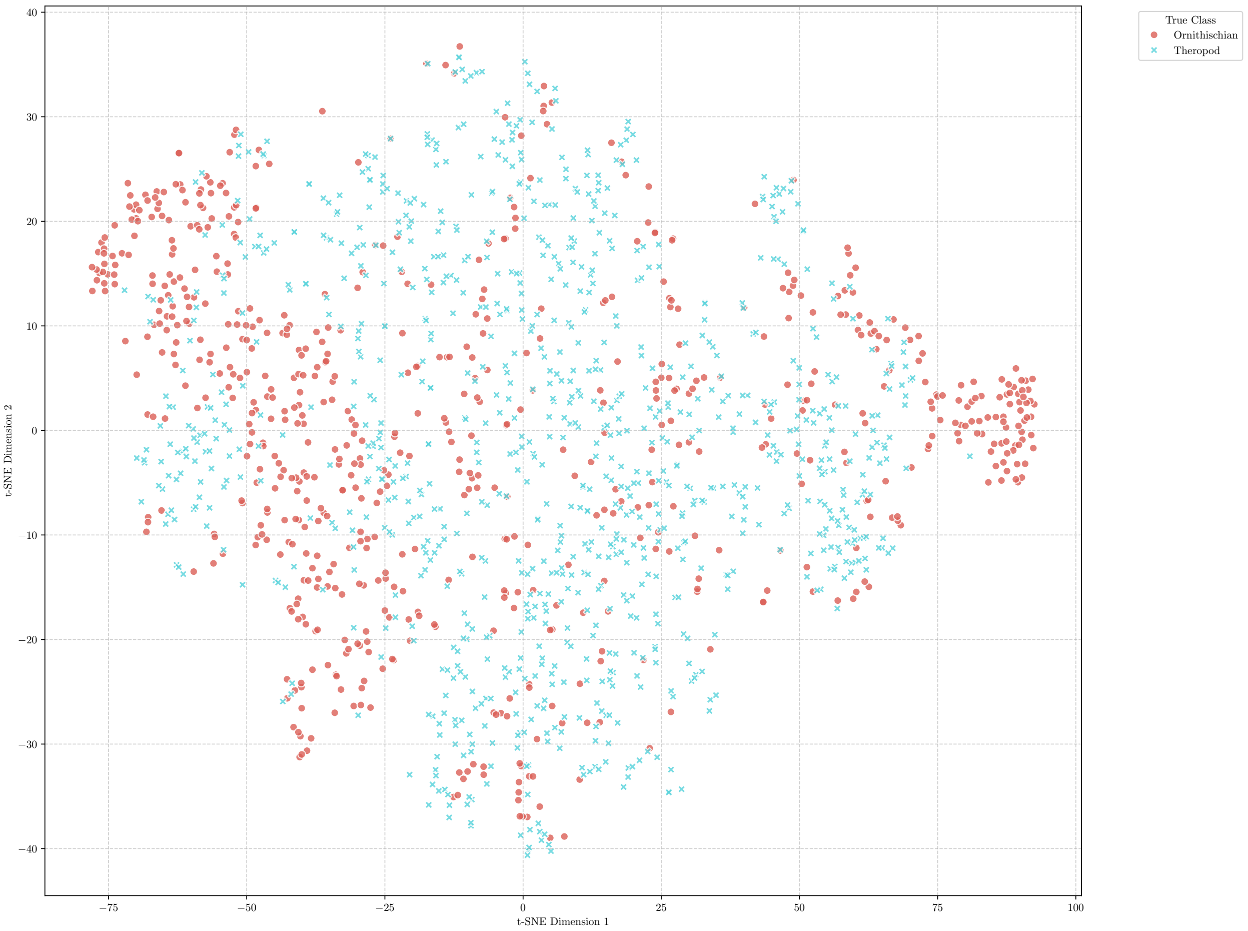

Figure S11: Fossil Tracks - t-SNE

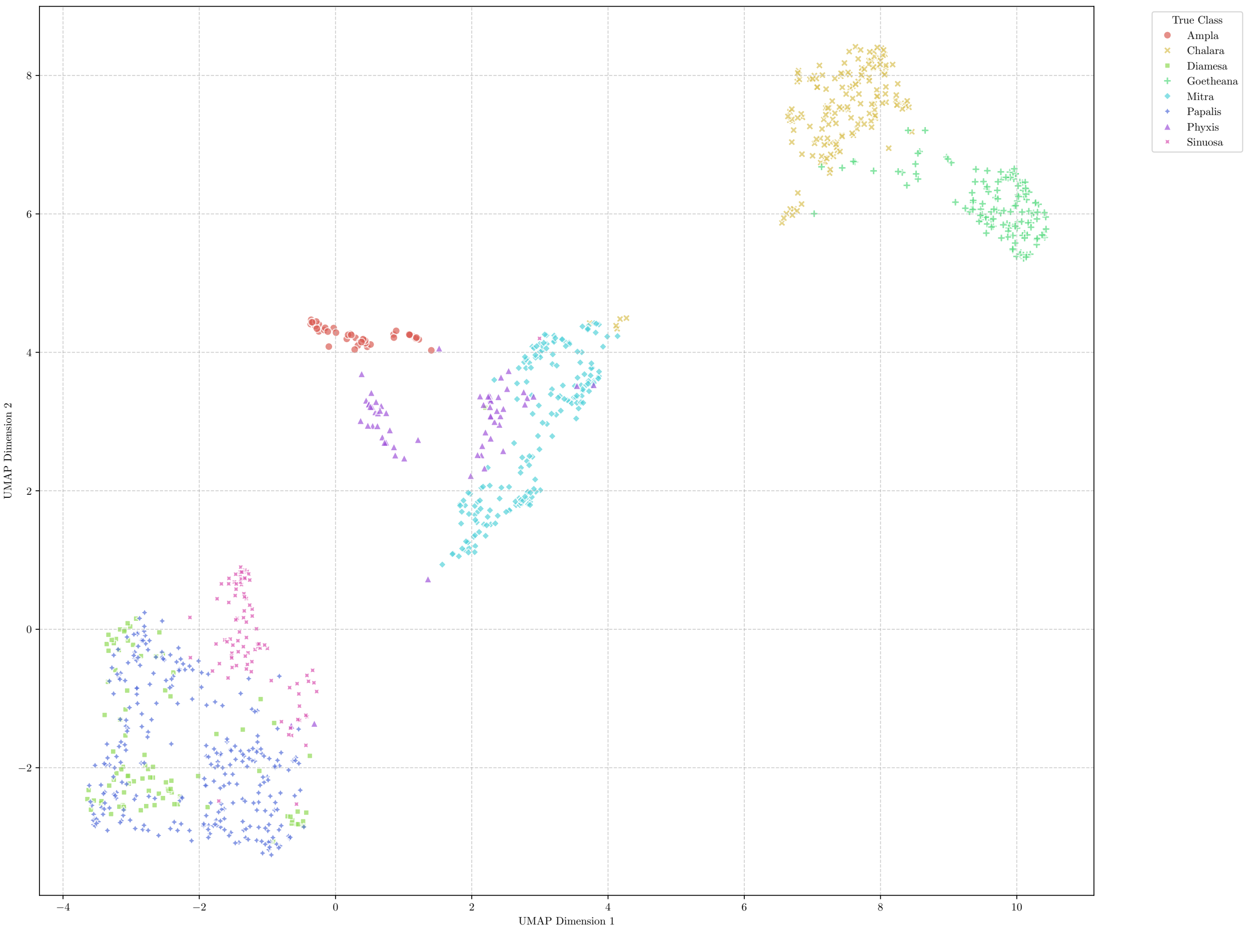

Figure S12: Radiolaria - UMAP

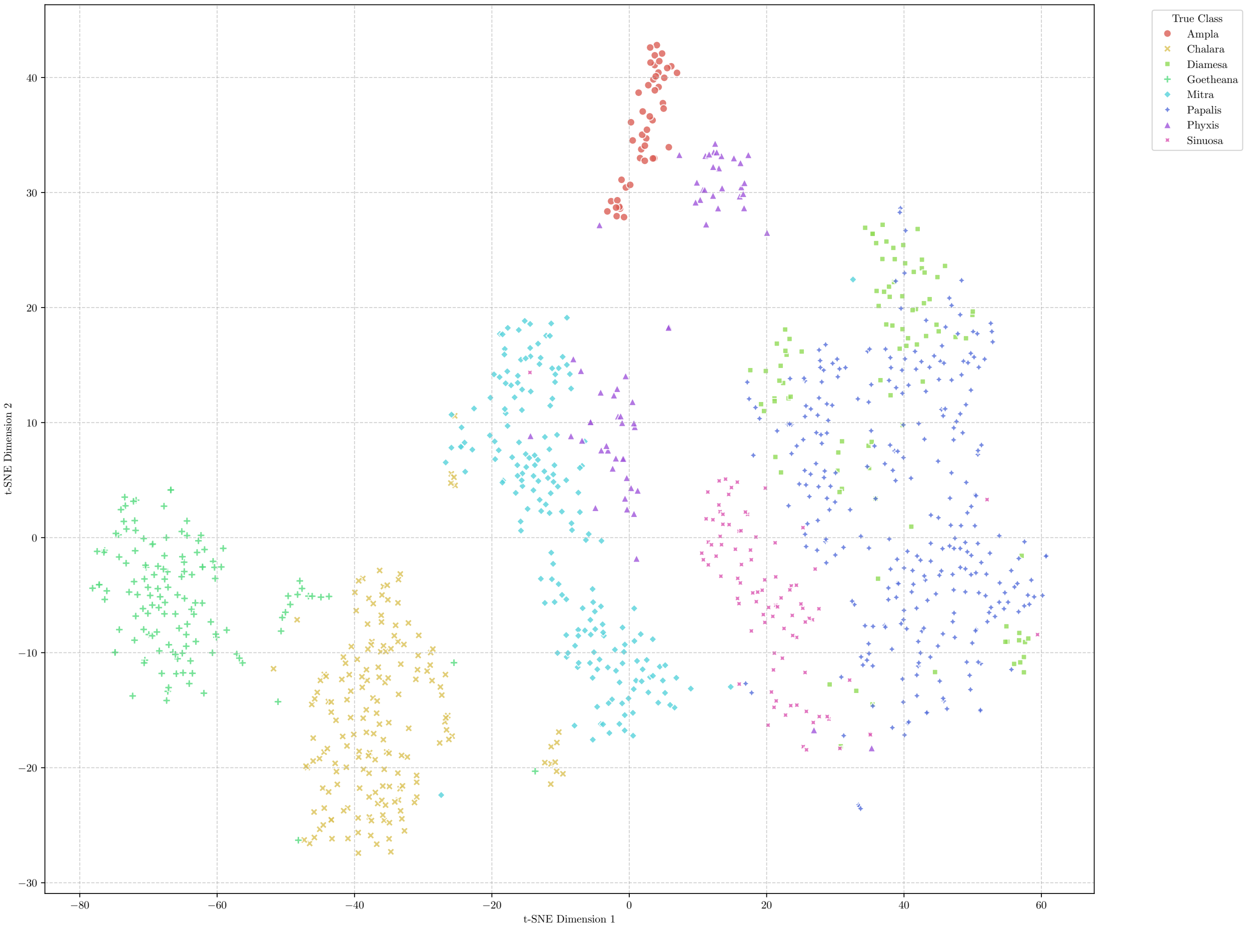

Figure S13: Radiolaria - t-SNE

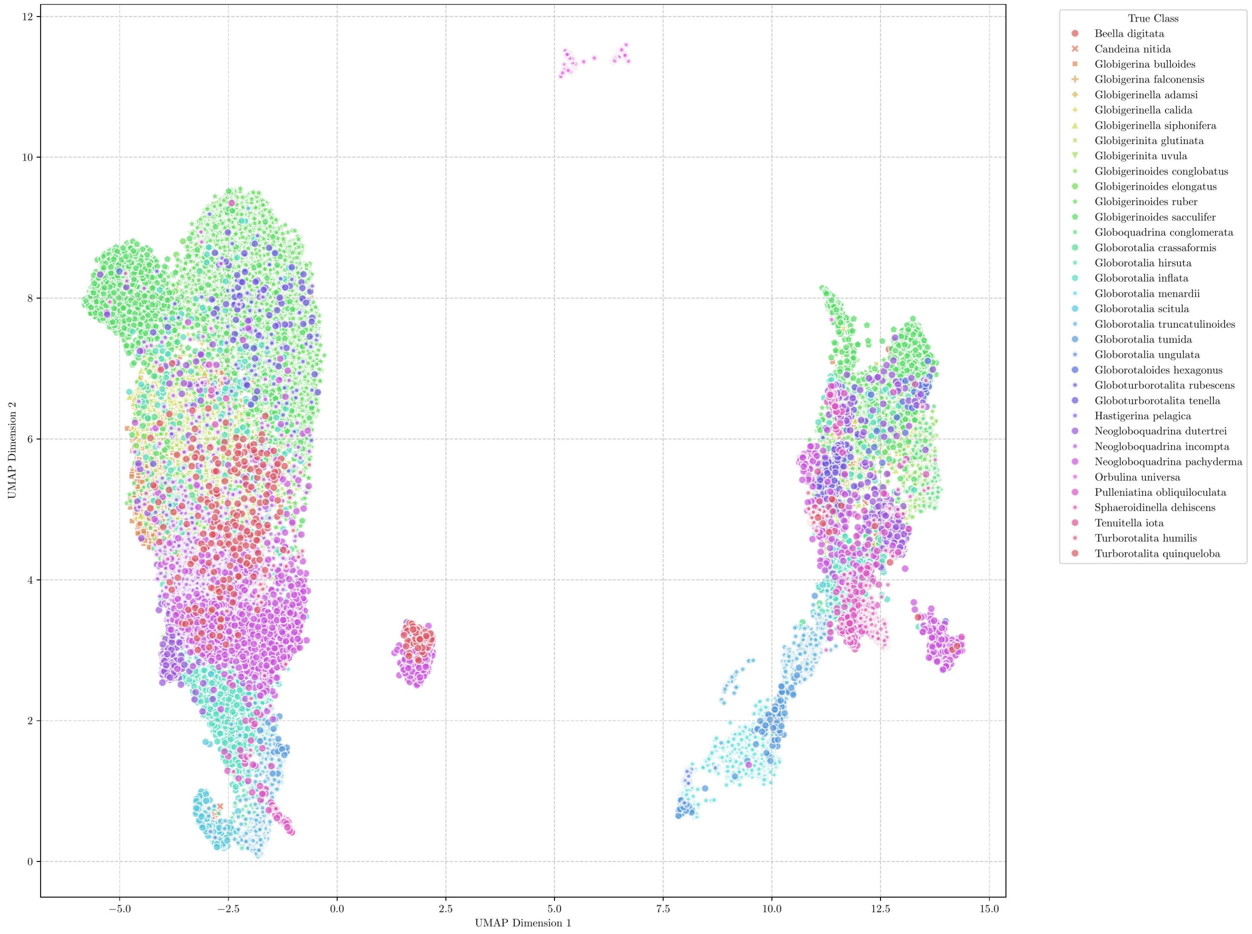

Figure S14: Foraminifera - UMAP

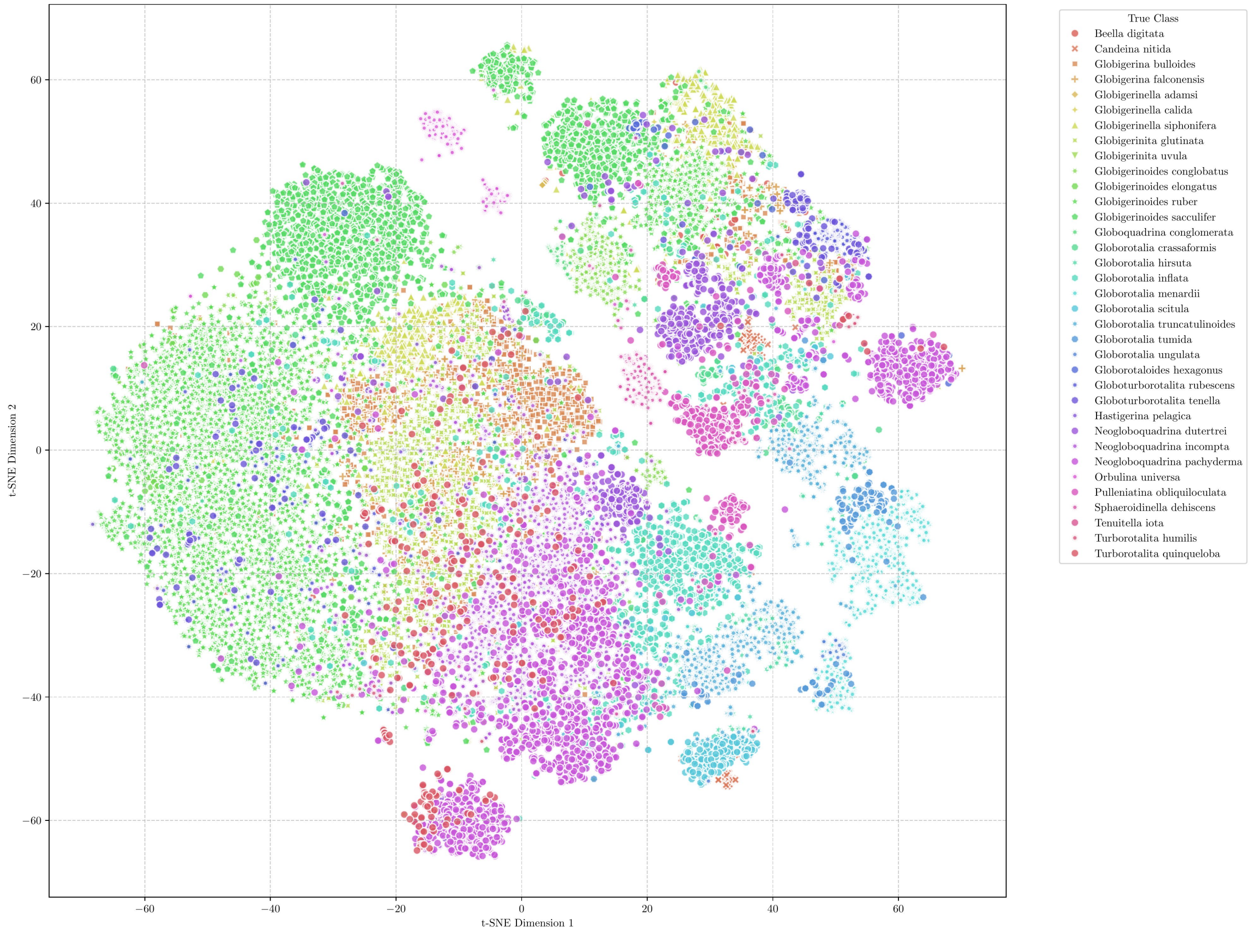

Figure S15: Foraminifera - t-SNE

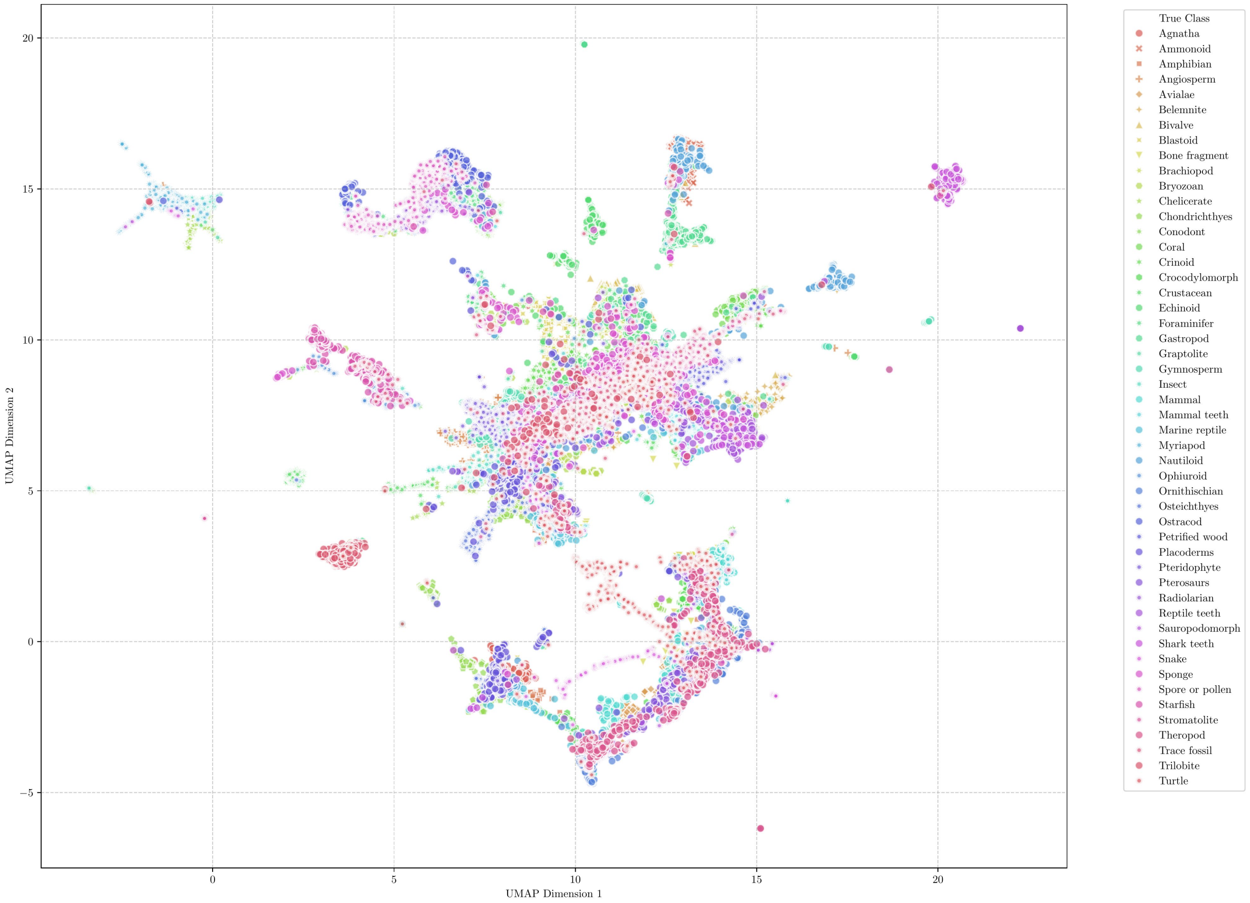

Figure S16: Diverse Palaeontological images (RFID) - UMAP

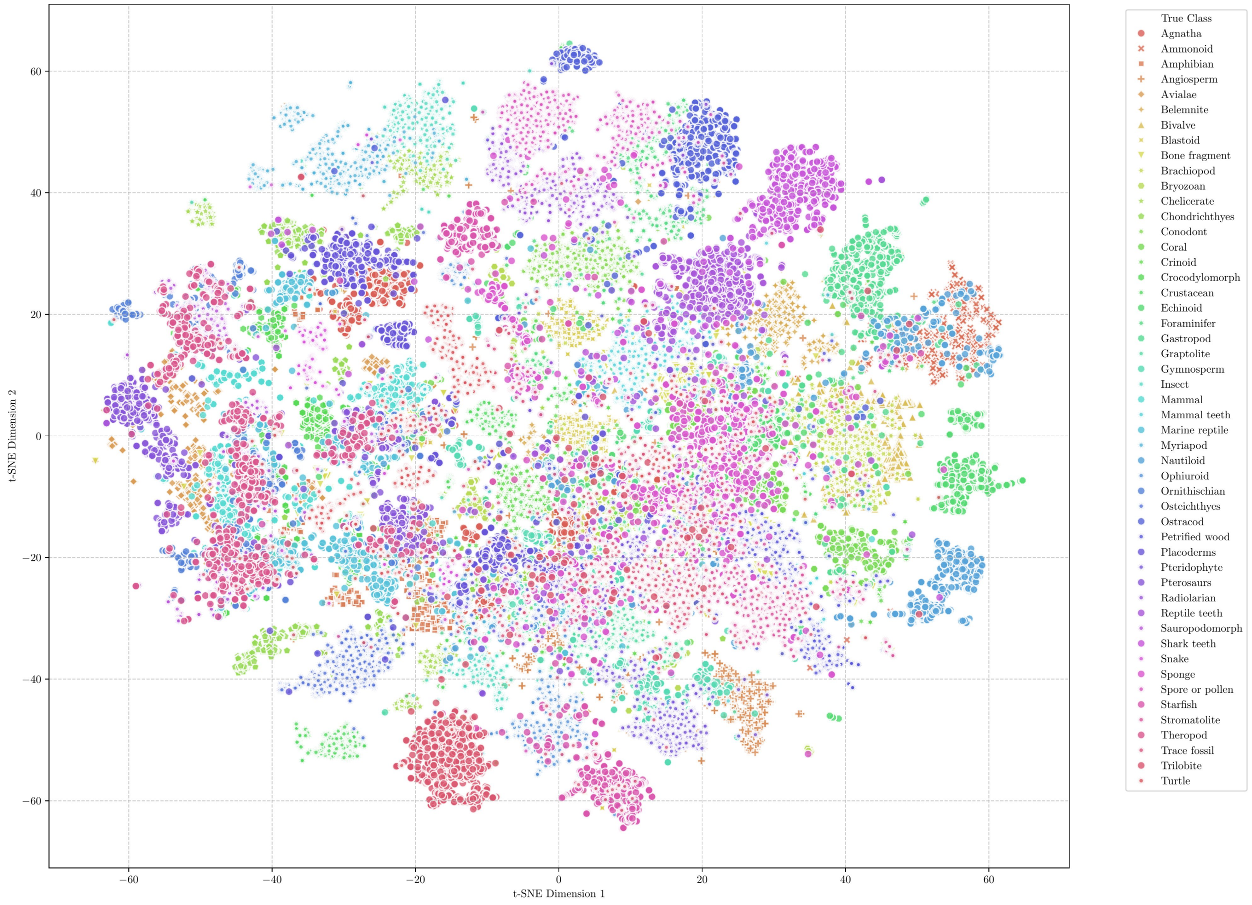

Figure S17: Diverse Palaeontological images (RFID) - t-SNE

S1.11.2 Clustering Plots from Main Text

In this section, we replot the clustering results described in the main text, in a larger format.

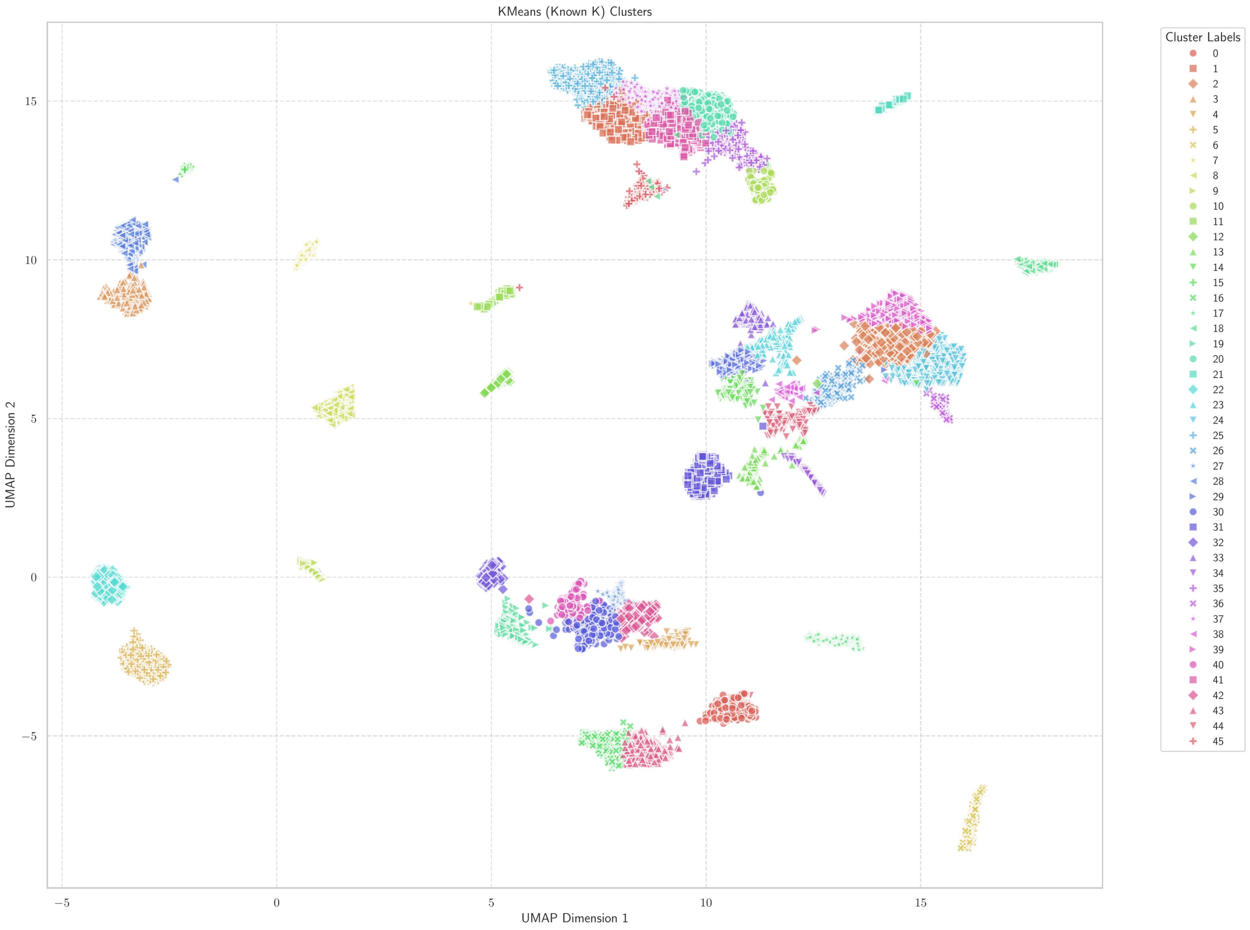

Figure S18: Pollen - (known k) k-Means

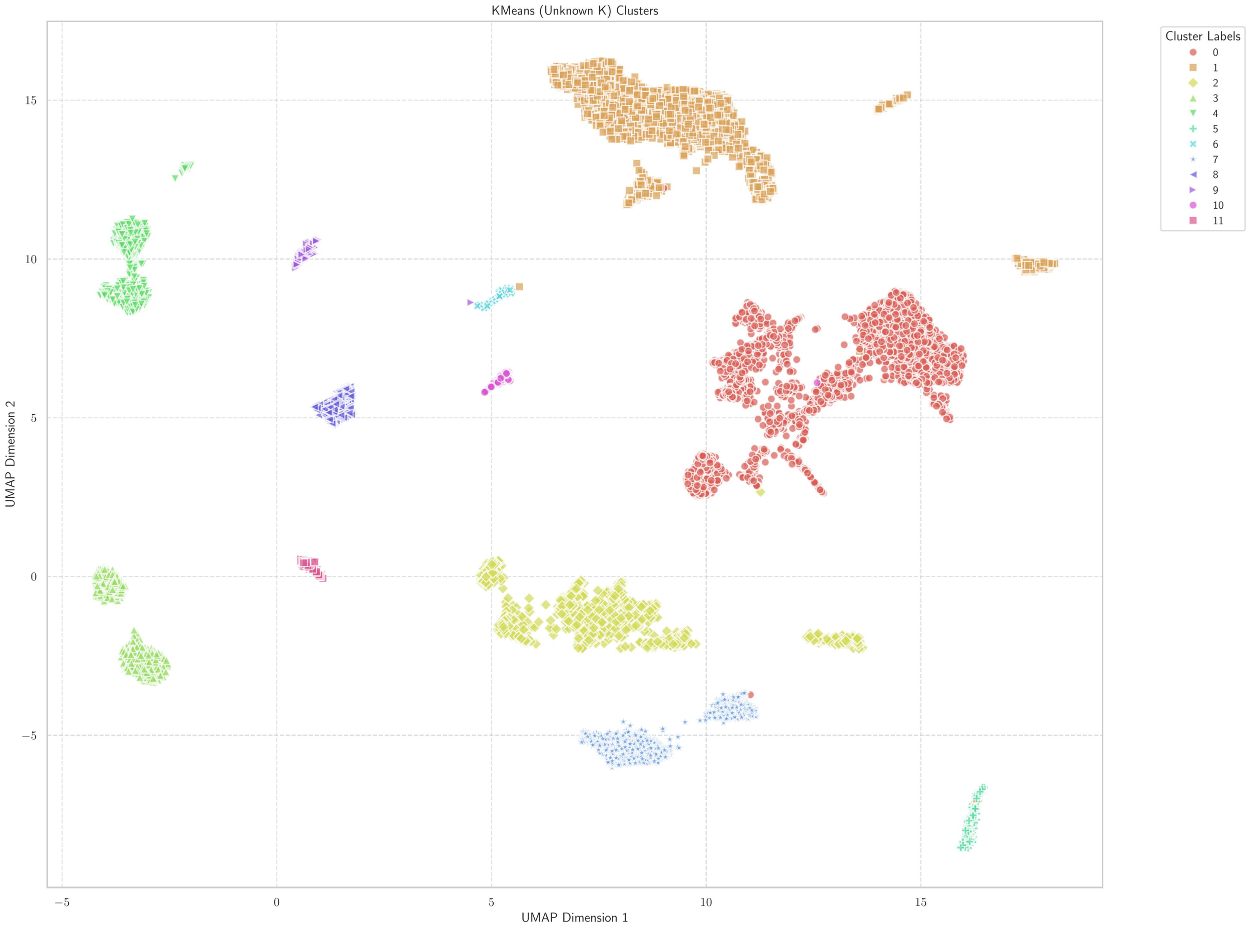

Figure S19: Pollen - (unknown k) k-Means

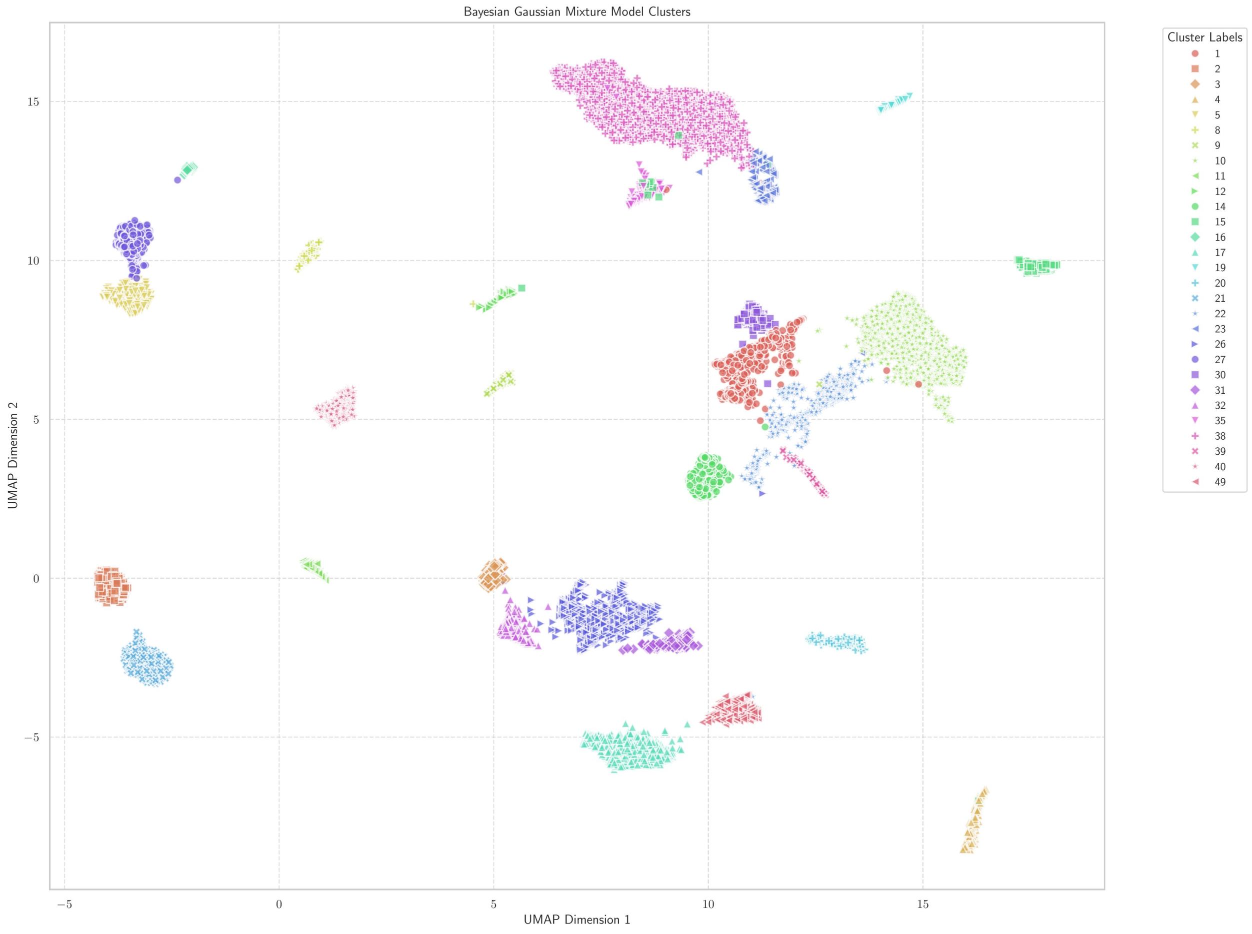

Figure S20: Pollen - BGMM

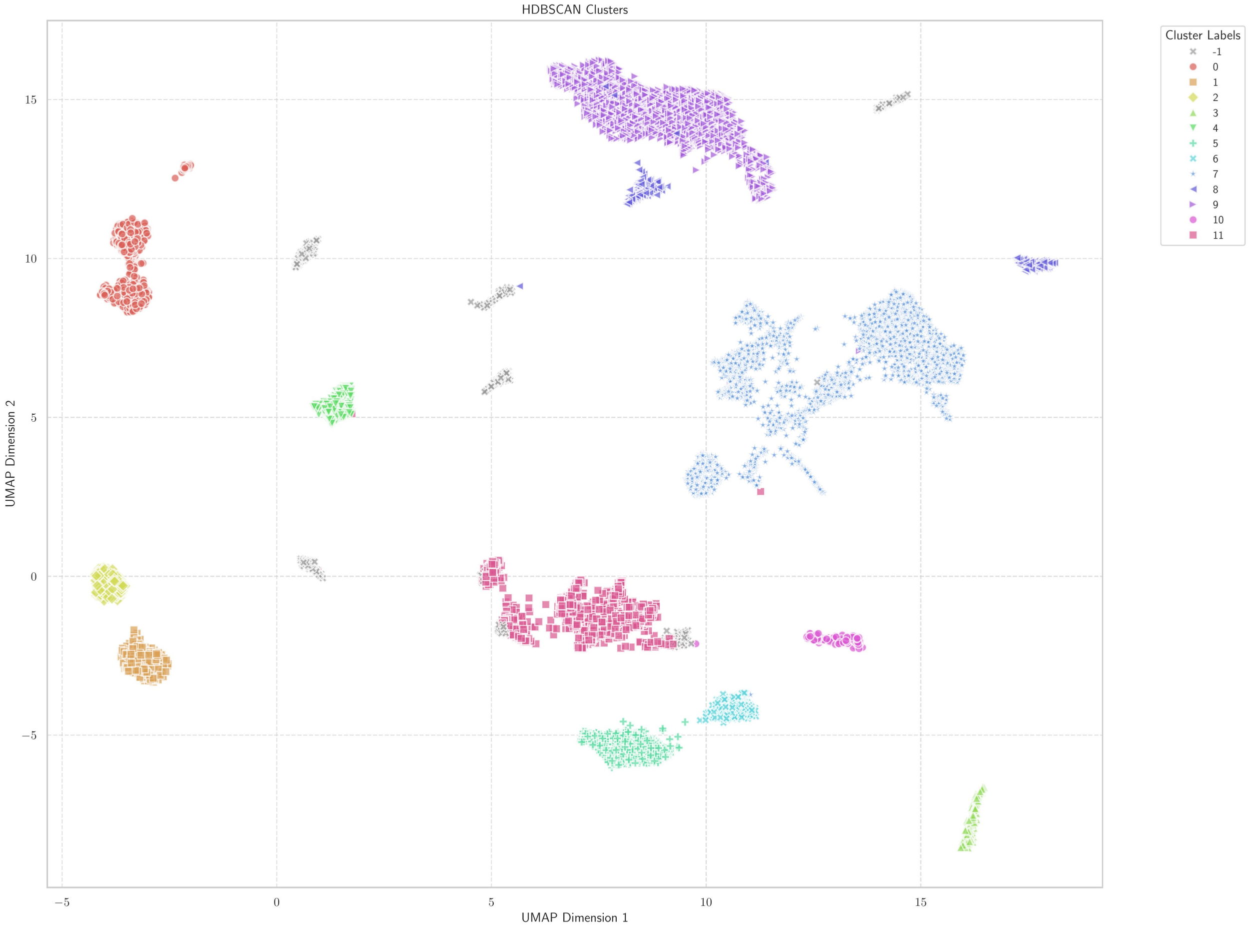

Figure S21: Pollen - HDBSCAN

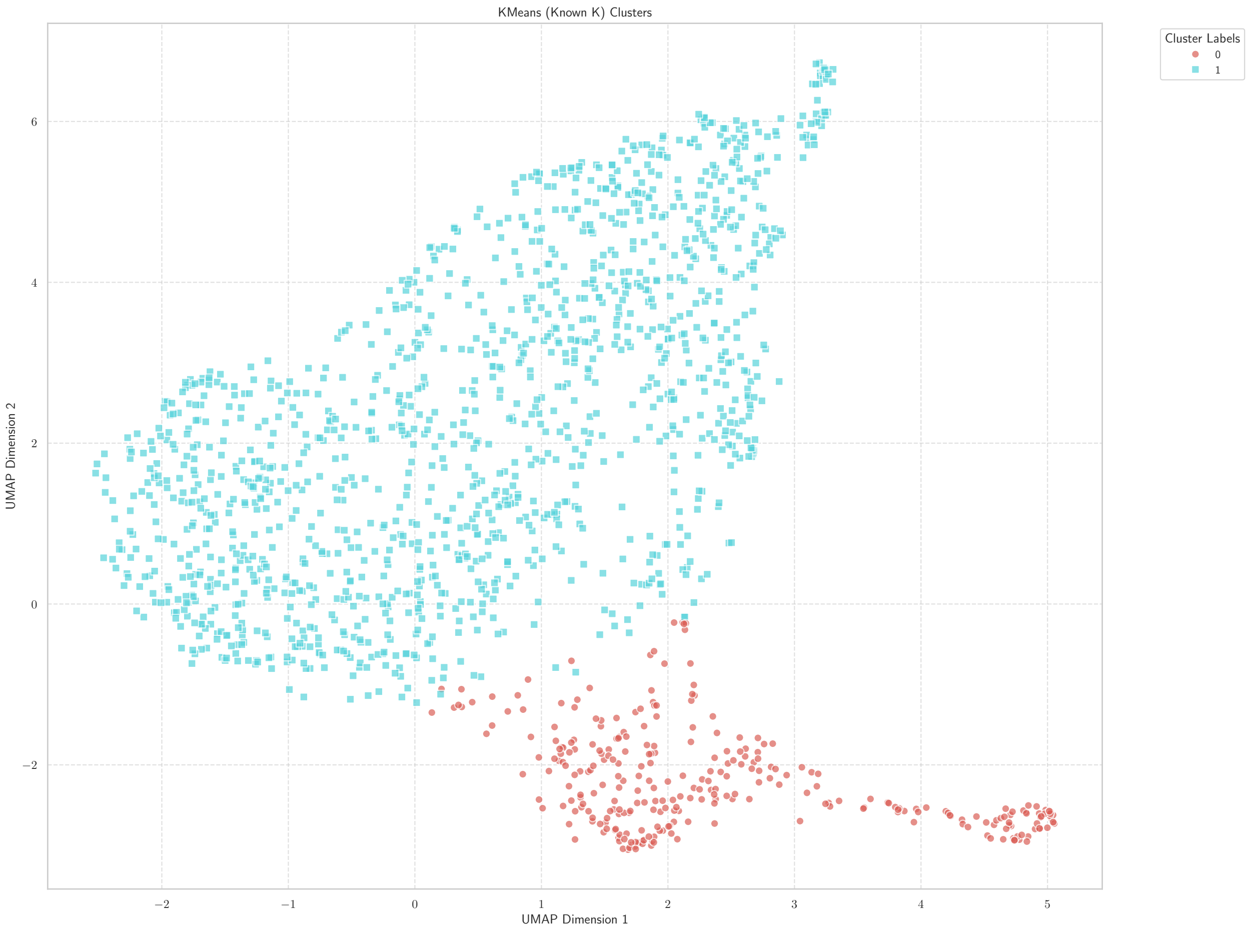

Figure S22: Fossil Tracks - (known k) k-Means

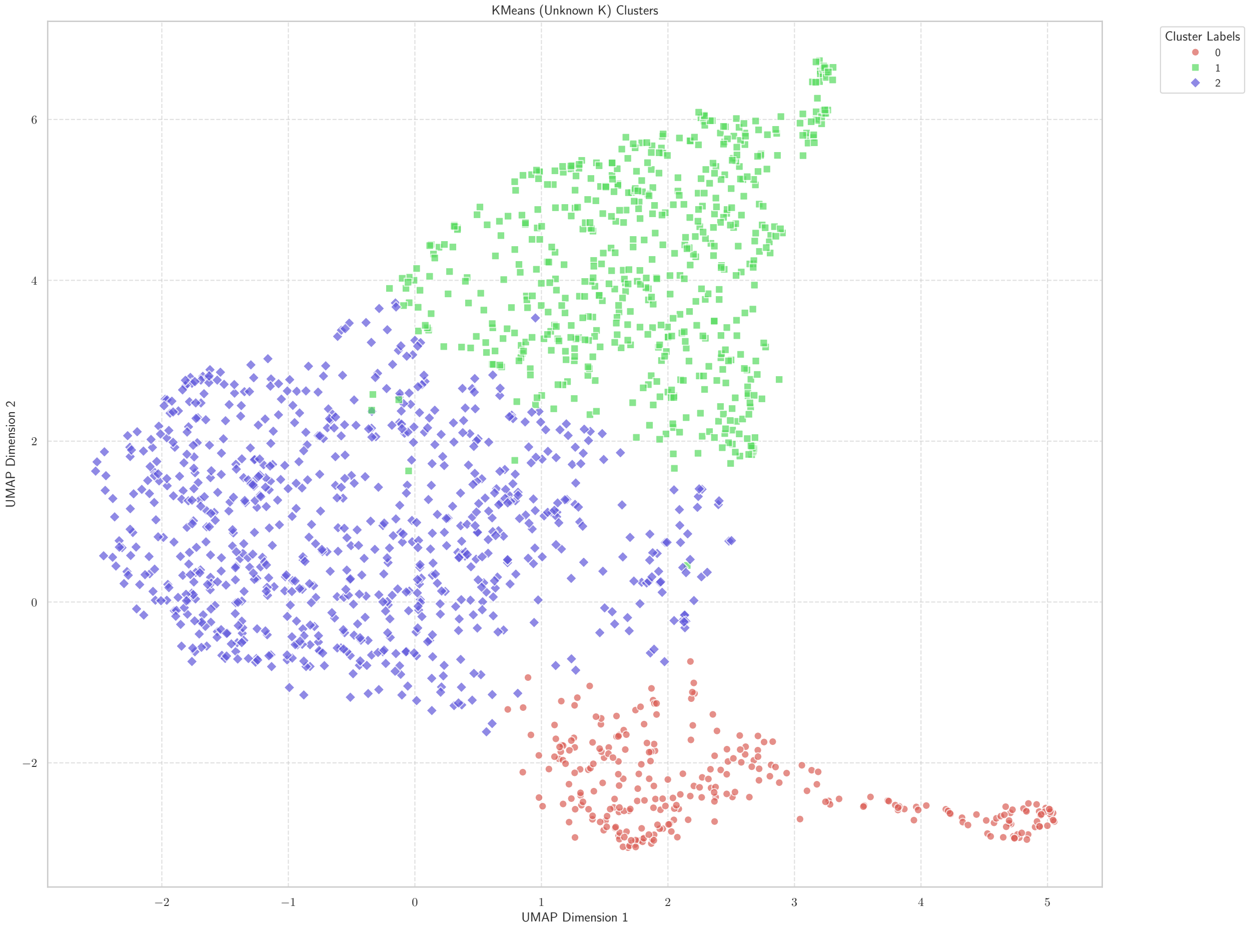

Figure S23: Fossil Tracks - (unknown k) k-Means

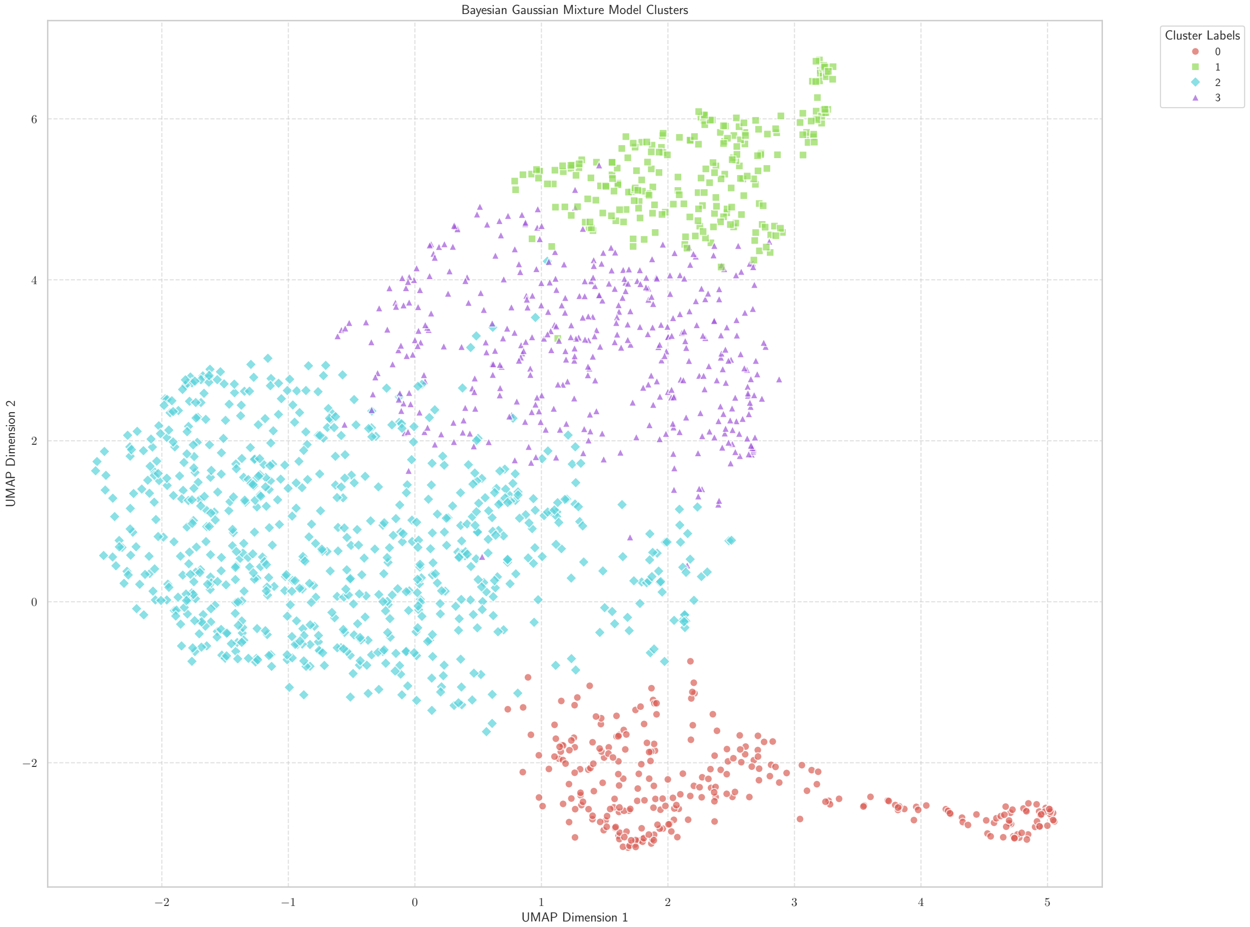

Figure S24: Fossil Tracks - BGMM

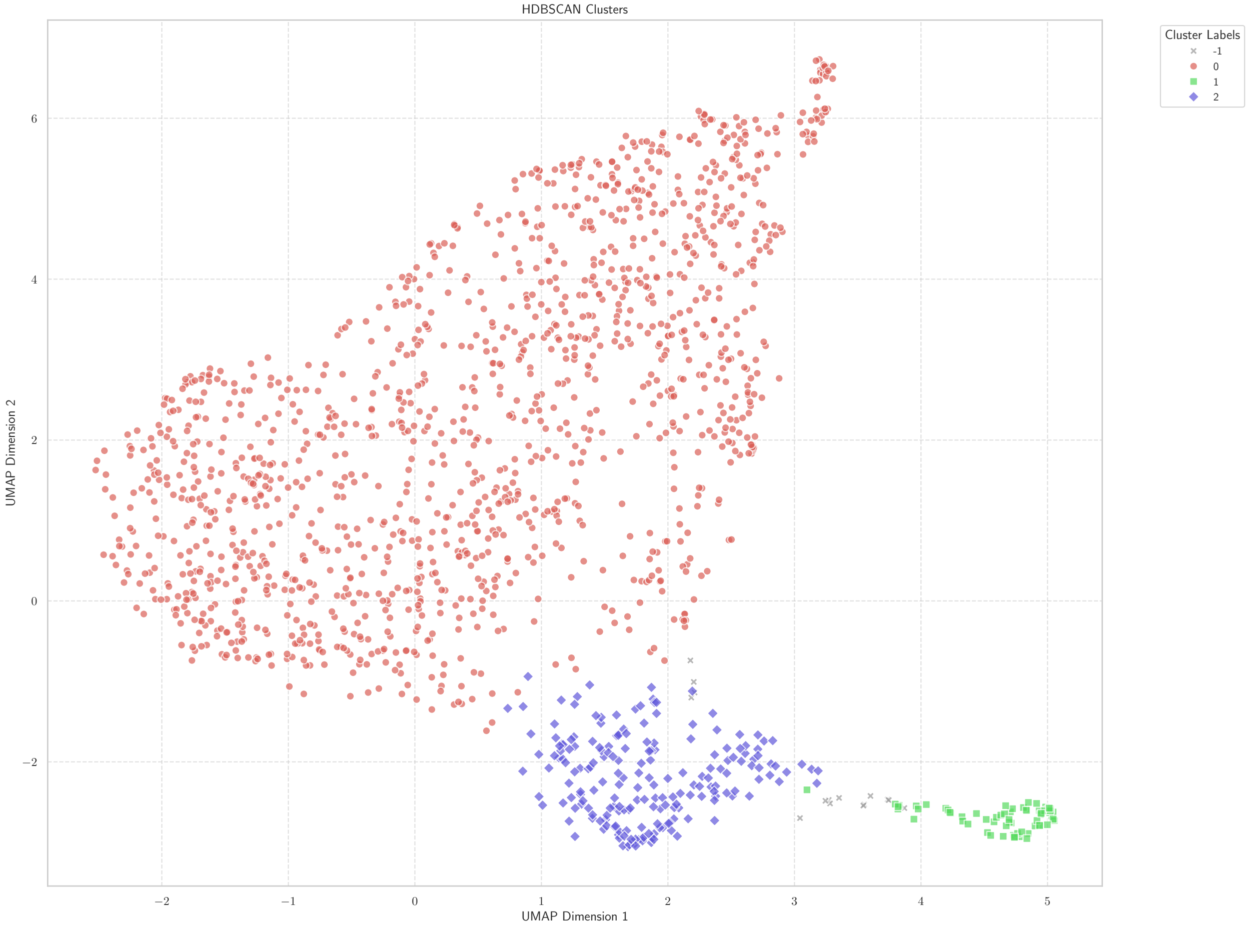

Figure S25: Fossil Tracks - HDBSCAN

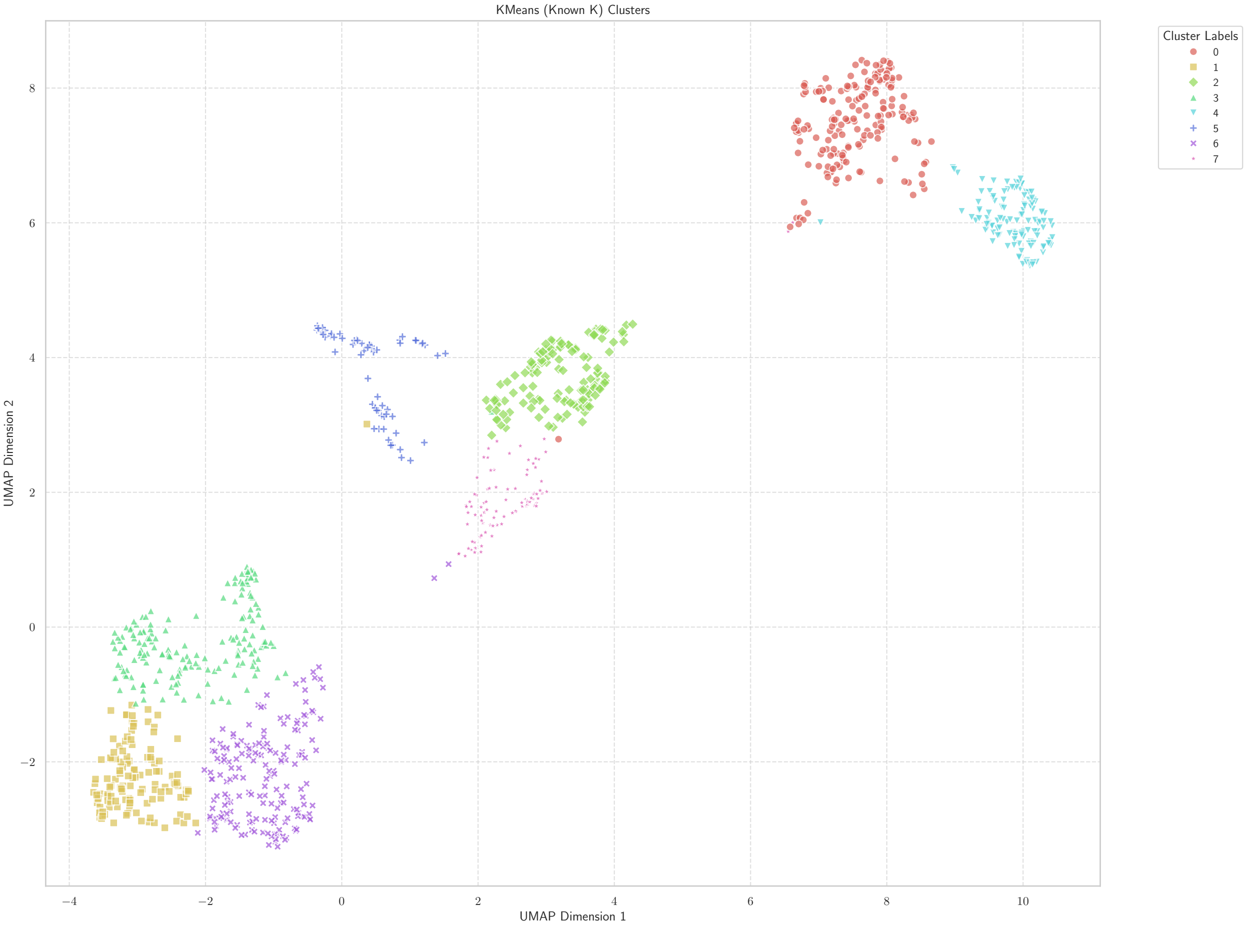

Figure S26: Radiolaria - (known k) k-Means

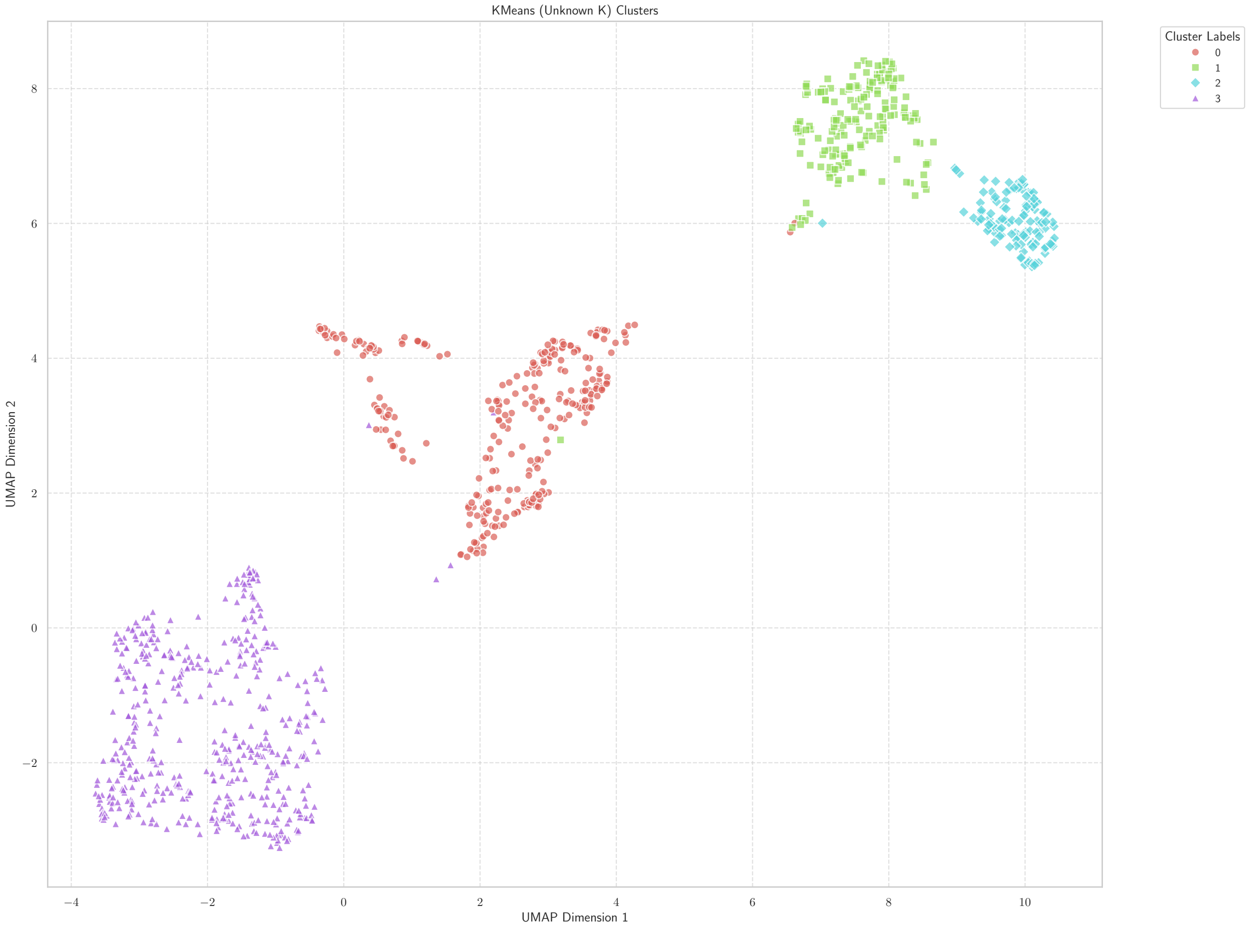

Figure S27: Radiolaria - (unknown k) k-Means

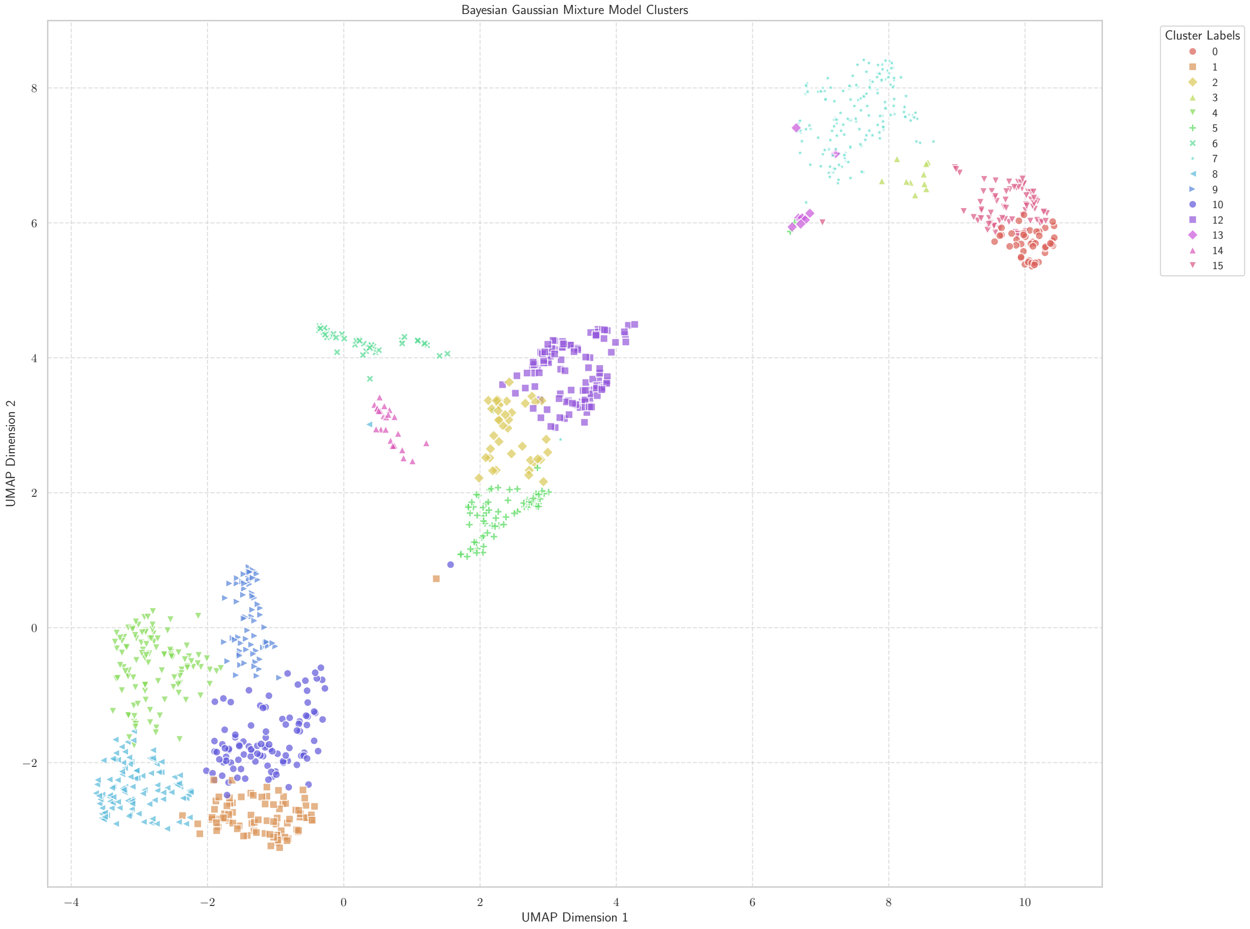

Figure S28: Radiolaria - BGMM

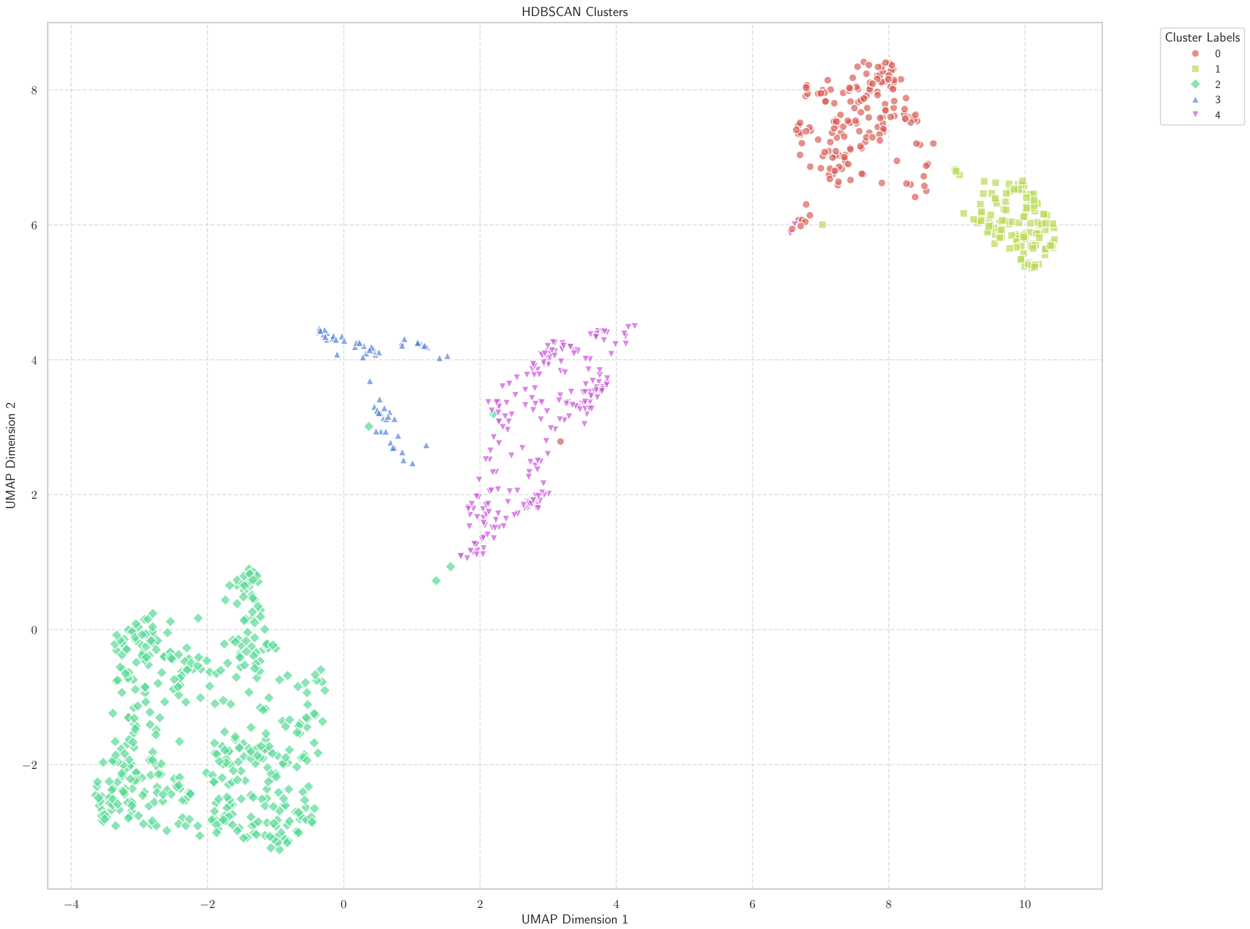

Figure S29: Radiolaria - HDBSCAN

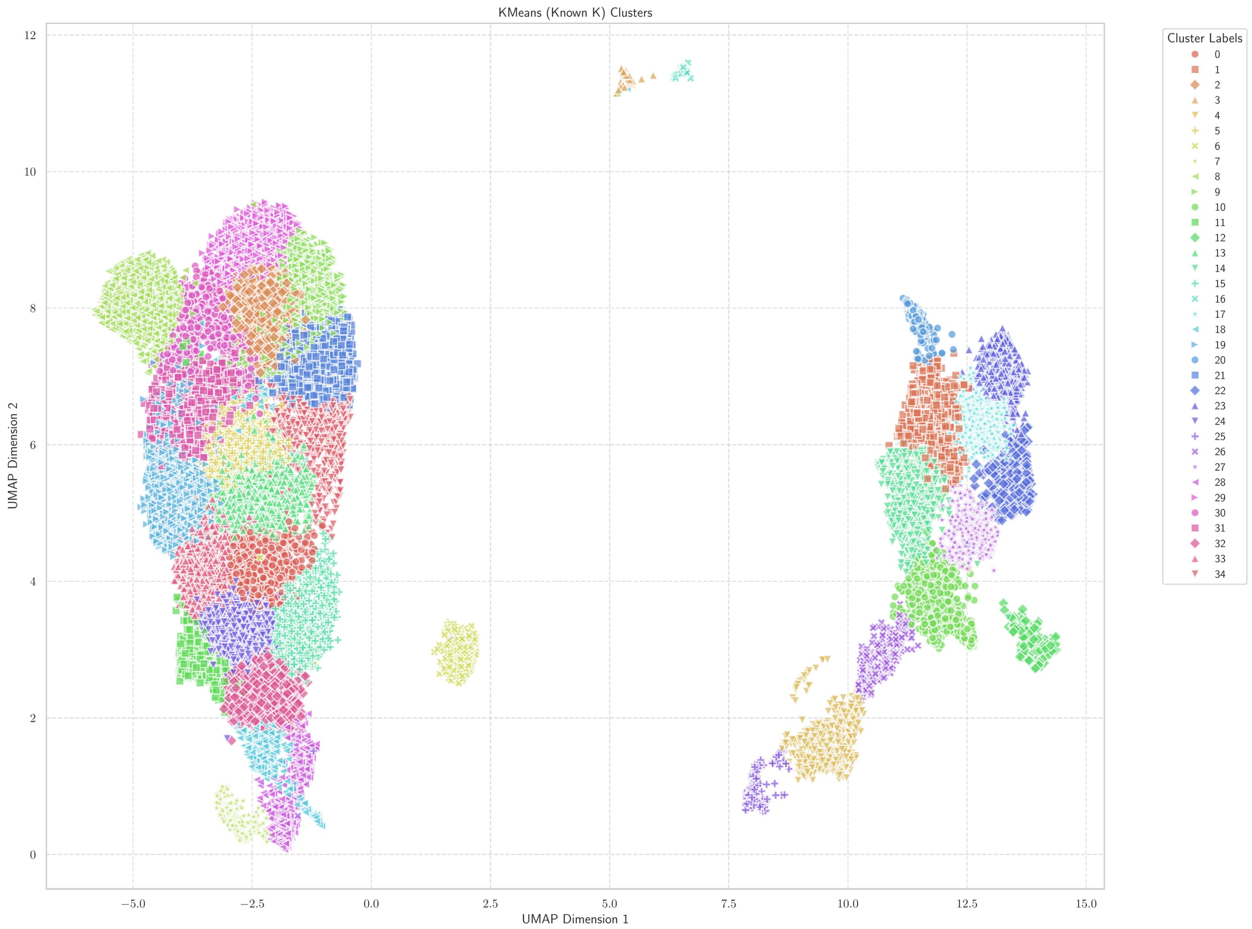

Figure S30: Foraminifera - (known k) k-Means

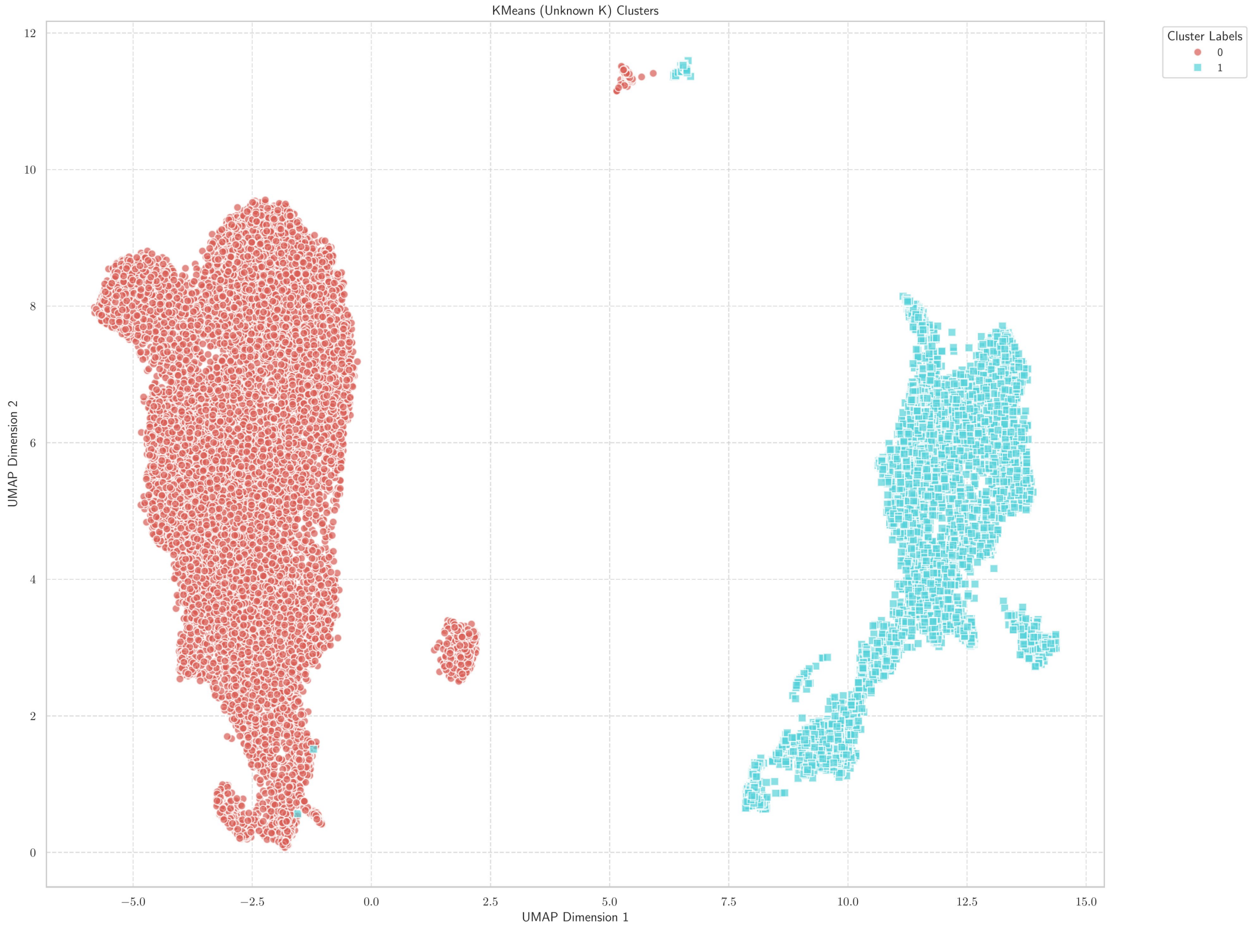

Figure S31: Foraminifera - (unknown k) k-Means

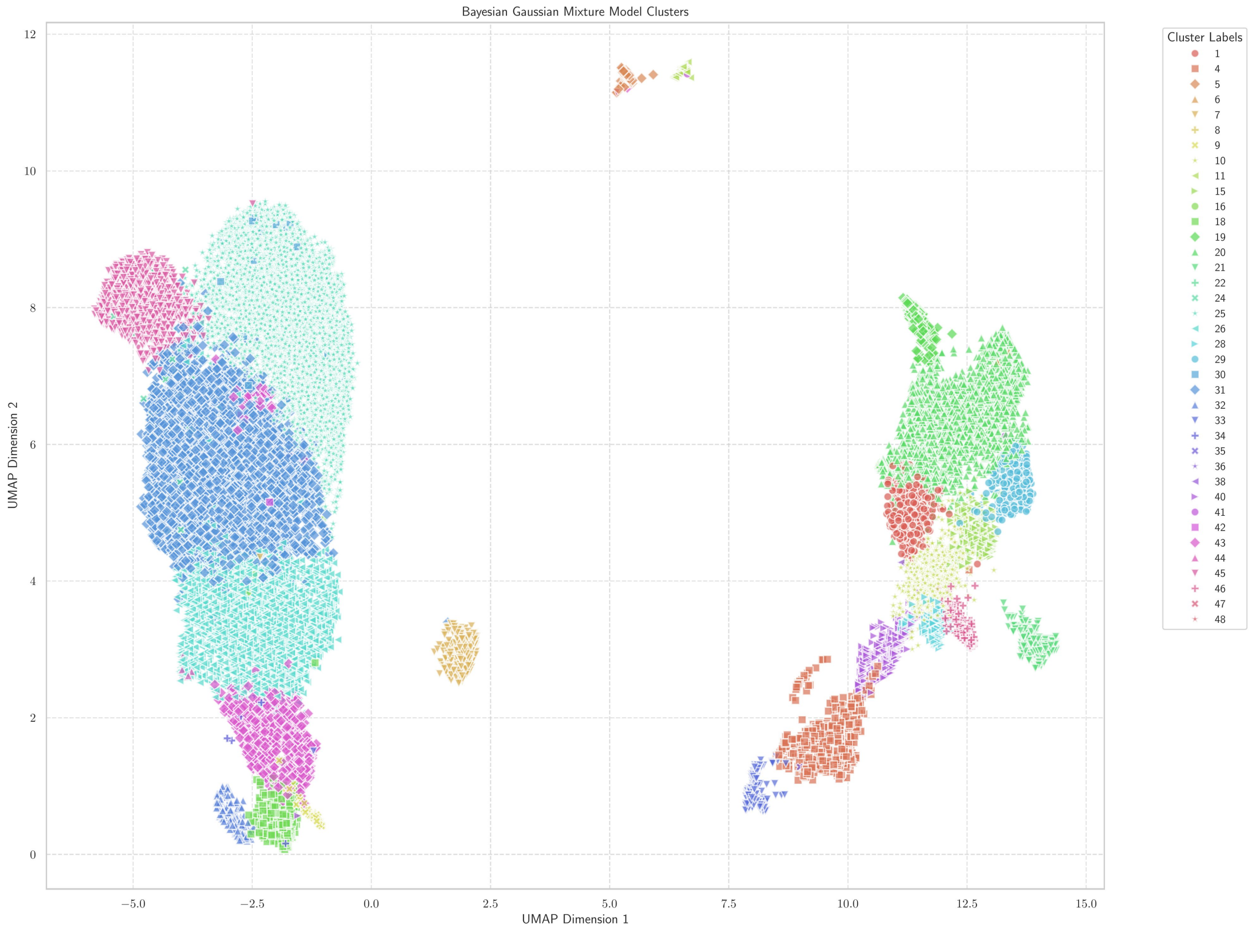

Figure S32: Foraminifera - BGMM

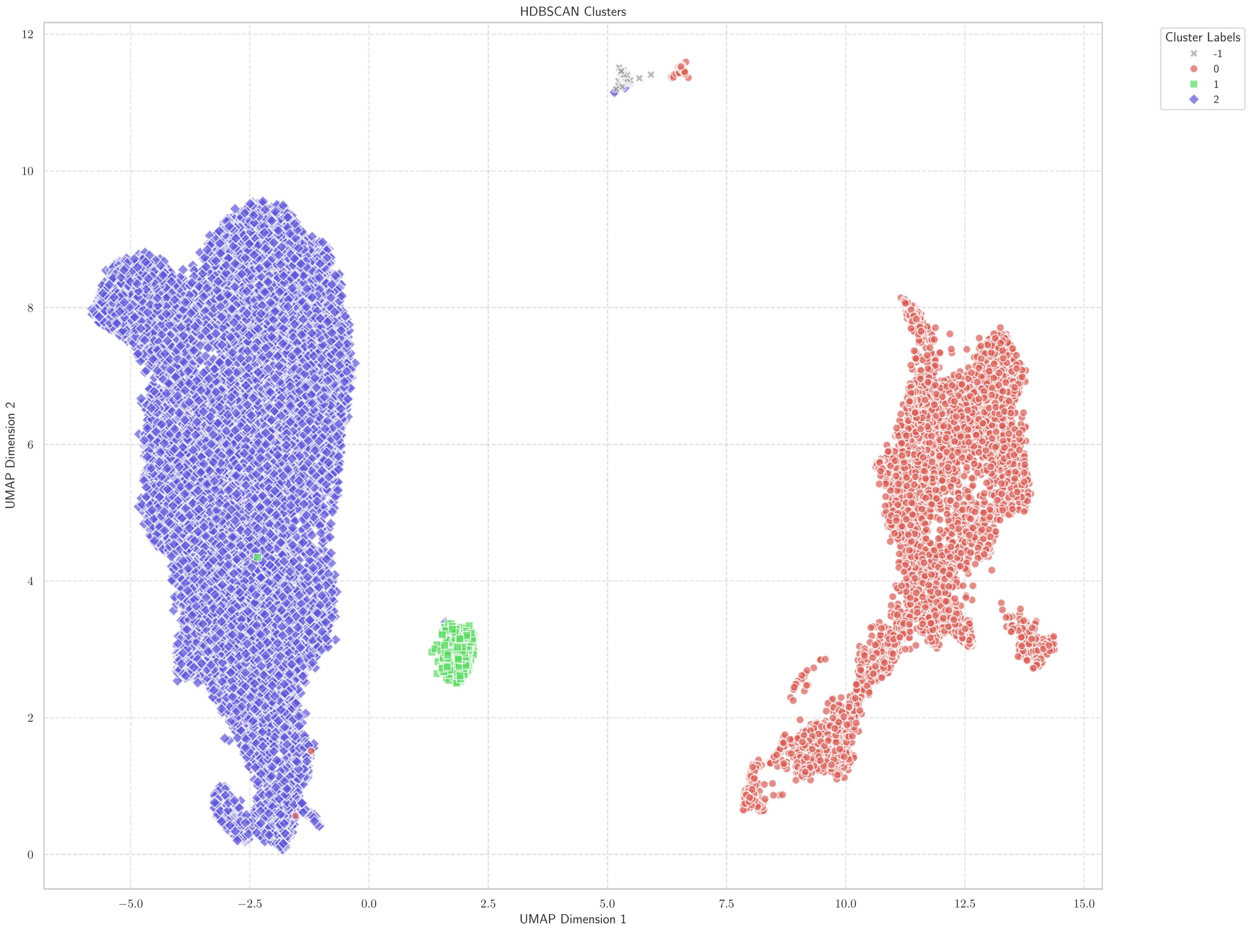

Figure S33: Foraminifera - HDBSCAN

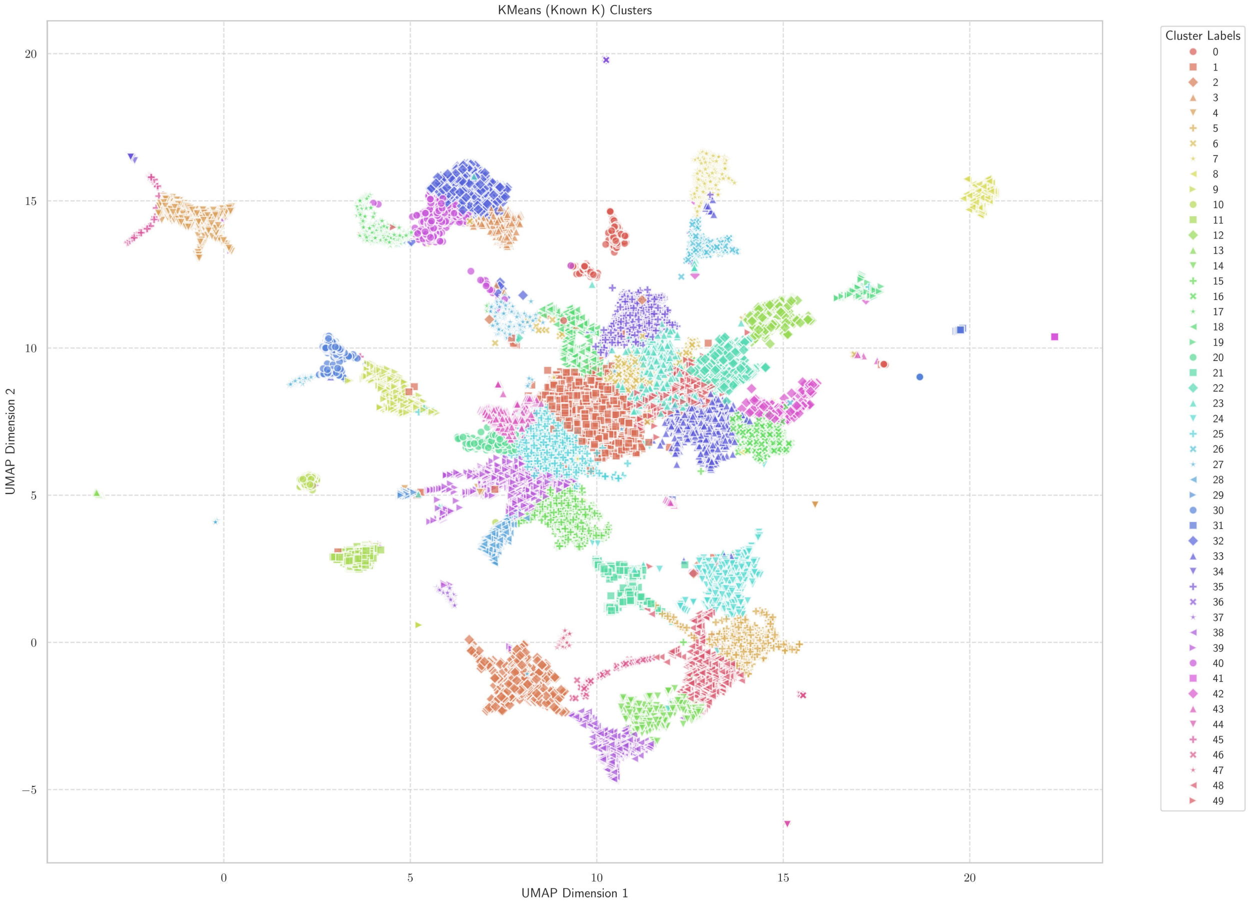

Figure S34: RFID - (known k) k-Means

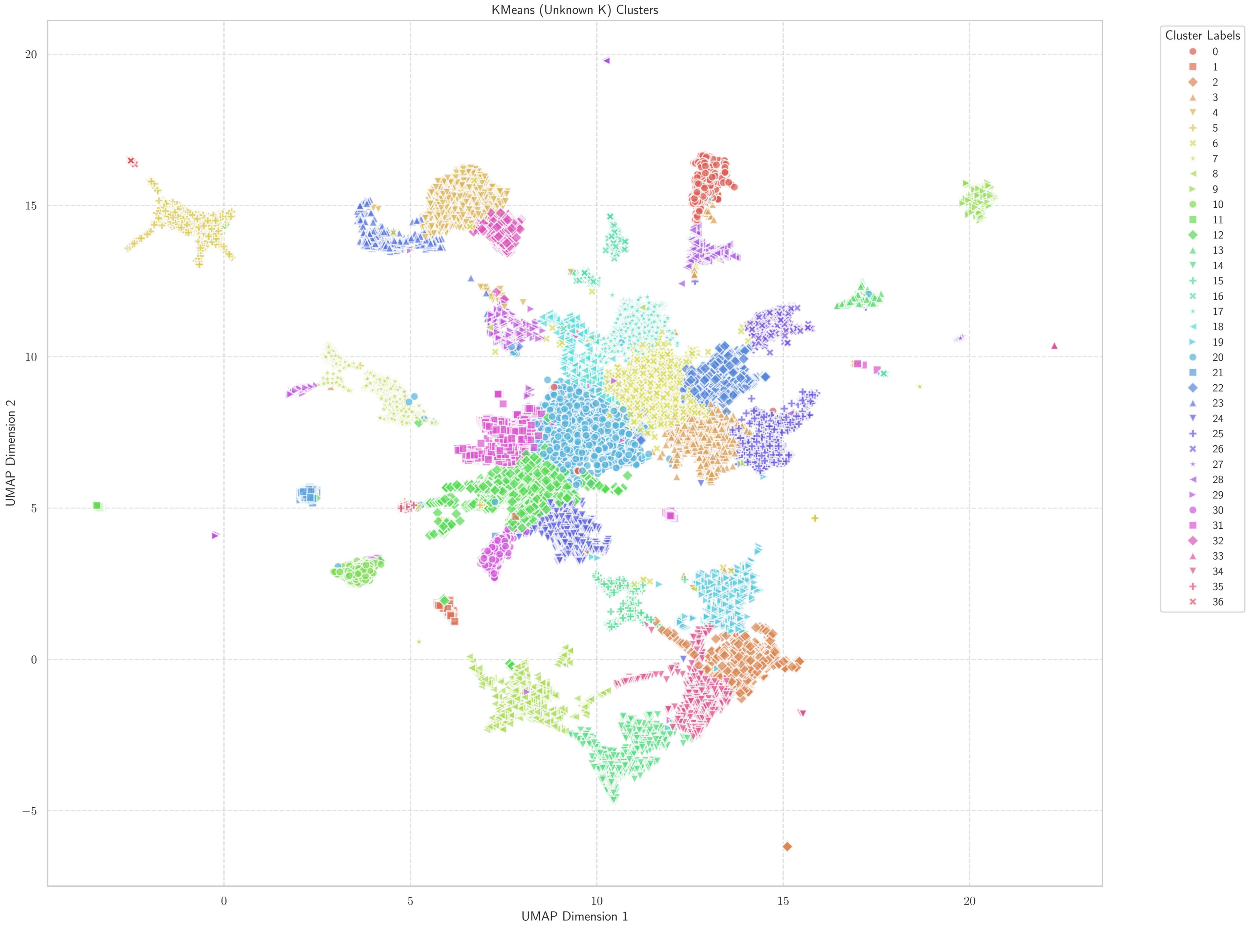

Figure S35: RFID - (unknown k) k-Means

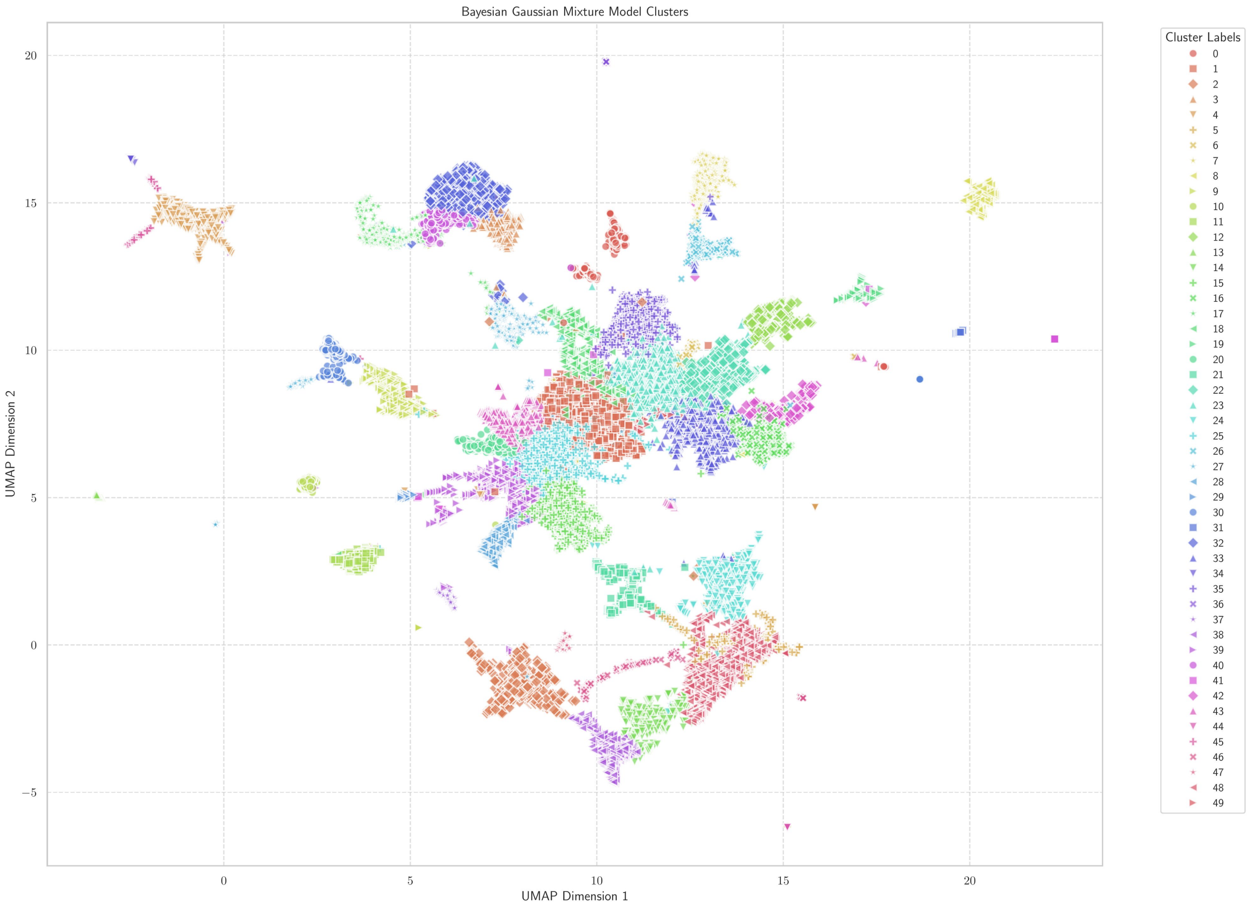

Figure S36: RFID - BGMM

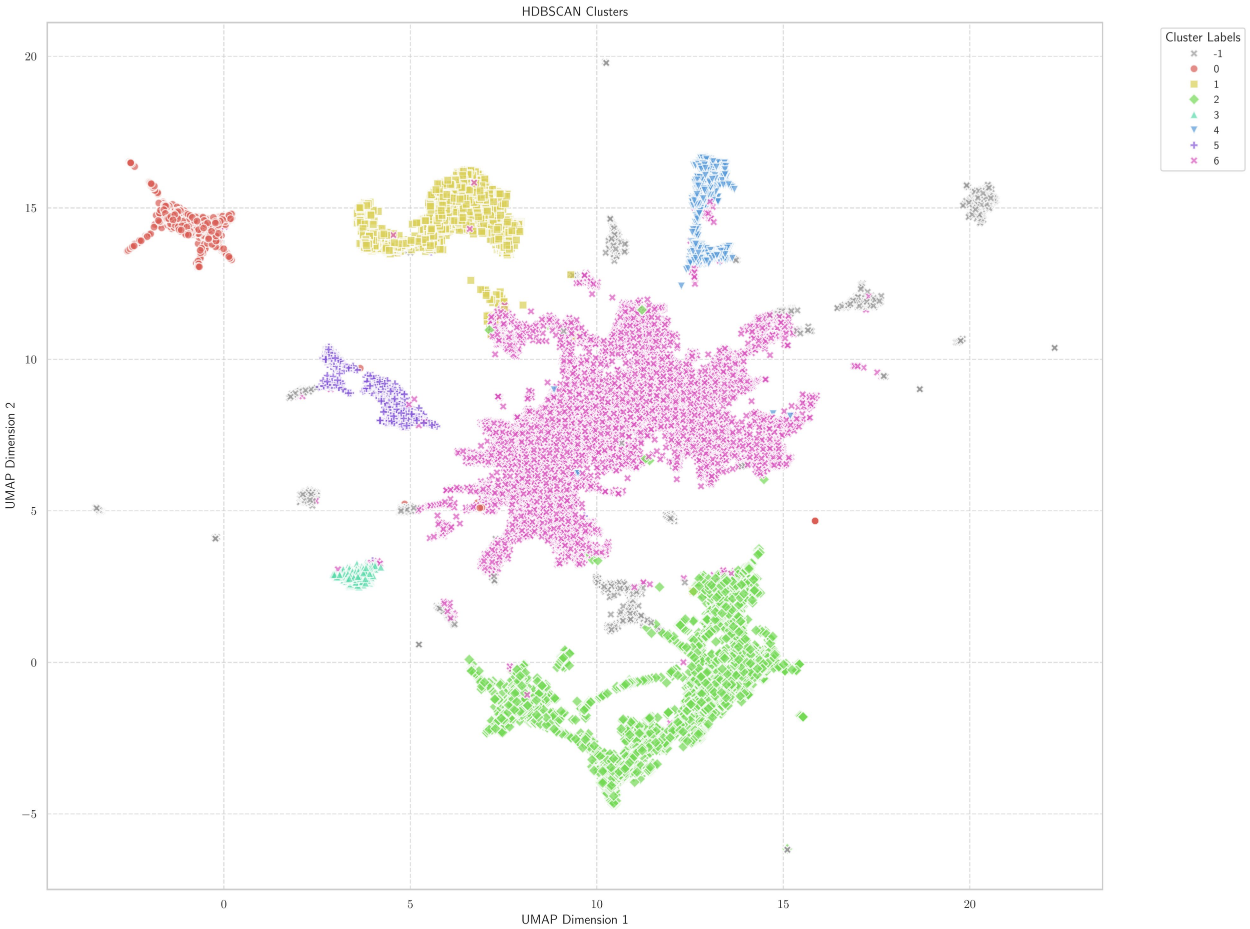

Figure S37: RFID - HDBSCAN

Supplementary References

1. Adaimé MÉ, Kong S, Urban MA, Street-Perrott FA, Verschuren D, and Punyasena SW. Reconstructing the diversity dynamics of paleo-grasslands using deep learning on superresolution images of fossil Poaceae pollen. bioRxiv 2024 Sep. DOI: 10.1101/2024.09.23.612957
2. Caron M, Touvron H, Misra I, Jegou H, Mairal J, Bojanowski P, and Joulin A. Emerging Properties in Self-Supervised Vision Transformers. *Proceedings of the IEEE International Conference on Computer Vision.* 2021. DOI: 10.1109/ICCV48922.2021.00951
3. Oquab M, Darcet T, Moutakanni T, Vo H, Szafraniec M, Khalidov V, Fernandez P, Haziza D, Massa F, El-Nouby A, Assran M, Ballas N, Galuba W, Howes R, Huang PY, Li SW, Misra I, Rabbat M, Sharma V, Synnaeve G, Xu H, Jegou H, Mairal J, Labatut P, Joulin A, and Bojanowski P. DINOv2: Learning Robust Visual Features without Supervision. arXiv:2304.07193 2024 Feb
4. Darcet T, Oquab M, Mairal J, and Bojanowski P. Vision Transformers Need Registers. arXiv:2309.16588 2024 Apr
5. Siméoni O, Vo HV, Seitzer M, Baldassarre F, Oquab M, Jose C, Khalidov V, Szafraniec M, Yi S, Ramamonjisoa M, Massa F, Haziza D, Wehrstedt L, Wang J, Darcet T, Moutakanni T, Sentana L, Roberts C, Vedaldi A, Tolan J, Brandt J, Couprie C, Mairal J, Jégou H, Labatut P, and Bojanowski P. DINOv3. arXiv:2508.10104 2025 Aug
6. Wolf T, Debut L, Sanh V, Chaumond J, Delangue C, Moi A, Cistac P, Rault T, Louf R, Funtowicz M, Davison J, Shleifer S, Platen P von, Ma C, Jernite Y, Plu J, Xu C, Scao TL, Gugger S, Drame M, Lhoest Q, and Rush AM. HuggingFace's Transformers: State-of-the-art Natural Language Processing. arXiv:1910.03771v5 2020 Jul
7. Pedregosa F, Varoquaux G, Gramfort A, Michel V, Thirion B, Grisel O, Blondel M, Prettenhofer P, Weiss R, Dubourg V, Vanderplas J, Passos A, Cournapeau D, Brucher M, Perrot M, and Duchesnay É. Scikit-learn: Machine learning in Python. Journal of Machine Learning Research 2011; 12
8. McInnes L, Healy J, Saul N, and Großberger L. UMAP: Uniform Manifold Approximation and Projection. Journal of Open Source Software 2018; 3. DOI: 10.21105/joss.00861
9. McInnes L, Healy J, and Astels S. hdbscan: Hierarchical density based clustering. The Journal of Open Source Software 2017; 2. DOI: 10.21105/joss.00205
10. Healy J and McInnes L. Uniform manifold approximation and projection. Nature Reviews Methods Primers 2024 Nov; 4:82. DOI: 10.1038/s43586-024-00363-x
11. Sevillano V, Holt K, and Aznarte JL. Precise automatic classification of 46 different pollen types with convolutional neural networks. PLoS ONE 2020; 15. DOI: 10.1371/journal.pone.0229751
12. Carlsson V, Danelian T, Boulet P, Devienne P, Laforge A, and Renaudie J. Artificial intelligence applied to the classification of eight middle Eocene species of the genus Podocyrtis (polycystine radiolaria). Journal of Micropalaeontology 2022 Nov; 41:165–82. DOI: 10.5194/jm-41-165-2022
13. Lallensack JN, Romilio A, and Falkingham PL. A machine learning approach for the discrimination of theropod and ornithischian dinosaur tracks. Journal of the Royal Society Interface 2022; 19. DOI: 10.1098/rsif.2022.0588
14. Liu X, Jiang S, Wu R, Shu W, Hou J, Sun Y, Sun J, Chu D, Wu Y, and Song H. Automatic taxonomic identification based on the Fossil Image Dataset (> 415,000 images) and deep convolutional neural networks. Paleobiology 2023; 49. DOI: 10.1017/pab.2022.14
15. Hsiang AY, Brombacher A, Rillo MC, Mleneck-Vautravers MJ, Conn S, Lordsmith S, Jentzen A, Henehan MJ, Metcalfe B, Fenton IS, Wade BS, Fox L, Meilland J, Davis CV, Baranowski U, Groeneveld J, Edgar KM, Movellan A, Aze T, Dowsett HJ, Miller CG, Rios N, and Hull PM. Endless Forams: >34,000 Modern Planktonic Foraminiferal Images for Taxonomic Training and Automated Species Recognition Using Convolutional Neural Networks. Paleoceanography and Paleoclimatology 2019; 34. DOI: 10.1029/2019PA003612
16. Marchant R, Tetard M, Pratiwi A, Adebayo M, and De Garidel-Thoron T. Automated analysis of foraminifera fossil records by image classification using a convolutional neural network. Journal of Micropalaeontology 2020; 39. DOI: 10.5194/jm-39-183-2020

17. Karaderi T, Burghardt T, Hsiang AY, Ramaer J, and Schmidt DN. Visual Microfossil Identification via Deep Metric Learning. *Pattern Recognition and Artificial Intelligence*. Ed. by El Yacoubi M, Granger E, Yuen PC, Pal U, and Vincent N. Cham: Springer International Publishing, 2022 :34–46
18. Marchant R, Tetard M, Pratiwi A, Adebayo M, and Garidel-Thoron T de. Endless Foram, MD022508 and MD9712138 training datasets. Zenodo 2020 Aug. DOI: 10.5281/zenodo.3996436. Available from: https://doi.org/10.5281/zenodo.3996436
